# A programmable DNA origami nanosyringe for directed membrane translocation

**DOI:** 10.1038/s41565-026-02249-3

**Published:** 2026-08-11

**Authors:** Longjiang Ding, Sisi Fan, Xiang Hao, Xinxin Jing, Jiahui Liu, Andreas Peil, Pengfei Zhan, Dechang Li, Stephan Nussberger, Na Liu

**Affiliations:** 1 2nd Physics Institute, University of Stuttgart, Stuttgart, Germany; 2 Max Planck Institute for Solid State Research, Stuttgart, Germany; 3 Department of Biophysics, Institute of Biomaterials and Biomolecular Systems, University of Stuttgart, Stuttgart, Germany; 4 Institute of Biomechanics and Applications, Department of Engineering Mechanics, Zhejiang University, Hangzhou, China; 5 Hangzhou Institute of Medicine, Chinese Academy of Sciences, Hangzhou, China

**Keywords:** DNA nanotechnology, Biomaterials

## Abstract

Bacterial contractile injection systems provide a model for membrane penetration and targeted delivery of molecular cargo through mechanical actuation. Replicating these features in synthetic nanoscale systems remains challenging, particularly with respect to coupling structural organization with dynamic actuation, reversibility and spatiotemporal regulation. Here we report a DNA origami nanosyringe that integrates these capabilities to enable programmable membrane translocation. The DNA origami nanosyringe comprises two ~70-nm DNA origami bundles crosslinked by a ~10-nm gold nanoparticle. One bundle forms a cholesterol-functionalized membrane-anchoring base, whereas the other serves as a DNA fuel-driven sliding needle. After binding to supported lipid bilayers or vesicle membranes, DNA-fuel actuation drives the needle downwards in ~14-nm steps, thereby enabling membrane penetration, while reverse actuation retracts the needle and promotes membrane resealing. We show that this device provides controllable delivery of cargo tethered to the needle tip into lipid-bounded compartments and can regulate biochemical processes within cell-sized environments, including membrane-localized hybridization chain reactions, RNA transcription and catalytic RNA cleavage. These results demonstrate a strategy for constructing dynamic DNA devices that operate at membrane interfaces and coordinate mechanical actuation with biochemical function.

## Abstract (web summary)

A membrane-anchoring nanodevice that integrates reversible, fuel-driven mechanical actuation enables programmable membrane penetration, cargo delivery and spatiotemporal regulation of biochemical reactions in synthetic cells.

## Main

Nature has evolved sophisticated mechanical systems for transmembrane delivery that bypass biochemical transport constraints through direct physical penetration^1^. A canonical example is the extracellular contractile injection system (CIS), a syringe-like protein-based nanomachine employed by bacteria to puncture target cells and inject effector proteins via rapid sheath contraction^2,3^. Recently, this system was repurposed for efficient protein delivery into mammalian cells through membrane penetration, underscoring the potential of mechanobiological adaptations for therapeutic and biotechnological applications^4,5^.

Despite their functional sophistication, natural molecular machines are inherently difficult to engineer due to their structural intricacy and incompletely understood activation mechanisms, limiting their controllability and adaptability. By contrast, synthetic systems, although not yet matching the precision and efficiency of biological counterparts, offer unique advantages in modularity and rational design^6^. A prominent example is intracytoplasmic sperm injection (ICSI)^7^, where a microneedle penetrates the oolemma to deliver sperm, bypassing natural fertilization barriers. While such microscale tools are well established, constructing analogous nanoscale devices capable of interacting with membranes remains a formidable challenge, particularly in achieving structural precision and functional integration. Recent advances in structural DNA nanotechnology have begun to bridge this gap, enabling highly programmable nanodevices that combine biological inspiration with synthetic innovation^8^. These include DNA-based motors^9^, turbines^10^, nanosprings^11^, force-generating actuators^12^, nanoscopic force clamps^13^ and tension sensors^14^, as well as synthetic nanosystems that generate, transmit and measure mechanical forces^15^. In addition, DNA nanostructures have been employed in membrane bioengineering to remodel lipid bilayers^16–18^, facilitate molecular transport^19,20^ and mediate signal transduction^21^. These developments provide a foundation for designing programmable nanoscale systems that integrate mechanobiological functions with precise control over membrane dynamics^15,22^.

Here, we demonstrate actuated and programmable DNA origami nanosyringes (DOSs) for directed translocation across membranes. The DOS binds to the lipid membrane via its cholesterol-tagged base, while its DNA fuel-triggered needle slides downwards to puncture the membrane, enabling high spatiotemporal control of molecular transport. Small molecules, such as ions, diffuse passively through the central channels of the needle, whereas larger biomolecules, such as DNA molecules, can be specifically tethered to the needle tip and actively translocated by direct physical penetration. This system allows on-demand initiation of biochemical processes in synthetic cells, including membrane-localized hybridization chain reaction (HCR), RNA transcription and catalytic RNA cleavage. By penetrating the membrane to enable programmable regulation of permeability, the modular DOS needle recapitulates the membrane-penetration principle of CIS. Our work establishes a transformative approach, in which DNA-based nanodevices are engineered to execute complex, highly specific functions at cellular interfaces, potentially redefining molecular delivery for diagnostics, therapeutics and synthetic biology.

## Structure and function of DOSs

**Fig. 1:**
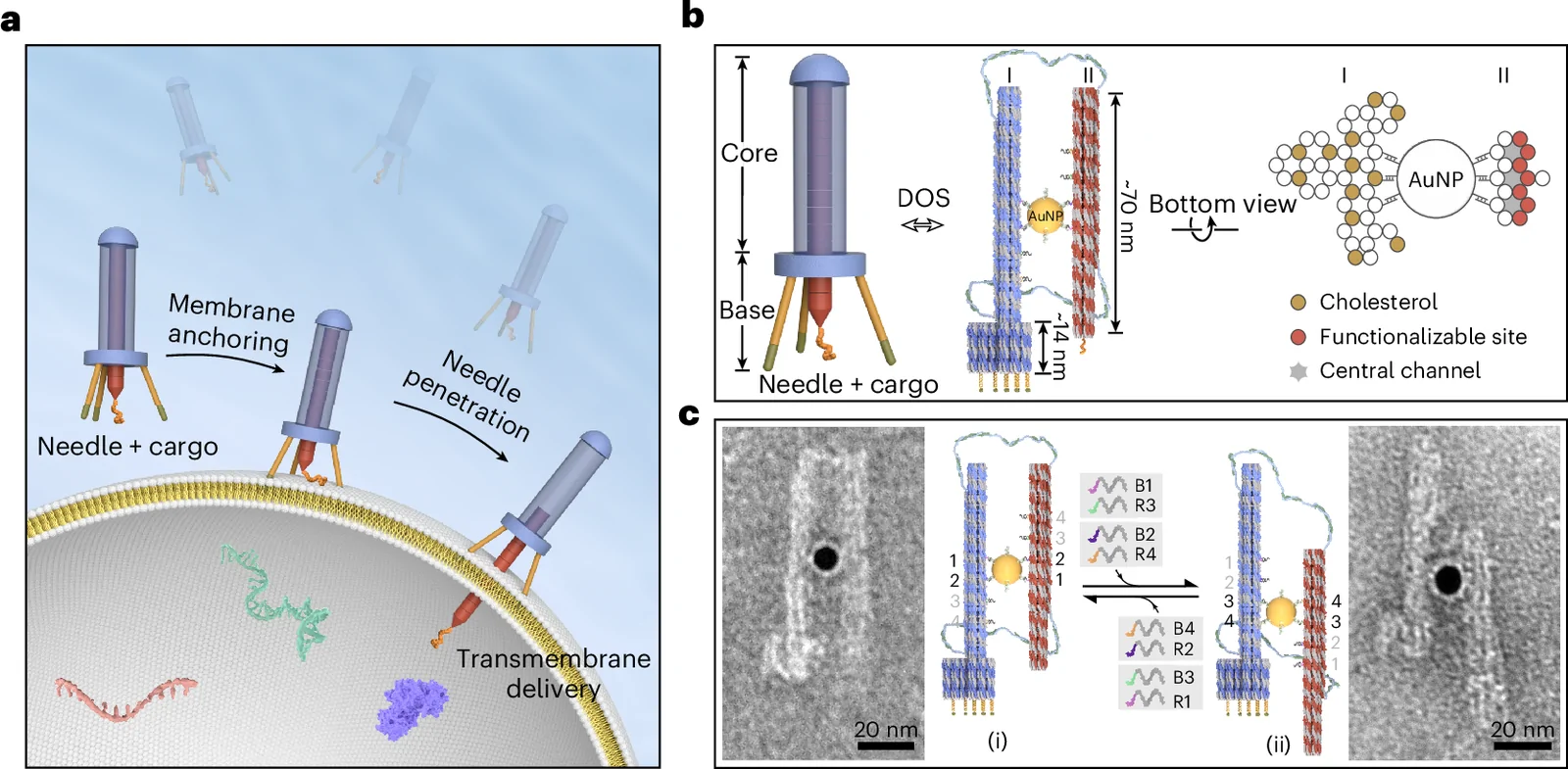
Structure and function of the DOS. **a**, Conceptual illustration of the DOS’s operation on lipid membranes, highlighting two key steps: membrane anchoring and needle penetration. **b**, Schematic of the DOS architecture. The core consists of two antiparallel, 14-helix DNA origami bundles crosslinked by a 10-nm AuNP. The bottom of bundle I is appended with a multihelix, cholesterol-modified base for membrane binding. Bundle II features seven functionalizable sites at its bottom for cargo conjugation and serves as the needle, enclosing three ~2-nm central channels arranged in a honeycomb lattice. The scaffold strand passing through both bundles reinforces structural alignment, while preserving flexibility. Four rows of footholds are positioned along the inner surface of each bundle. **c**, AuNP-mediated reversible sliding between the two bundles, activated by toehold-mediated strand displacement upon the addition of DNA fuel strands. The AuNP binds to two foothold rows on each bundle, maintaining antiparallel alignment. Sliding is initiated by the simultaneous addition of specific DNA fuel strands. Blocking strands (B1–B4) release the AuNP from bound footholds, while removal strands (R1–R4) reactivate previously deactivated footholds, enabling AuNP binding. TEM images: (i) before and (ii) after two-step sliding.

**Extended Data Fig. 1:**
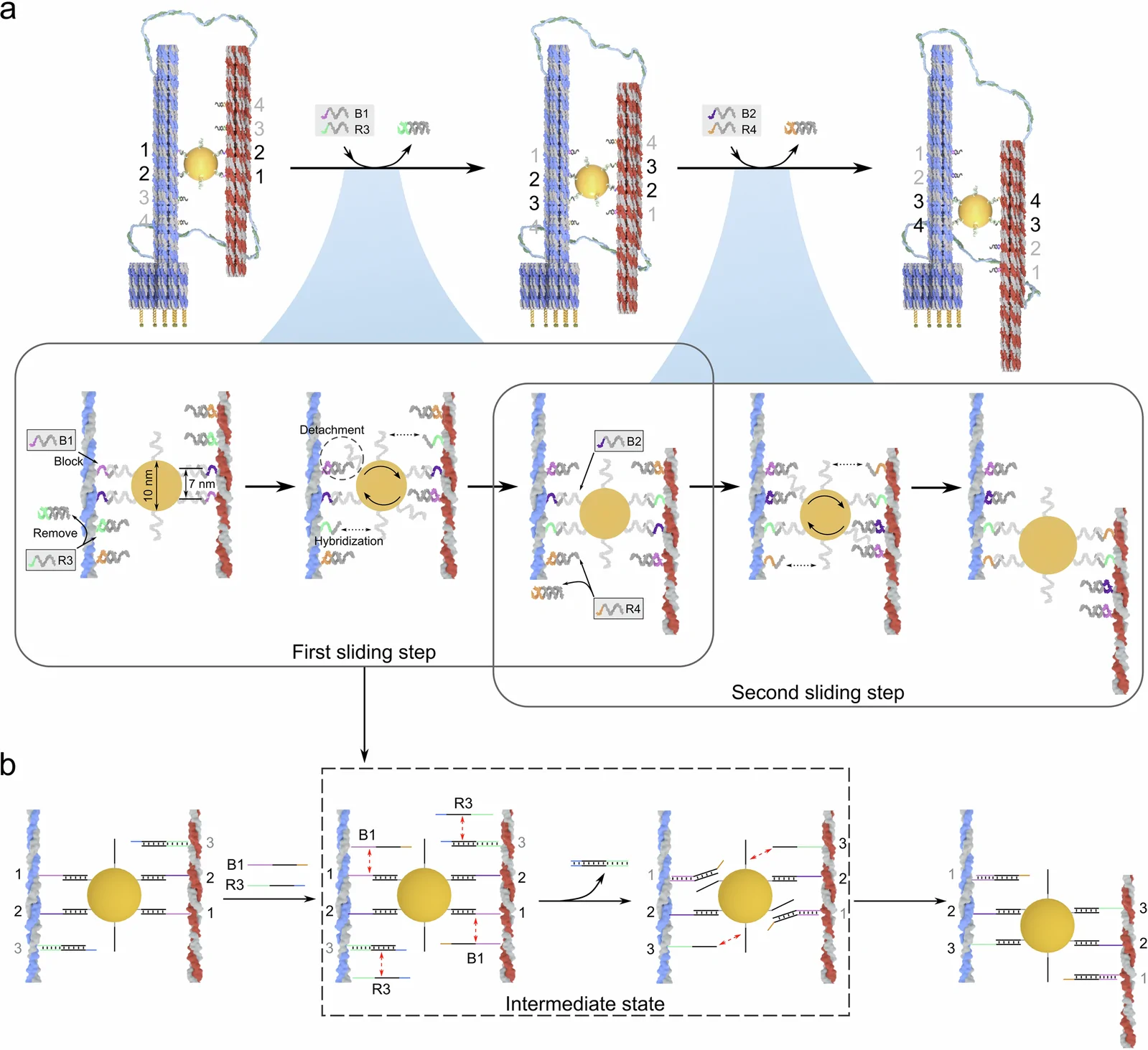
Directional sliding mechanism of the DOS via toehold-mediated strand displacement. **a**, Schematic illustrating the sliding mechanism. The AuNP (~10 nm) crosslinks two antiparallel DNA origami bundles, each containing four rows of footholds (1-4) spaced ~7 nm apart. The foothold rows are arranged in reverse order on the two bundles, giving them opposite polarities. Only two central foothold rows on each bundle (rows 1 and 2) are initially engaged by the AuNP, while the remaining rows are passivated. Upon addition of blocking and removal strands, toehold-mediated strand displacement reactions detach the AuNP from one foothold row and activate an adjacent row, thereby preventing back-sliding and enforcing directional movement. Specifically, blocking strands B1 displace the AuNP from row 1, while removal strands R3 activate row 3 for hybridization, resulting in a shift of the AuNP binding site and a relative displacement of the bundles by 14 nm. A second step, induced by B2 and R4, produces an additional 14 nm displacement. **b**, Detailed schematic of the strand displacement process during a sliding step. Sequence domains on the DNA origami, AuNP-bound strands, and fuel strands are indicated. Blocking strands displace AuNP-bound strands from occupied footholds, while removal strands activate new foothold sites for binding. This drives relocation of the AuNP and directional sliding of the DNA needle, enabling the needle to advance across the membrane and adopt a membrane-spanning configuration.

Drawing inspiration from the CIS (Fig. 1a), the DOS is constructed from modular components with well-defined functions (Fig. 1b). Its core structure comprises two 14-helix DNA origami bundles (I and II, ~70 nm each), crosslinked by a 10-nm gold nanoparticle (AuNP). The scaffold strand passing through both bundles reinforces structural alignment, while preserving flexibility (Supplementary Figs. 1 and 2). Four rows of footholds, spaced at ~7-nm intervals and oriented oppositely, are arranged on the inner surfaces of the two bundles (Supplementary Fig. 3). The AuNP binds to two foothold rows on each bundle with high efficiency (~91%), organizing them in an antiparallel configuration, while the remaining footholds are passivated (Supplementary Figs. 4 and 5). DNA fuel strands initiate reversible sliding between the bundles via toehold-mediated strand displacement. This process is controlled by blocking and removal strands, which detach the AuNP from one foothold row and activate an adjacent row for binding^23,24^ (Fig. 1c). This stepwise relocation of the AuNPs enables directional sliding of the bundles relative to one another (Extended Data Fig. 1). Each sliding step results in a 14-nm displacement, with a maximum shift of 28 nm over two steps. Transmission electron microscopy (TEM) images confirm distinct DOS configurations before and after two sliding steps (Fig. 1c and Supplementary Fig. 6). Unless stated otherwise, all experiments are performed under the two-step sliding condition.

A 14-nm multihelix segment is appended to bundle I to form the DOS base, with 12 cholesterol-tagged DNA anchors spatially distributed on its underside. These anchors facilitate membrane binding of the DOS without inducing aggregation, as indicated by the monodisperse distribution of DOSs (Supplementary Fig. 5). Bundle II, termed the needle, adopts a honeycomb lattice arrangement of DNA helices, enclosing three central channels (each ~2 nm in diameter)^25^. The needle tip is equipped with seven functionalizable sites for cargo conjugation. Once the base is anchored to the membrane, the needle undergoes DNA-fuel-triggered sliding, driving it downwards to pierce the membrane. This design supports two distinct translocation modes: its central channels permit passive diffusion of small molecules, while its tip enables directed transport of tethered macromolecular cargo across the membrane. Subsequent upward sliding retracts the needle, restoring membrane integrity.

## Membrane perforation via downward needle sliding

**Fig. 2:**
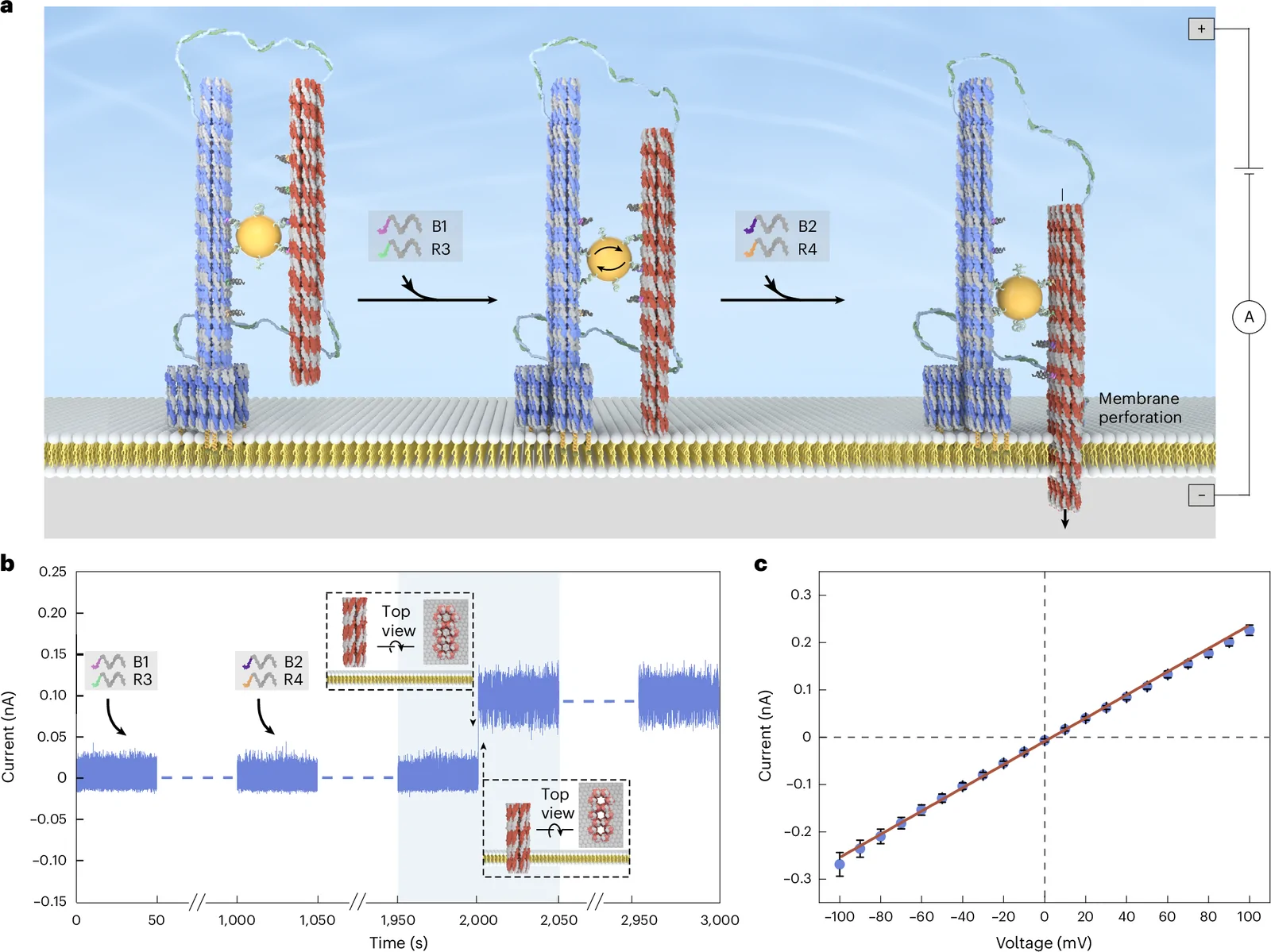
Membrane perforation of supported lipid bilayers by nanosyringe. **a**, Schematic of membrane perforation by sliding-induced needle penetration through a supported lipid bilayer. **b**, A representative continuous current recording after addition of DNA fuel strands. Current monitoring starts upon addition of fuel strands B1/R3 at *t* = 0 min, followed by addition of B2/R4 at *t* = 15 min. A stepwise current increase is observed at approximately *t* = 33 min, indicating the formation of a conductive pore. **c**, Current trace recorded during voltage ramps from −100 mV to +100 mV in 10-mV increments. Data represent the mean ± s.d. from six individual insertion events. All electrophysiological measurements are conducted in 1 M KCl and 10 mM HEPES at pH 7.6. Source data

**Extended Data Fig. 2:**
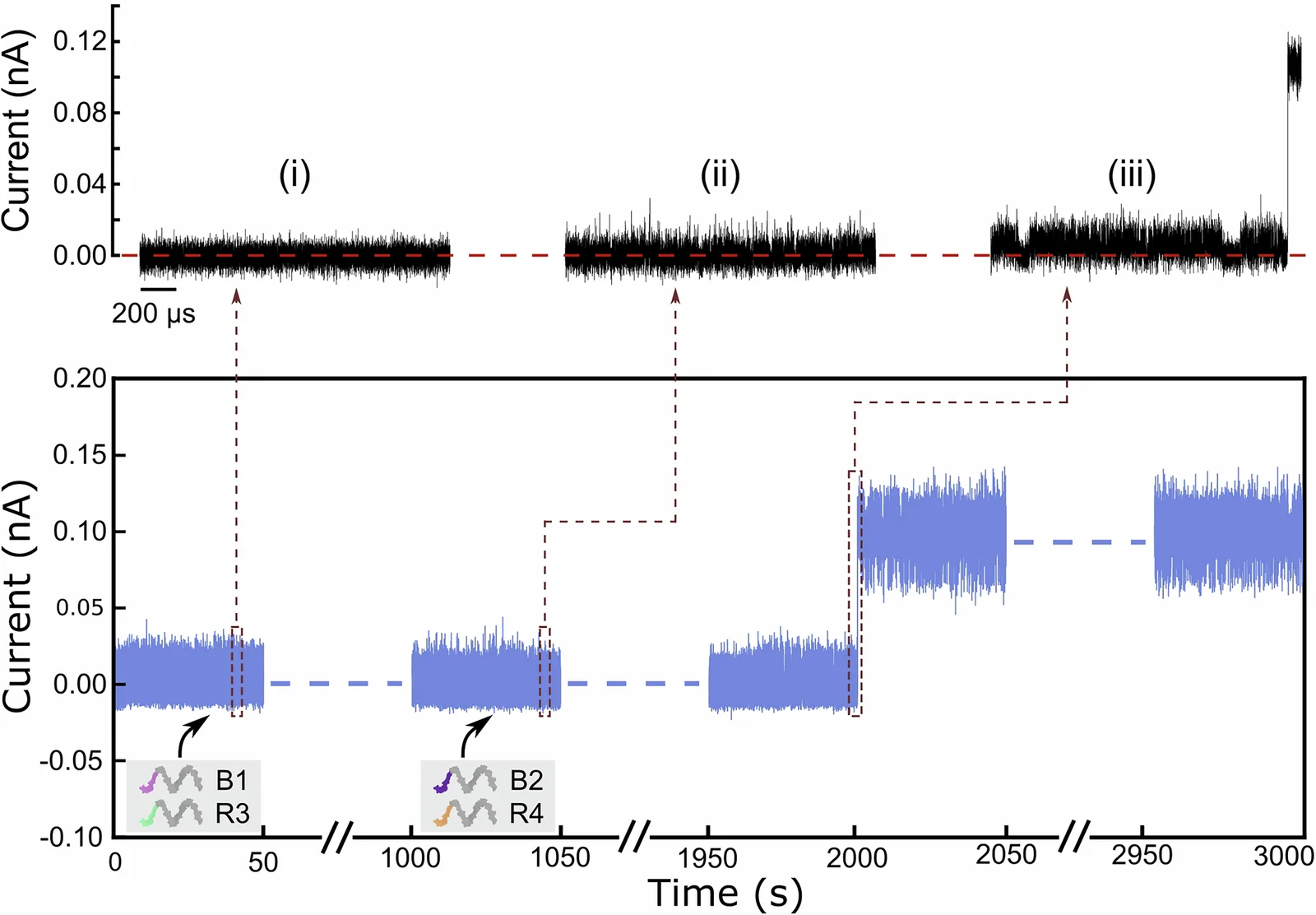
Time-resolved current recording during DOS needle penetration. (i) Stable current following addition of fuel strands B1/R3. (ii) Increased baseline noise after addition of fuel strands B2/R4, indicating initial needle-membrane interactions. (iii) Transient fluctuations preceding a stable current step, suggesting the formation of short-lived membrane defects and subsequent conductive pore formation. The measurement was conducted in 1 M KCl and 10 mM HEPES (pH 7.6) at an applied membrane potential of 50 mV.

To investigate the membrane perforation process by DOSs, single-channel current recordings are conducted across free-standing lipid bilayers (Fig. 2a). Ionic currents are monitored over time at a transmembrane potential of +50 mV. DOS binding to the membrane alone yields no detectable current, confirming preservation of bilayer integrity. Following addition of fuel strands, a stepwise current increase is observed, indicative of conductive pore formation (Fig. 2b). In the absence of AuNPs, DNA origami structures alone do not exhibit detectable current signals under the same conditions (Supplementary Fig. 7). These results demonstrate that membrane perforation is directly driven by downward sliding of the needle. Through time-resolved current analysis, these results indicate that DNA strand displacement, needle–membrane interactions and defect formation together contribute to the observed timescale of membrane perforation (Extended Data Fig. 2). The onset kinetics of membrane perforation are comparable to those of natural transmembrane proteins, such as outer membrane protein F (OpmF)^26^ and aerolysin^27^ (Supplementary Fig. 8), suggesting that this engineered system recapitulates the temporal characteristics of biological pores. The needle-punctured pore remains stable for over 1 h under continuous conduction. Furthermore, the linear current–voltage relationship supports a symmetric transmembrane conductive path, consistent with the architecture of the needle’s central channels^28,29^ (Fig. 2c and Supplementary Fig. 9).

## Nanosyringe operation on synthetic cells

**Fig. 3:**
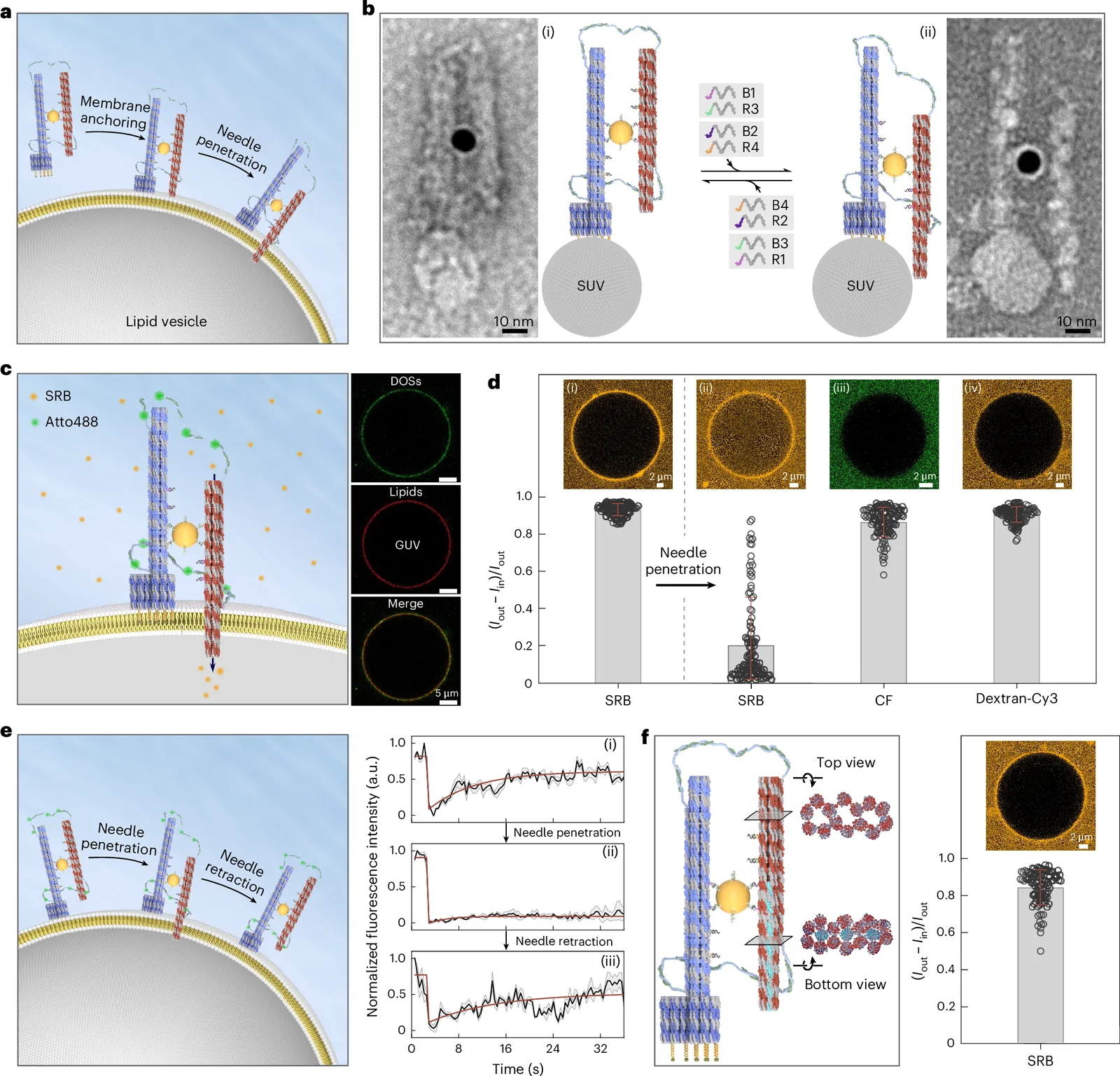
Needle penetration and retraction of nanosyringe on lipid vesicles. **a**, Schematic of membrane anchoring and needle penetration by the DOS on a lipid vesicle. **b**, Schematics and representative TEM images of DOSs vertically anchored on SUVs, shown (i) before and (ii) after sliding. **c**, Schematic and confocal fluorescence images of Atto488-labelled DOSs bound to GUVs, with SRB used as an influx indicator. **d**, Quantification of the normalized fluorescence intensity difference, (*I*_out_ − *I*_in_)/*I*_out_, of GUVs at equilibrium exposed to (i) SRB, (ii) CF and (iii) dextran-Cy3. Data represent mean ± s.d. from three independent experiments. Number of GUVs analysed: *n* = 108 for SRB before and after needle penetration; *n* = 112 for CF and dextran-Cy3 after needle penetration. **e**, Schematic of DOS needle penetration and retraction on GUVs, along with the corresponding diffusion behaviours in (i) prepenetration, (ii) penetration and (iii) retraction states, as characterized by FRAP. Red lines indicate fits to the recovery curves. Data represent mean ± s.d. (*n* = 3). **f**, Schematic and oxDNA simulation of the DOS variant with blocked needle channels, along with quantification of SRB transport following needle penetration. Data represent mean ± s.d. from three independent experiments (*n* = 104). Source data

**Extended Data Fig. 3:**
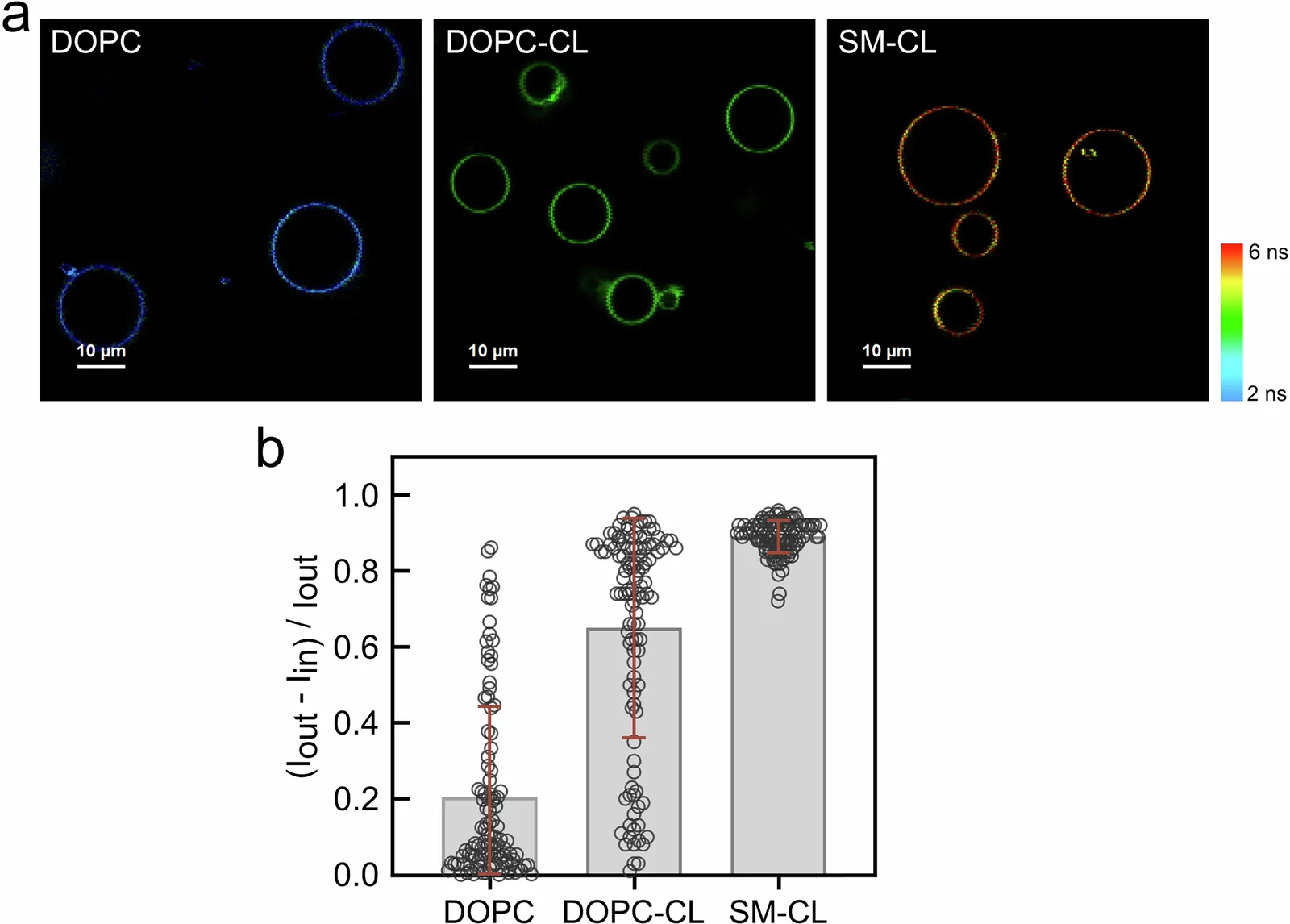
Effect of membrane mechanics on needle penetration. **a**, Fluorescence lifetime imaging of GUVs with three lipid compositions: DOPC, DOPC:cholesterol (DOPC-CL) 60:40, and sphingomyelin:cholesterol (SM-CL) 70:30 (molecular weight ratio), stained with the mechanosensitive probe Flipper-TR. Longer fluorescence lifetime indicates increased membrane mechanical resistance across the three compositions. **b**, Quantification of the normalized fluorescence intensity difference, *(I*_*out*_ *- I*_*in*_*)/I*_*out*_, of GUVs at equilibrium after exposure to SRB following needle penetration. SRB influx efficiency decreases with increasing membrane mechanical resistance, indicating reduced membrane penetration efficiency. Data represent mean ± s.d. from three independent experiments (n = 108, 113 and 111 for DOPC, DOPC-CL and SM-CL, respectively). Source data

To explore performance in a cell-mimetic setting, we apply DOSs to synthetic vesicles (Fig. 3a). DOSs are first incubated with small unilamellar vesicles (SUVs, ~50 nm) prepared via lipid-film rehydration and extrusion. Representative TEM images (Fig. 3b) reveal DOSs bound vertically on SUV membranes. Before actuation, the two bundles adopt a symmetric configuration (Fig. 3b(i) and Supplementary Fig. 10), which transitions to an asymmetric arrangement upon DNA fuel-triggered sliding (Fig. 3b(ii) and Supplementary Fig. 11). DOSs are then applied to giant unilamellar vesicles (GUVs) composed of 1,2-dioleoyl-*sn*-glycero-3-phosphocholine (DOPC). For fluorescence visualization, eight Atto488 molecules are attached to the scaffold strand at positions distal to the AuNP to minimize quenching (Fig. 3c). Confocal fluorescence microscopy imaging confirms membrane binding through colocalization of DOS and lipid signals (Fig. 3c and Supplementary Fig. 12). Membrane perforation is assayed using sulforhodamine B (SRB, ~0.7 nm)^29–31^, quantified as *I*_*n*_ = (*I*_out_ − *I*_in_)/*I*_out_, where *I*_in_ and *I*_out_ denote fluorescence intensities inside and outside the GUVs, respectively^16^. To avoid transient dye leakage during needle penetration, SRB is introduced to the GUV external space 1 h after actuation. Minimal influx is observed before actuation (<*I*_*n*_ > = 0.94; Fig. 3d(i)), whereas substantial SRB entry occurs after actuation (<*I*_*n*_ > = 0.20; Fig. 3d(ii)), confirming successful membrane perforation (Supplementary Figs. 13 and 14). By contrast, DNA origami structures alone show negligible influx under the same experimental conditions (Supplementary Fig. 15). Moreover, DOS-mediated transport exhibits charge- and size-dependent molecular selectivity. Specifically, transport of 6-carboxyfluorescein (CF) is markedly reduced compared with SRB, with replacement of a positive charge by a negative one within the same parent structure^29,32^ (Fig. 3d(iii) and Supplementary Fig. 16a). Also, 3-kDa dextran-Cy3 (~3 nm in hydrodynamic diameter) remains impermeable due to size exclusion (Fig. 3d(iv) and Supplementary Fig. 16b), consistent with the internal dimension of the needle’s central channels. These findings reveal that molecular transport occurs predominantly through the needle’s central channels, rather than via non-specific leakage or vesicle disruption. Furthermore, a DOS variant bearing two AuNPs exhibits comparable SRB influx efficiency^24^ (Supplementary Fig. 17), and SRB efflux from preloaded GUVs confirms bidirectional molecular transport (Supplementary Fig. 18). The DOS-enabled SRB influx is further examined in GUVs with different lipid compositions^33^ (Extended Data Fig. 3). Across membranes with systematically varied compositions, penetration efficiency decreases with increasing membrane mechanical resistance, indicating that membranes with higher resistance are more difficult to penetrate. These results suggest that the mechanical resistance of the membrane is a key barrier to needle insertion.

## Membrane resealing via needle retraction

Unlike conventional DNA-based transmembrane constructs that rely on hydrophobic modifications for membrane insertion^19,34^, the DOS operates based on a fundamentally distinct mechanism via directed membrane penetration. Its needle pierces the membrane via downward sliding actuation, eliminating dependence on chemical modifications. Also, while advanced DNA nanopores can be gated^35^, they typically remain in the membrane after insertion. By contrast, the DOS offers operational reversibility, as indicated by restoration of baseline current in free-standing lipid bilayers (Supplementary Fig. 19) and fluorescence recovery after photobleaching (FRAP) in GUVs (Fig. 3e and Supplementary Fig. 20), both of which are associated with needle retraction. In lipid bilayer recordings, the current returns to a stable baseline following addition of upward-sliding fuel strands, indicating needle retraction while preserving membrane integrity (Supplementary Fig. 19). In GUVs, Atto488-functionalized DOSs in the prepenetration state exhibit rapid fluorescence recovery following photobleaching (Fig. 3e(i)), reflecting high mobility. After penetration (Fig. 3e(ii)), fluorescence recovery is abolished, indicating reduced mobility. This reduced mobility is attributed to the membrane-spanning configuration of the DOS, which introduces additional anchoring, thereby restricting lateral diffusion. Subsequent retraction via fuel-triggered upward needle sliding reinstates fluorescence recovery with kinetics comparable to those of the prepenetration state (Fig. 3e(iii)), verifying physical disengagement^36^. SRB influx assays further corroborate membrane resealing, showing substantially reduced transport after retraction (Supplementary Fig. 21).

As a negative control, we also design a modified DOS, in which the lower needle segment adopts hexagonal-lattice close-packing instead of honeycomb-lattice packing^37^ (Supplementary Figs. 22 and 23), effectively occluding the three central channels as shown in the oxDNA (a simulation code implementing a coarse-grained DNA model) simulations^38^ (Fig. 3f). Corresponding ionic current recordings (Supplementary Fig. 24) and SRB influx assays (Fig. 3f and Supplementary Fig. 25) reveal that this DOS variant is nearly impermeable, validating that molecular transport occurs primarily through the central channels and peripheral membrane disruption is negligible. Furthermore, all-atom molecular dynamics simulations show preferential association of lipid headgroups with the DNA surfaces (Supplementary Fig. 26), suggesting minimal membrane disruption and local stabilization after needle penetration^36,39^. Taken together, these results demonstrate that the DOS enables programmable membrane translocation via a mechanical actuation cycle, integrating molecular precision, structural resilience and dynamic function in a single DNA nanodevice.

## Nanosyringe-directed HCR

**Fig. 4:**
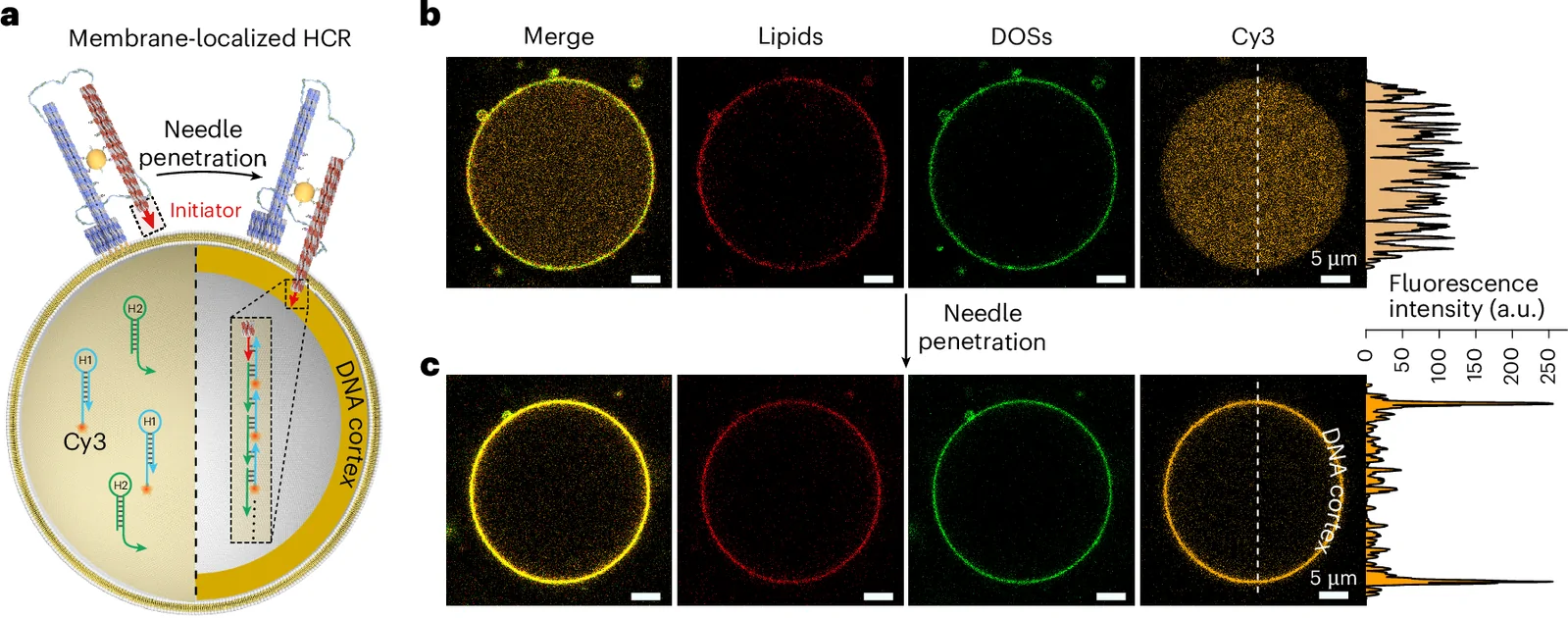
Nanosyringe-directed membrane-localized HCR in synthetic cells. **a**, Schematic illustrating spatiotemporal control of HCR within a GUV enabled by DOS needle penetration. **b**, Confocal fluorescence images before needle penetration, showing a homogeneous distribution of DNA strands within the GUV lumen. **c**, Confocal fluorescence images after needle penetration, revealing the formation of a ring-shaped DNA cortex. Fluorescence intensity profiles along the equatorial planes (Cy3 channel) are shown alongside **b** and **c**. Source data

A defining feature of the DOS is its mechanical actuation and its ability to spatiotemporally control transport across membranes. To highlight this capability, we demonstrate a membrane-localized HCR in GUVs. HCR is a DNA-based signal amplification method that utilizes specifically designed initiator strands to catalyse the formation of extended DNA polymers via sequential hybridization of hairpin oligonucleotides^40^. Two partially self-complementary hairpin oligonucleotides (Cy3-labelled H1 in blue, unlabelled H2 in green) are pre-encapsulated within GUVs as reaction substrates (Fig. 4a). These hairpins, designed with high kinetic barriers to prevent spontaneous hybridization, initially show a uniform fluorescence distribution throughout the GUV lumen (Fig. 4b and Supplementary Fig. 27). Four DNA initiator strands (red) are conjugated to the functionalizable sites at the tip of the DOS needle. Upon fuel-induced downward sliding and membrane piercing, these DNA initiators are exposed to the GUV interior, enabling their access to the encapsulated H1 and H2. The initiators first open the H1 hairpins via toehold-mediated strand displacement. This subsequently triggers an autocatalytic hybridization cascade between H1 and H2, producing long, double-stranded DNA polymers, until hairpin substrates are depleted in the GUV.

Notably, as the needle only dips into the GUV, initiator release is spatially confined near the inner membrane. This localized seeding event leads to HCR-driven polymerization concentrated along the membrane interface, forming a ring-shaped DNA cortex, as indicated by the peripheral fluorescence observed in Fig. 4c and Supplementary Fig. 27. Time-resolved and quantitative analysis of the equatorial fluorescence intensity reveals a marked redistribution of DNA, from initial homogeneity to concentrated accumulation at opposing membrane poles (Supplementary Fig. 28). This DOS-directed transmembrane activation illustrates a new scheme of intracellular reaction control, where directed translocation, spatial targeting and signal amplification converge to create dynamic and localized functional architectures within synthetic cells.

## Nanosyringe-directed RNA transcription

**Fig. 5:**
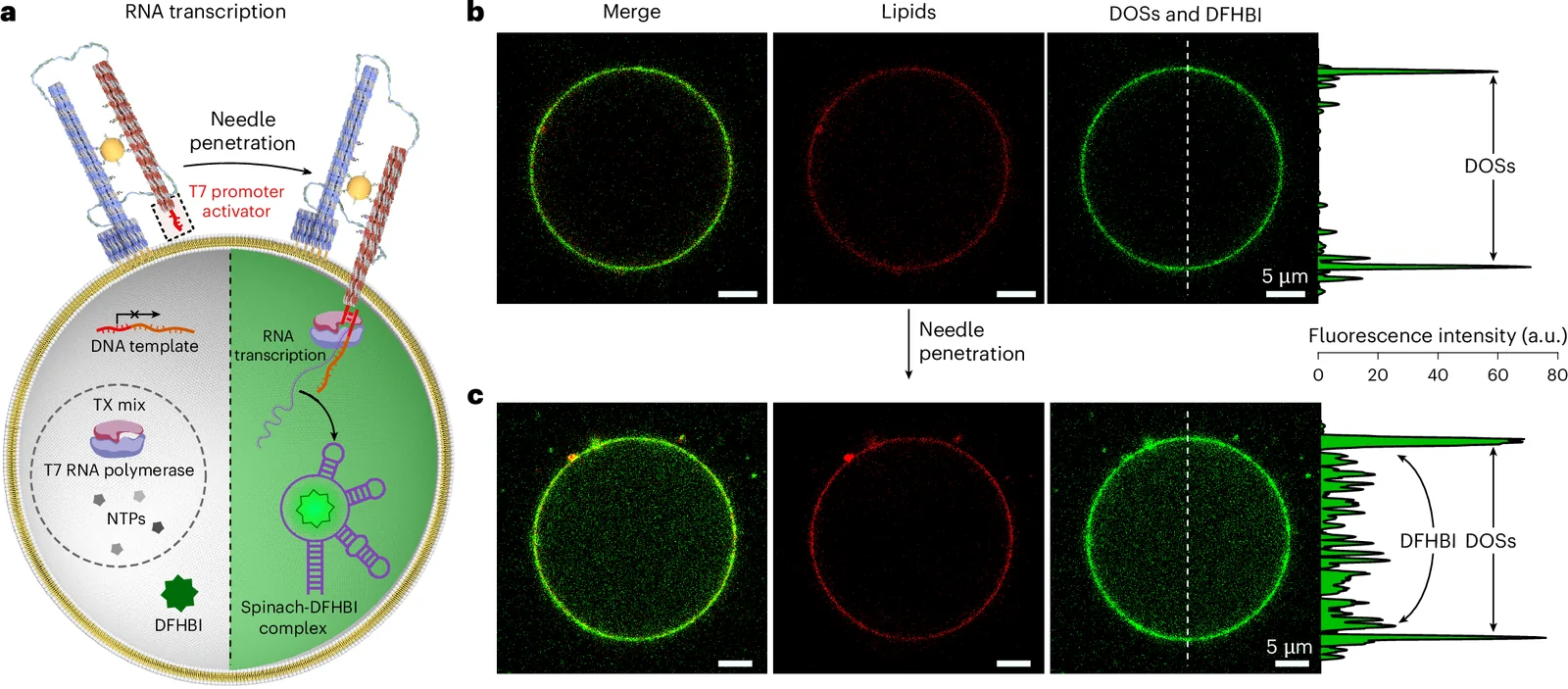
Nanosyringe-directed RNA transcription in synthetic cells. **a**, Schematic of DOS needle-guided delivery of T7 promoter activators into a GUV, leading to the formation of T7 promoter duplexes and subsequent transcription of Spinach RNA aptamers in the presence of DFHBI. **b**, Confocal fluorescence images before needle penetration. The absence of fluorescence inside the GUV indicates stringent suppression of RNA transcription. **c**, Confocal fluorescence images after needle penetration. Fluorescence signals emerge within the GUV, confirming successful transcription of the Spinach RNA aptamers. Images in **b** and **c** are acquired after 12-h incubation at 25 °C. Atto488-labelled DOSs and DFHBI are recorded in the same confocal channel. Fluorescence intensity profiles along the equatorial planes are shown alongside **b** and **c**. Source data

Controlled RNA transcription underlies both natural gene regulation and synthetic genetic circuit design^41,42^. Next, we set out to demonstrate DOS-directed activation of RNA transcription in GUVs. Specifically, we employ the Spinach RNA aptamer, which fluoresces upon binding the small-molecule fluorogen DFHBI (3,5-difluoro-4-hydroxybenzylidene imidazolinone), providing a visual readout of transcriptional activity^43^. As illustrated in Fig. 5a, T7 promoter-activating DNA sequences are anchored to the DOS needle tip, while the Spinach encoding template, DFHBI, and the transcription mixture (TX mix, containing T7 RNA polymerase, nucleoside triphosphates (NTPs) and supporting reagents) are pre-encapsulated within GUVs. Before needle penetration, the absence of fluorescence inside the GUV indicates stringent suppression of RNA transcription (Fig. 5b and Supplementary Fig. 29). Following DOS needle penetration and 12-h incubation at 25 °C, fluorescence signals emerge within the GUV, confirming successful transcription of the Spinach RNA aptamers (Fig. 5c and Supplementary Fig. 29). Time-resolved control experiments further demonstrate that transcription strictly depends on DOS-directed transport of T7 promoter activators, as omission of this component abolishes fluorescence (Supplementary Fig. 30). These results establish DOSs as a programmable nanoscale tool for externally triggered cell-free gene expression with spatiotemporal precision, while preserving compartmentalization.

## Nanosyringe-directed catalytic RNA cleavage

**Fig. 6:**
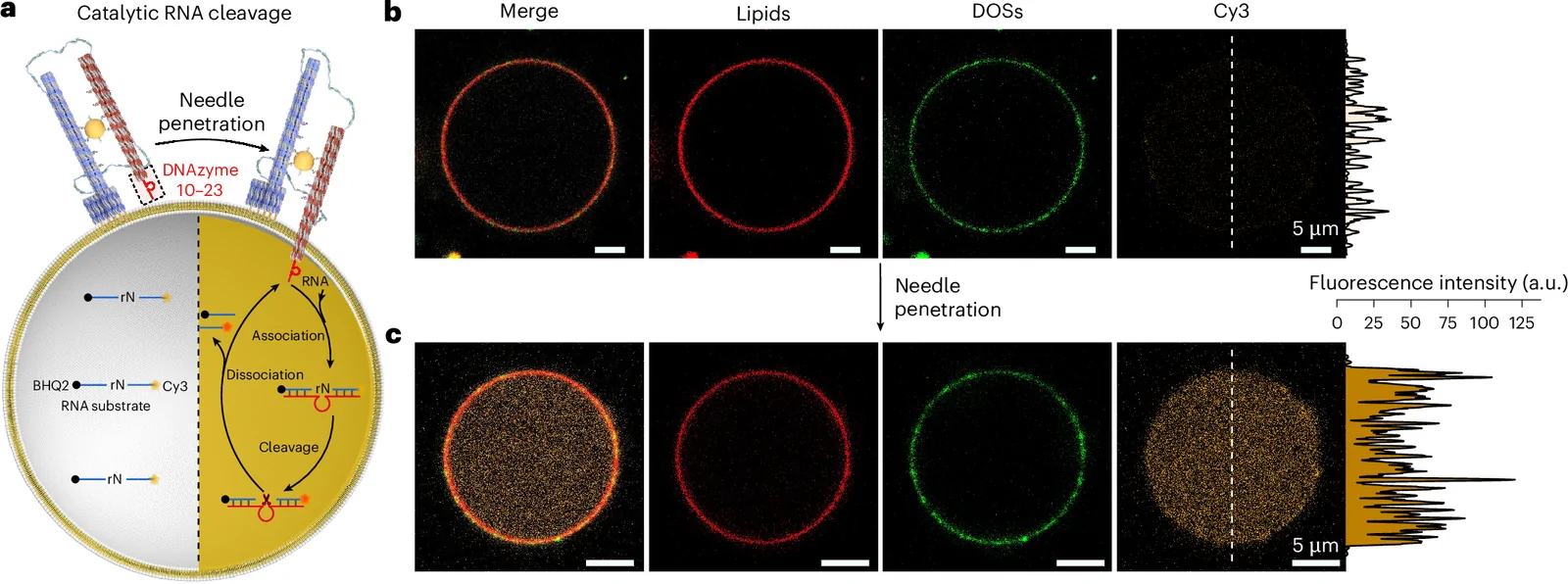
Nanosyringe-directed catalytic RNA cleavage in synthetic cells. **a**, Schematic of needle-guided translocation of 10–23 DNAzymes into a GUV, followed by catalytic RNA substrate cleavage. **b**, Confocal fluorescence images before needle penetration, showing minimal Cy3 signal within the GUV. **c**, Confocal fluorescence images after needle penetration, showing uniform Cy3 fluorescence throughout the lumen, indicating successful RNA cleavage. Fluorescence intensity profiles along the equatorial planes (Cy3 channel) are shown alongside **b** and **c**. Source data

To further demonstrate its versatility, we use the DOS platform to achieve catalytic RNA cleavage in GUVs by needle-guided transport of DNAzymes. We select the 10–23 DNAzyme, a biologically relevant RNA-cleaving enzyme known for its programmability and robust catalytic activity in gene silencing and RNA-targeting therapeutics^44,45^. This DNAzyme features two flanking arms (red) that specifically hybridize to complementary regions on the target RNA, aligning the substrate at its catalytic core (Fig. 6a). The 10–23 DNAzymes are tethered to the tip of the DOS needle. The target RNA (blue), containing a defined cleavage site, is labelled with a Cy3 fluorophore and a BHQ2 quencher at opposite termini. When pre-encapsulated within GUVs, the proximity of fluorophore and quencher results in minimal fluorescence due to Förster resonance energy transfer-based quenching (Fig. 6b and Supplementary Fig. 31). Upon the addition of DNA fuel strands, the DOS needle mechanically guides the DNAzymes across the membrane into the GUV lumen, where the RNA substrates reside. In the presence of divalent Mg^2+^ ions that diffuse through the needle’s central channels, the DNAzymes catalyse phosphodiester bond hydrolysis at the cleavage site, generating two RNA fragments. This physical separation of Cy3 and BHQ2 leads to a uniform fluorescence signal throughout the GUV lumen, indicating successful catalytic RNA cleavage (Fig. 6c and Supplementary Fig. 31). Together with time-resolved monitoring (Supplementary Fig. 32), these results demonstrate that DOSs can translocate functional nucleic acid catalysts across lipid membranes and reconstitute catalytic RNA processing reactions within a compartmentalized biochemical environment. In addition to tethered transport, we further demonstrate cargo release within GUVs. Specifically, cargo molecules initially tethered to the DOS needles can be released into the lumen via toehold-mediated strand displacement, thereby redistributing them from the membrane into the GUV interior (Supplementary Figs. 33–35). This expands the functional scope of DOS-enabled membrane translocation.

## Conclusion

We have demonstrated the DOS as a mechanically actuated and modular nanodevice that enables programmable membrane translocation. By integrating spatial precision through guided, site-specific transport and temporal precision via fuel-triggered actuation, the DOS allows on-demand control of biochemical reactions within cell-sized confinements. Furthermore, the DOS can function both as a transmembrane pore for passive diffusion of small molecules and as a mechanical needle for DNA fuel-triggered transport of large biomolecules. The membrane-proximal delivery of reactants enabled by the DOS establishes a surface-confined reaction environment at the membrane interface. Such confinement may lead to distinct reaction behaviour compared with bulk conditions^46^. This highlights the potential of the DOS as a platform for regulating biochemical processes under membrane-associated confinement in synthetic cell-like systems. It is noteworthy that the DOSs share conceptual similarities with microneedles used in ICSI, albeit with distinct characteristics. In ICSI, sperm injection requires manual micromanipulation by a skilled embryologist, making the process highly labour intensive. By contrast, the DOS, specifically anchored to the vesicle membrane, operates as a localized nanodevice directly at the delivery site. Cargo such as DNA, RNA or other bioentities can be tethered to the needle tip in a modular fashion and mechanically navigated across membranes via fuel-triggered actuation. This signal-responsive, self-administered mechanism eliminates manual intervention, advancing precision, efficiency and scalability in molecular delivery^47^. Notably, the current design relies on DNA-mediated cargo tethering, making it most readily applicable to biomolecules that can be conjugated to DNA. Future implementations using alternative tethering chemistries could further expand the scope of cargo delivery.

These distinctive attributes position the DOS as a platform that bridges the gap between nanoscale engineering and biological function. It exemplifies how precision nanotechnology can redefine approaches to membrane engineering, molecular transport and confined biochemical reactions. By advancing both fundamental insights and practical innovations, the DOS highlights the broader potential of DNA-based nanodevices in synthetic biology, cellular engineering, molecular diagnostics and therapeutic delivery, further expanding the scope and impact of DNA nanotechnology across diverse scientific fields^15,48,49^.

## Methods

### Materials

Scaffold DNA strands (p8064) were purchased from Tilibit Nanosystems. All DNA strands for the DNA origami were ordered from Sigma-Aldrich and stored at −20 °C. The detailed sequences are presented in Supplementary Tables 1–5. Lipids were purchased from Avanti Polar Lipids. The RNA substrates were purchased from Biomers. HiScribe T7 High Yield RNA Synthesis Kit was purchased from New England Biolabs. DFHBI was purchased from Biomol. Other chemicals were purchased from Sigma-Aldrich. Nuclease-free water was used throughout the RNA transcription procedures. Unless otherwise specified, all experiments were performed using ultrapure water (18.2 MΩ cm). Carbon-coated TEM grids were purchased from Ted Pella. Confocal imaging chambers were obtained from ibidi GmbH.

### Preparation and purification of DNA origami

The DOSs were designed using caDNAno software. To assemble the DNA origami, staple strands (Supplementary Tables 1–3) were mixed with scaffold strands at a molar ratio of 10:1 in TE-MgCl_2_ buffer (10 mM Tris, 1 mM EDTA and 16 mM MgCl_2_, pH 7.8). The mixture was then annealed in a polymerase chain reaction thermocycler using the following protocol: 85 °C for 5 min; 65 °C for 15 min; ramped from 60 °C to 40 °C at 45 min per degree Celsius; and from 40 °C to 25 °C at 25 min per degree Celsius; and stored at 20 °C. For the modified DOS variant with blocked channels, nine DNA strands were employed as mini-scaffolds (Supplementary Tables 4 and 5), and the MgCl_2_ concentration was raised to 28 mM to facilitate assembly. DNA origami structures were purified by 0.8% agarose gel electrophoresis in 0.5× TBE buffer with 11 mM MgCl_2_ at 80 V for 3 h under ice cooling. After incubation with 1× SYBR Gold for 30 min, the target band was excised and recovered by squeezing between two glass slides. The concentration of purified DNA origami was determined by ultraviolet–visible absorption spectroscopy and stored at 4 °C.

### Assembly and purification of DOSs

AuNPs were uniformly functionalized with thiol-modified DNA using a modified instant dehydration in butanol (INDEBT)-based method. Before use, colloidal AuNP solution (10 nm) was concentrated tenfold by centrifugation using a 100-kDa centrifugal filter for 10 min. Thiol-modified DNA (100 μM; Supplementary Table 3) was freshly mixed with Tris(2-carboxyethyl)phosphine (200 mM, pH 7.0) at a 1:5,000 ratio and incubated at 25 °C for 10 min. The activated DNA was then added to the concentrated AuNPs at a molar ratio of 500:1 in a final volume of 100 μl. This solution was immediately mixed with 900 μl of *n*-butanol by vortexing. Then, 200 μl of 0.5× TBE buffer (Tris 44.5 mM, EDTA 1 mM and boric acid 44.5 mM, pH 8.0) was added, followed by another vortex mixing. DNA-functionalized AuNPs were collected by brief centrifugation at 2,000*g* to facilitate phase separation, and purified by five cycles of centrifugation at 8,000*g* for 10 min using a 100-kDa filter, with redispersion in water to remove excess DNA. The DNA-functionalized AuNPs were then mixed with purified DNA origami at a molar ratio of 5:1 and annealed in a thermocycler from 35 °C to 25 °C at a ramp rate of 120 min per degree Celsius. After annealing, the samples were purified by 0.8% agarose gel electrophoresis in 0.5× TBE buffer with 11 mM MgCl_2_ at 80 V for 3 h under ice cooling, and the target band was excised and recovered by squeezing between two glass slides. The obtained DOSs were stored at 4 °C. For TEM imaging, the grids were stained for 10 s using a 2% aqueous uranyl formate solution containing 25 mM NaOH.

### Preparation of SUVs

SUVs were prepared using the thin-film hydration, sonication and extrusion method. Lipid stock solutions in chloroform were aliquoted into glass vials under a fume hood using a cleaned Hamilton syringe. The chloroform was evaporated under a nitrogen stream, and the lipid films were further dried under vacuum at ~2 mbar for at least 8 h. The dried lipids were then hydrated with Milli-Q water to a concentration of 1 mg ml^−1^, followed by sonication in an ice bath for 1 h at 10% amplitude with a pulse cycle of 10 s on and 5 s off using a probe sonifier (Branson, 40 kHz). The resulting suspension was extruded through polycarbonate membranes from 100 nm down to 50 nm pore size with 11 passes to obtain monodisperse SUVs.

### Preparation of GUVs

GUVs were prepared using the Vesicle Prep Pro device (Nanion Technologies). A 2 mg ml^−1^ lipid stock solution was prepared by mixing chloroform solutions of DOPC and Atto655-DOPE at a molar ratio of 99.95:0.05. Then, 30 µl of the lipid solution was deposited onto the conductive side of a cleaned ITO-coated glass slide. After drying at room temperature for 20 min to remove residual solvent, another cleaned ITO slide was assembled to form an electroformation chamber with both conductive sides facing inward. Finally, 200 µl of 220 mM sucrose solution was added as the hydration buffer, followed by the application of an alternating current electric field (3 V, 5 Hz) at 37 °C for 2 h. For GUVs designed to encapsulate specific cargo, the sucrose solution was supplemented with the desired components instead of using pure sucrose. Specifically, sucrose containing 100 nM of two hairpin oligonucleotides (Cy3-labelled H1 and unlabelled H2; Supplementary Table 3) was used for GUV encapsulation in the HCR experiment, while sucrose containing 100 nM RNA substrate (Supplementary Table 3) was used for the RNA cleavage experiment.

GUVs used for RNA transcription were prepared using the emulsion transfer method. In brief, 80 µl of chloroform-solubilized DOPC (25 mg ml^−1^) containing Atto655-DOPE was dried under a nitrogen stream to form a lipid film, followed by overnight vacuum drying. 650 µl mineral oil predried with activated molecular sieves was added to obtain a 3 mg ml^−1^ lipid-in-oil solution by vortexing and sonication. For the exterior space, 200 µl lipid-in-oil solution was gently layered over 200 µl of outer buffer (40 mM HEPES, 125 mM KCl, 120 mM NaCl and 12 mM MgCl_2_) in a 2-ml tube and left undisturbed for 30 min. For the interior space, 7 µl of solution containing 220 mM sucrose, 40 mM HEPES, 125 mM KCl, 12 mM MgCl_2_, 62.5 µM DFHBI, 3 mM of each NTP, 5 mM dithiothreitol, 1 mM spermidine, 200 nM DNA template and T7 RNA polymerase (diluted 1:10 from the HiScribe T7 kit) was prepared. The dithiothreitol, NTPs and polymerase mix were used directly from the kit. This solution was mixed into 450 µl lipid-in-oil solution. The emulsion was then transferred onto the prepared outer phase and centrifuged at 4,500*g* for 10 min at 5 °C. Following centrifugation, the oil phase was carefully removed, and the aqueous phase was gently pipetted to redisperse the GUVs, yielding the final suspension.

### Single-channel current recording

Electrophysiological current measurements were performed using the Orbit Mini system (Nanion Technologies) equipped with a four-well recording chip (Ionera) containing 100-µm cavities. The electrolyte solution consisted of 1 M KCl and 10 mM HEPES (pH 7.6). Following bilayer formation composed of 1,2-diphytanoyl-*sn*-glycero-3-phosphocholine (DPhPC), a mixture of DOSs and 0.5% poly(ethylene glycol) octyl ether (2:1, in the same buffer) was added to the chamber. After 30 min incubation with DOS to allow membrane binding, blocking and removal strands were sequentially introduced as fuel. Current recording was initiated upon addition of the first pair (B1/R3; *t* = 0 min), and the second pair (B2/R4) was added at *t* = 15 min. For natural transmembrane proteins, including OmpF and aerolysin, current recording was initiated upon protein addition. Pore formation was identified by the appearance of stepwise current signals recorded at a sampling rate of 20 kHz.

### Confocal fluorescence microscopy

Confocal imaging was performed using a Zeiss LSM 980 laser scanning microscope equipped with a 63× oil immersion objective. DOSs were mixed with 10 μl of GUV stock to a final concentration of 1 nM in 90 μl of imaging buffer (0.5× TBE, 11 mM MgCl_2_, 65 mM NaCl, pH 7.4, ~220 mOsm kg⁻^1^) and incubated at room temperature for 2 h. For the RNA transcription experiments, GUVs in outer solution was directly used without the addition of imaging buffer. A 200-μl imaging chamber was pretreated with 100 μl of 2 mg ml^−^^1^ bovine serum albumin (BSA). After 3 min, the BSA solution was removed and the DOS–GUV mixture was introduced. After 30-min incubation for membrane attachment, to actuate the sliding of DOSs on the membrane, 1 μl of blocking strands (B1–B4) and removal strands (R1–R4) at 200 μM were sequentially added, with each sliding step incubated at room temperature for 30 min. For small-molecule transport, molecules (for example, SRB) were added to the external solution at a final concentration of 1 μM following the second 30-min sliding-step incubation, and confocal images were acquired 12 h after addition. For FRAP experiments, a defined region of interest on GUV membranes containing DOSs was photobleached, and fluorescence recovery was monitored over time. All DOS-directed reactions within GUVs were performed at room temperature (~25 °C). Statistical analysis of GUVs was performed using MATLAB following established analytical procedures^18^. Fluorescence intensity line profiles from confocal images in Figs. 4–6 were extracted and plotted using ImageJ.

### Statistics and reproducibility

No statistical method was used to predetermine sample size. No data were excluded from the analyses. The experiments were not randomized. The investigators were not blinded to allocation during experiments and outcome assessment.

## Online content

Any methods, additional references, Nature Portfolio reporting summaries, source data, extended data, supplementary information, acknowledgements, peer review information; details of author contributions and competing interests; and statements of data and code availability are available at https://doi.org/10.1038/s41565-026-02249-3.

## Supplementary information

Supplementary Information Supplementary Figs. 1–35, Tables 1–5 and References.

## Source data

Source Data Fig. 2 Statistical source data.

Source Data Fig. 3 Statistical source data.

Source Data Fig. 4 Statistical source data.

Source Data Fig. 5 Statistical source data.

Source Data Fig. 6 Statistical source data.

Source Data Extended Data Fig. 3 Statistical source data.

## Data Availability

All the data that support the findings of this study are available within the article and its Supplementary Information. Source data are provided with this paper.
