# Supplementary material for: A programmable DNA origami nanosyringe for directed membrane translocation
Source: Nat Nanotechnol. 2026 Aug 11;21(10):1459–66. doi: 10.1038/s41565-026-02249-3 (PMC13661252; doi:10.1038/s41565-026-02249-3)
Supplement: Supplementary file 1 [file 41565_2026_2249_MOESM1_ESM.pdf]

nature nanotechnology

Supplementary information https://doi.org/10.1038/s41565-026-02249-3

A programmable DNA origami nanosyringe for directed membrane translocation

In the format provided by the authors and unedited

Table of contents

Supplementary Figures…….……………………………………...………….………………2

Supplementary Tables………………………………………………….…………….…......29

References…………………………………………………………………………….....40

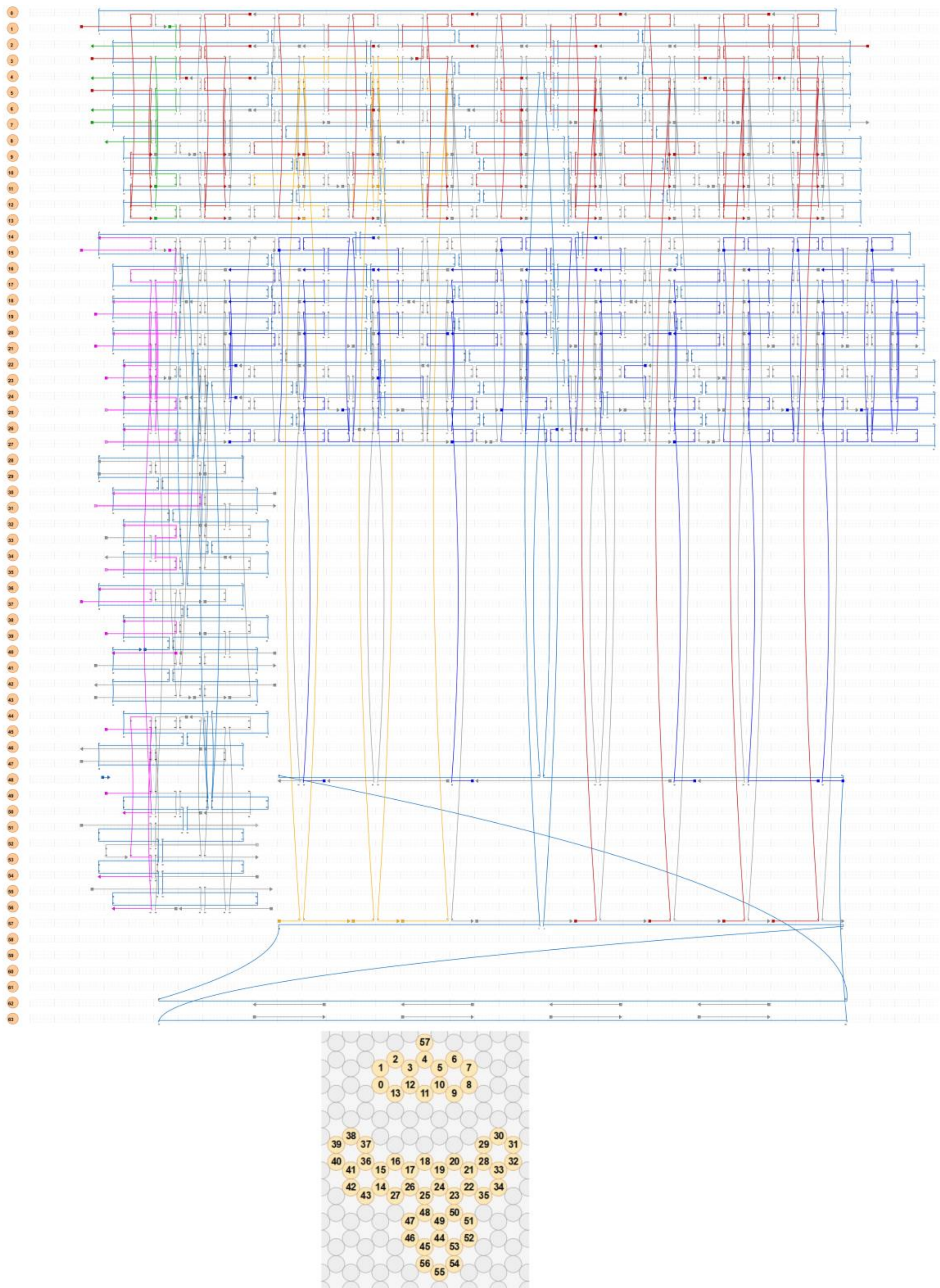

Figure S1. caDNAno design of the DNA origami in DOS. The cross-section of the structure is shown at the bottom of the strand diagram. Both were generated using caDNAno.

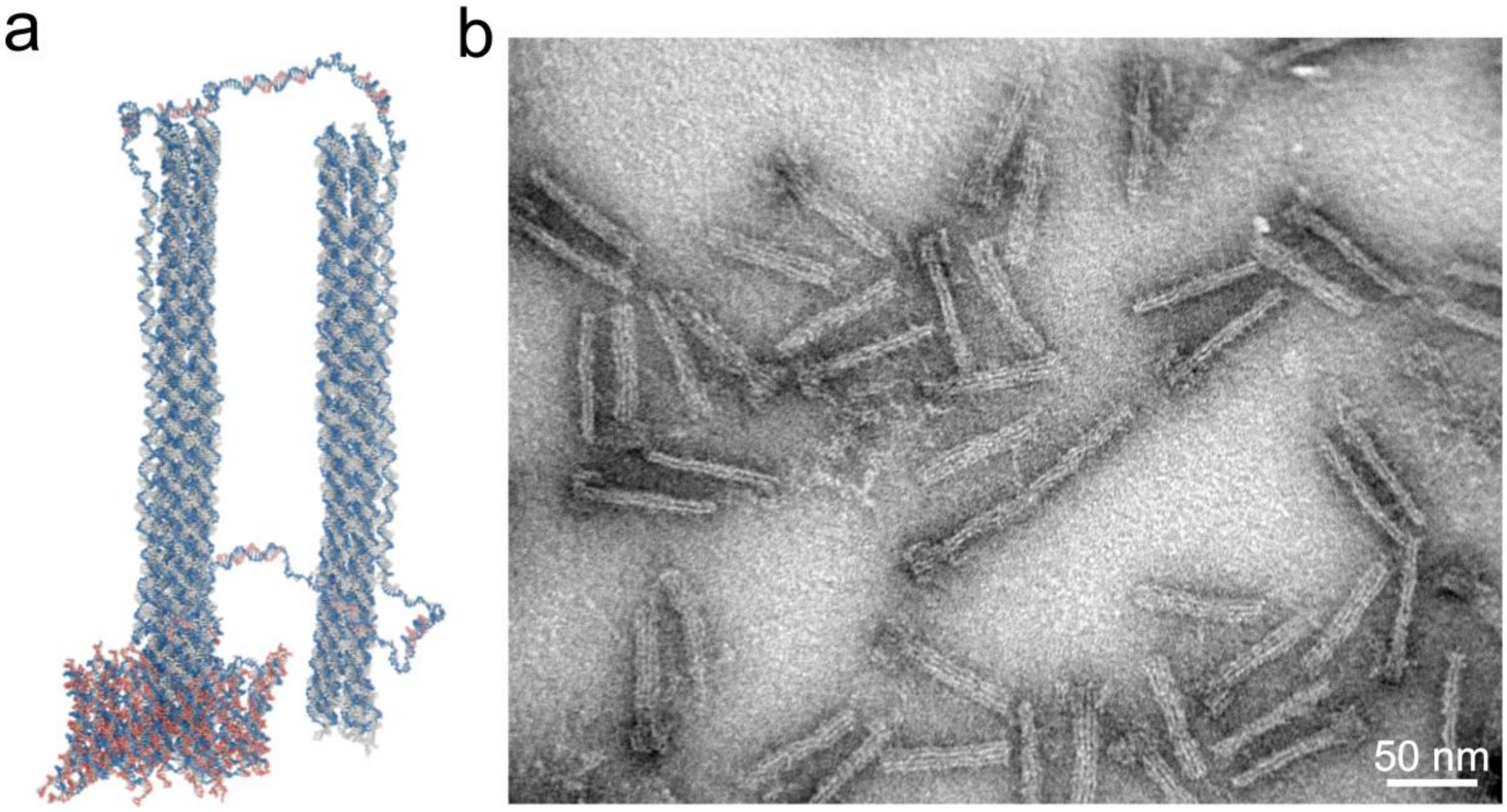

Figure S2. DNA origami component of the DOS. a, oxDNA simulation of the DNA origami. **b**, TEM image of the assembled DNA origami structures.

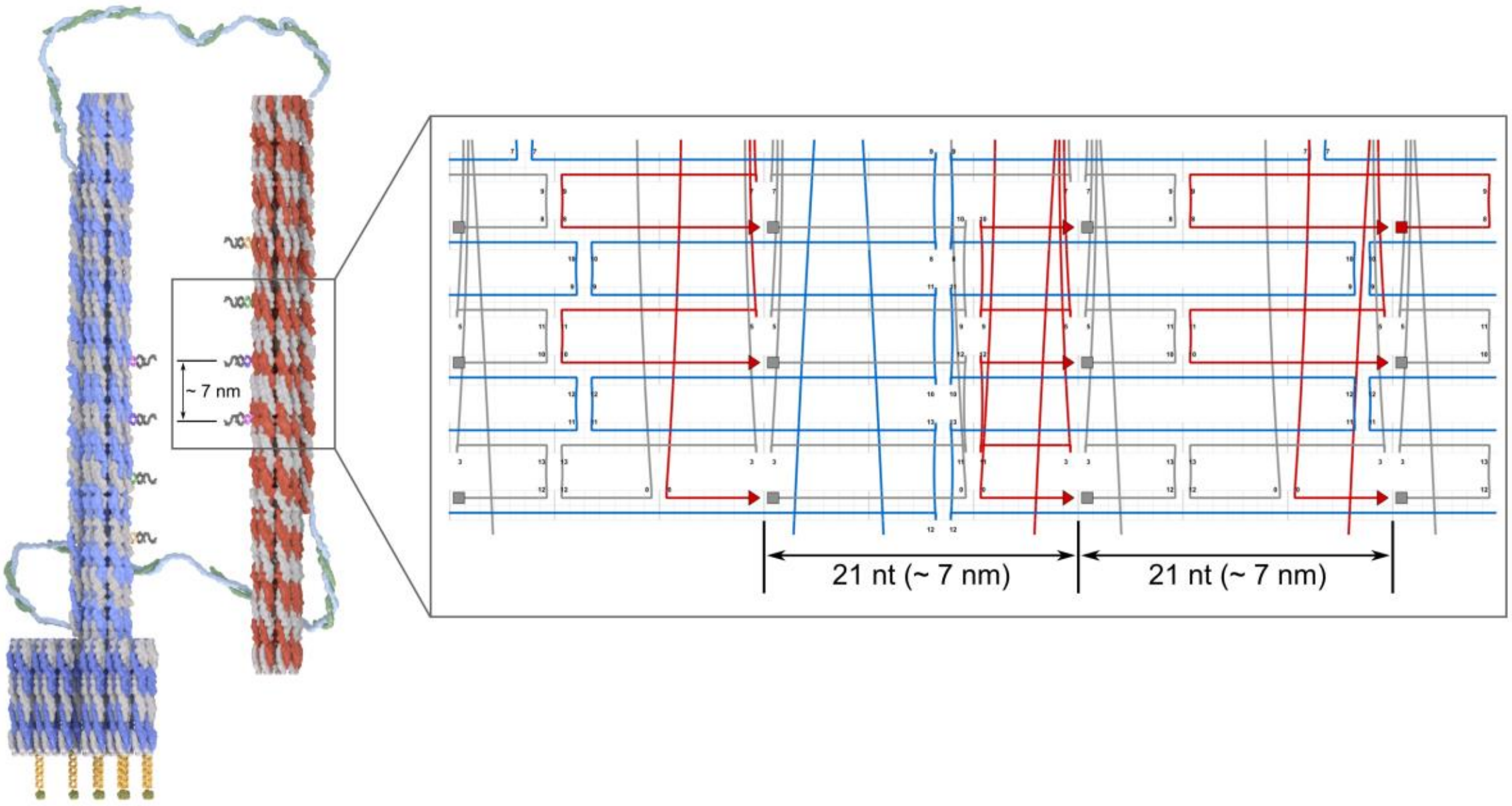

Figure S3. caDNAno design showing the staple arrangement of foothold regions. Considering crossover placement and the geometric constraints of DNA origami, a minimal spacing of 21 nt (two helical turns, ~7 nm) between adjacent footholds is required to maintain consistent orientation of the extended DNA strands on the same face of the origami.

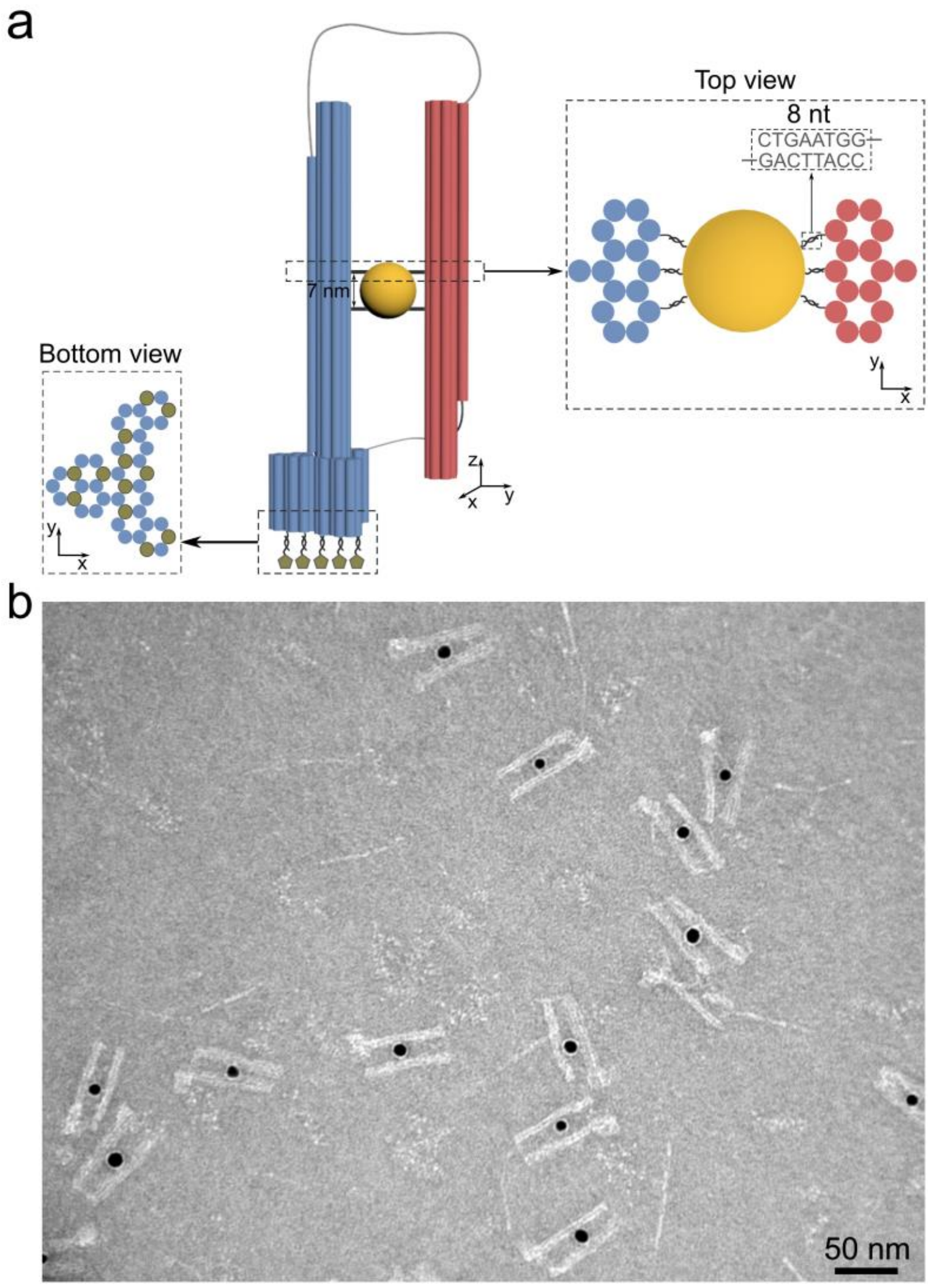

Figure S4. Structural characterization of the DOS. a, Schematic illustration of the DOS, highlighting AuNP linkage between the two origami bundles and the spatial distribution of cholesterol anchors for membrane binding. Foothold rows containing defined toehold segments are arranged along each DNA origami bundle at ~7 nm intervals. Each row comprises three identical binding sites. The foothold rows are arranged in reverse order on the two bundles, thereby introducing opposite polarities. The AuNP (~10 nm) binds between the two bundles by simultaneously engaging four foothold rows, two from each bundle, via complementary 8 nt foothold sequences. For membrane binding, twelve cholesterol-modified DNA strands are positioned on the underside of the DOS base, which consists of a ~14 nm multi-helix segment appended to bundle I. **b,** TEM image of assembled DOS structures.

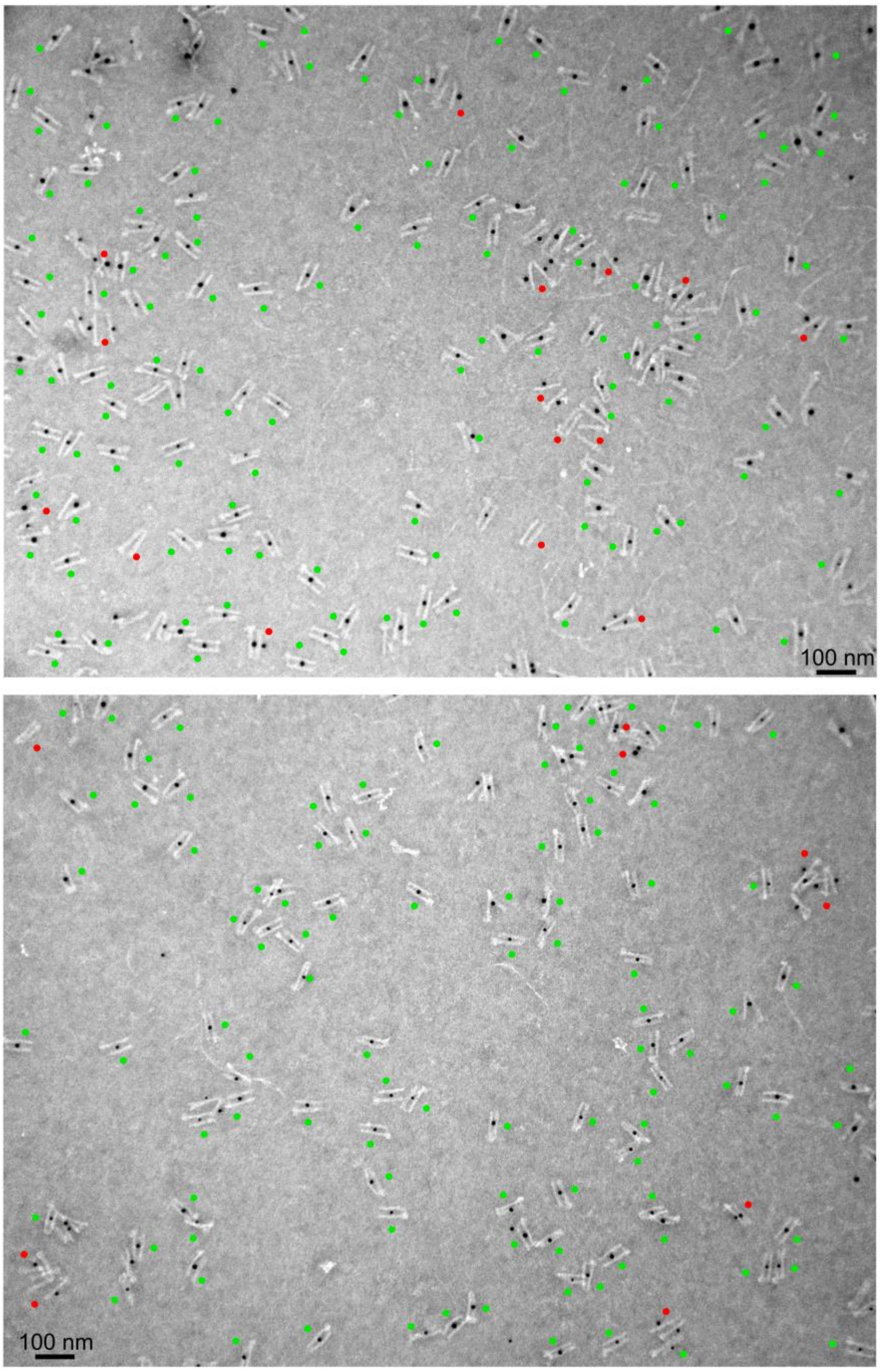

Figure S5. Quantification of DOS assembly efficiency from TEM images after purifications. Green and red markers indicate correctly assembled and misassembled DOS structures, respectively. A total of 214 correctly assembled and 22 misassembled structures were identified, corresponding to an assembly efficiency of ~91%.

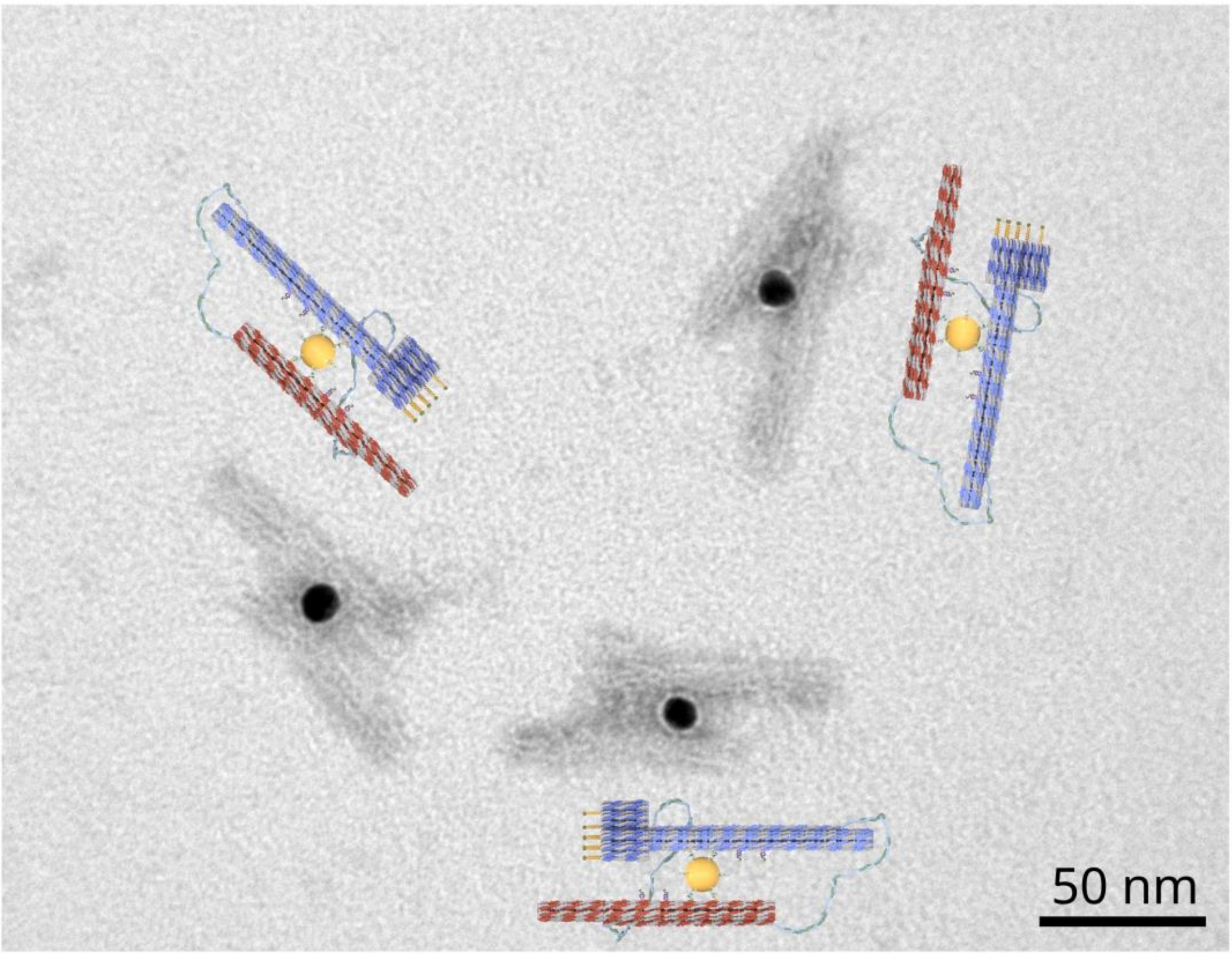

Figure S6. TEM image of DOSs after two-step sliding. Representative TEM image of DOSs after two sequential sliding steps, corresponding to a total displacement of 28 nm.

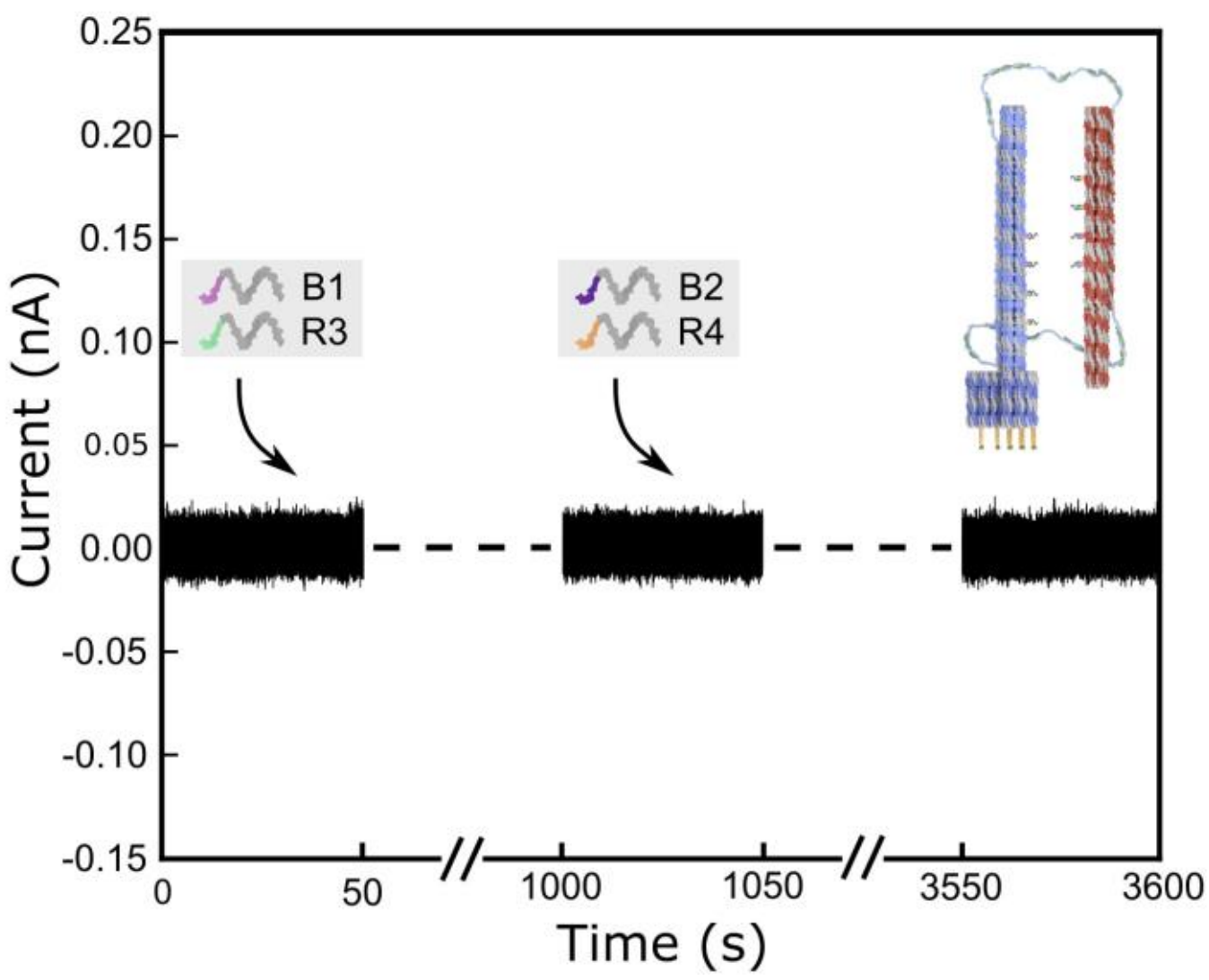

Figure S7. Ionic current recording of DNA origami without AuNP crosslinker. No detectable current signal is observed. The measurement was conducted in 1 M KCl and 10 mM HEPES (pH 7.6) at an applied membrane potential of 50 mV.

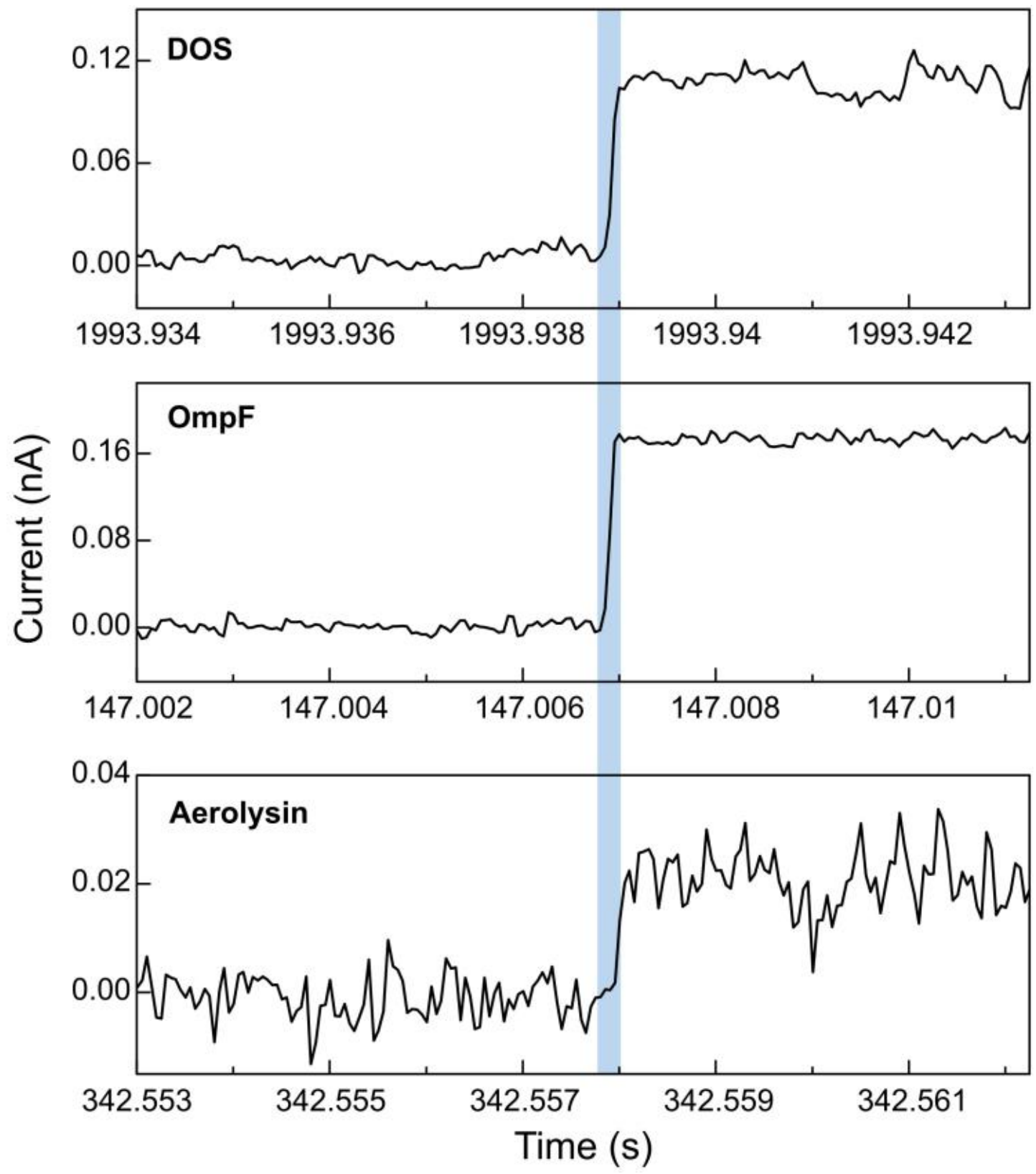

Figure S8. Comparison of current rising times between sliding-induced DOS perforation and natural transmembrane proteins, including outer membrane protein F (OmpF) and aerolysin. For comparison, segments of the recordings are extracted and aligned at the onset of the current step increase, resulting in different x-axis origins for each nanopore type. DOS, OmpF, and aerolysin exhibit comparable current step rising times, indicating similar temporal profiles of pore formation. The measurements were conducted in 1 M KCl and 10 mM HEPES (pH 7.6) at an applied membrane potential of 50 mV.

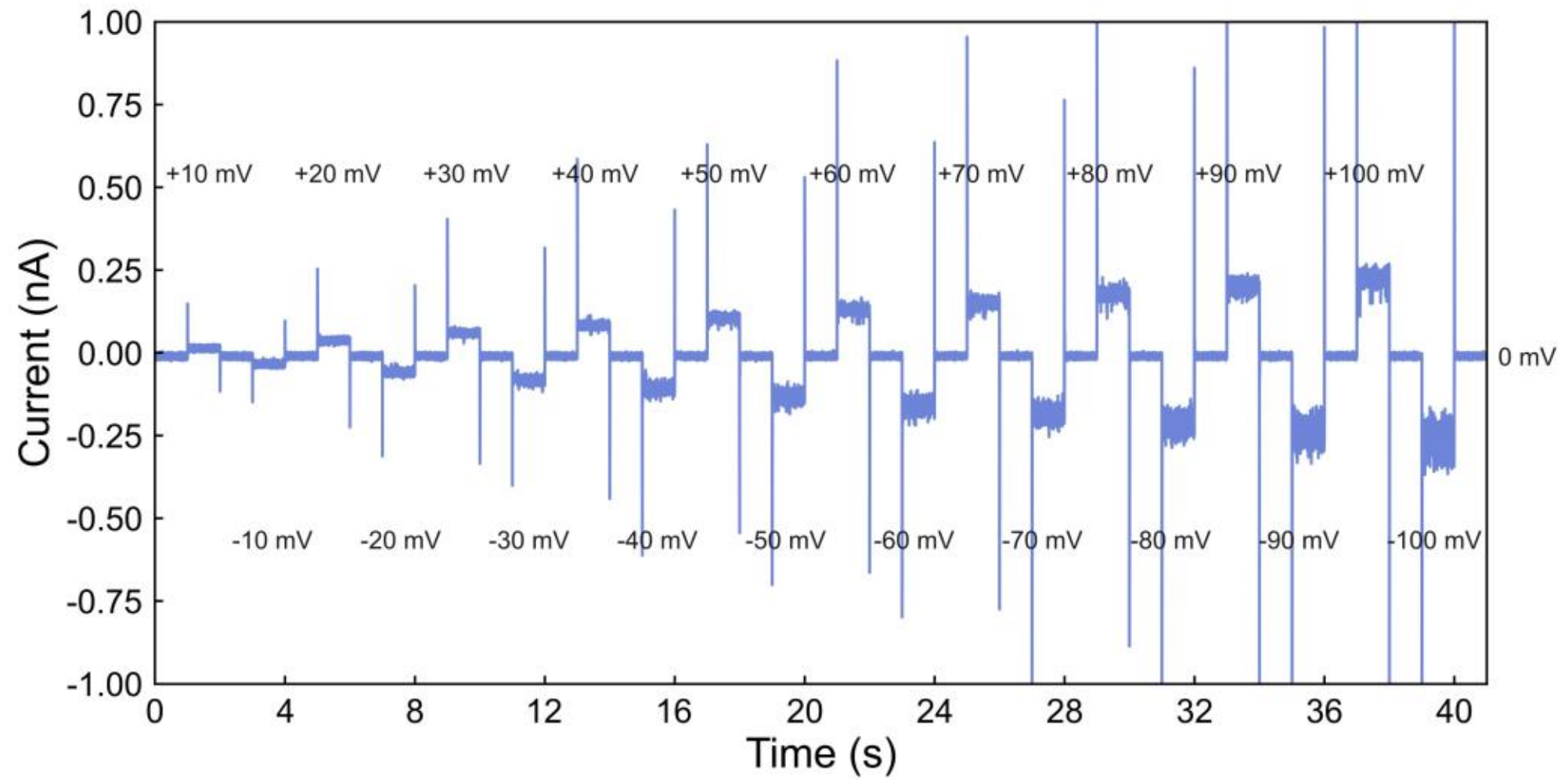

Figure S9. Current traces of a single DOS recorded by voltage stepping. The applied voltage was alternated in ±10 mV increments, starting at 0 mV and ending at -100 mV.

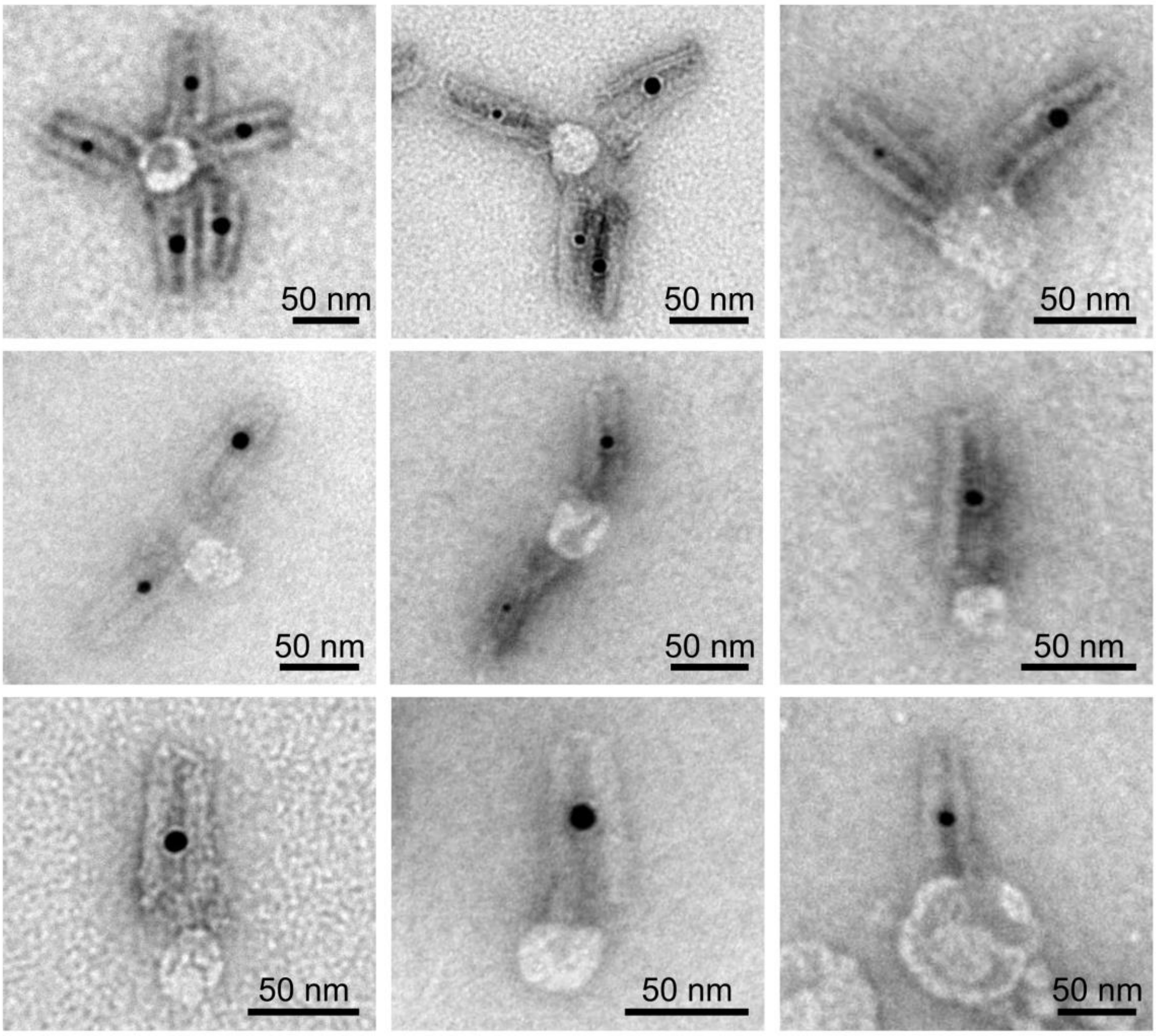

Figure S10. Representative TEM images of DOSs bound to SUVs. The DOSs are vertically oriented on the membrane surface, anchored via their cholesterol-modified bases.

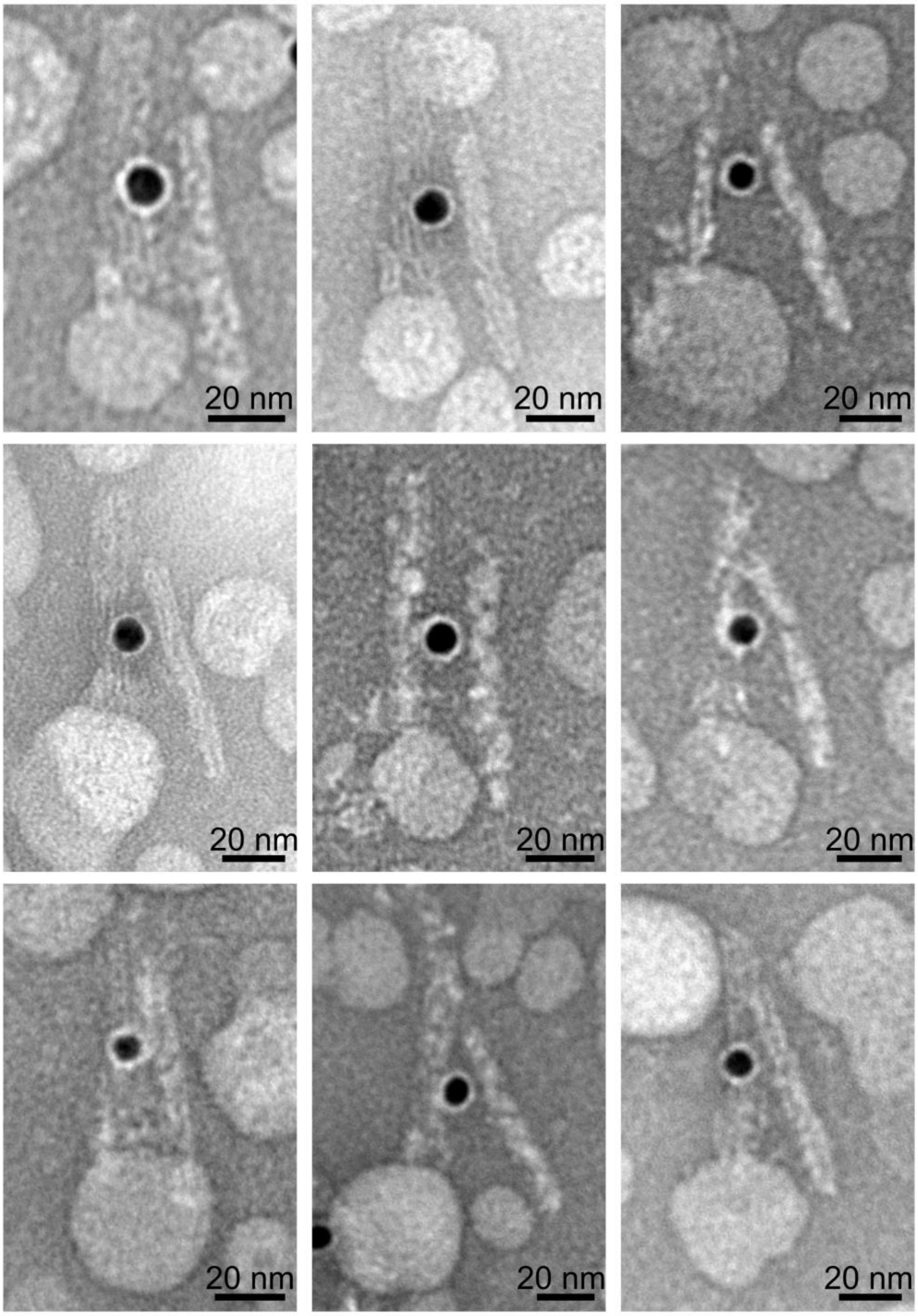

Figure S11. Representative TEM images of DOSs anchored to SUVs after needle sliding. The observed asymmetry between the two bundles reflects the relative movement of the needle following the sliding motion.

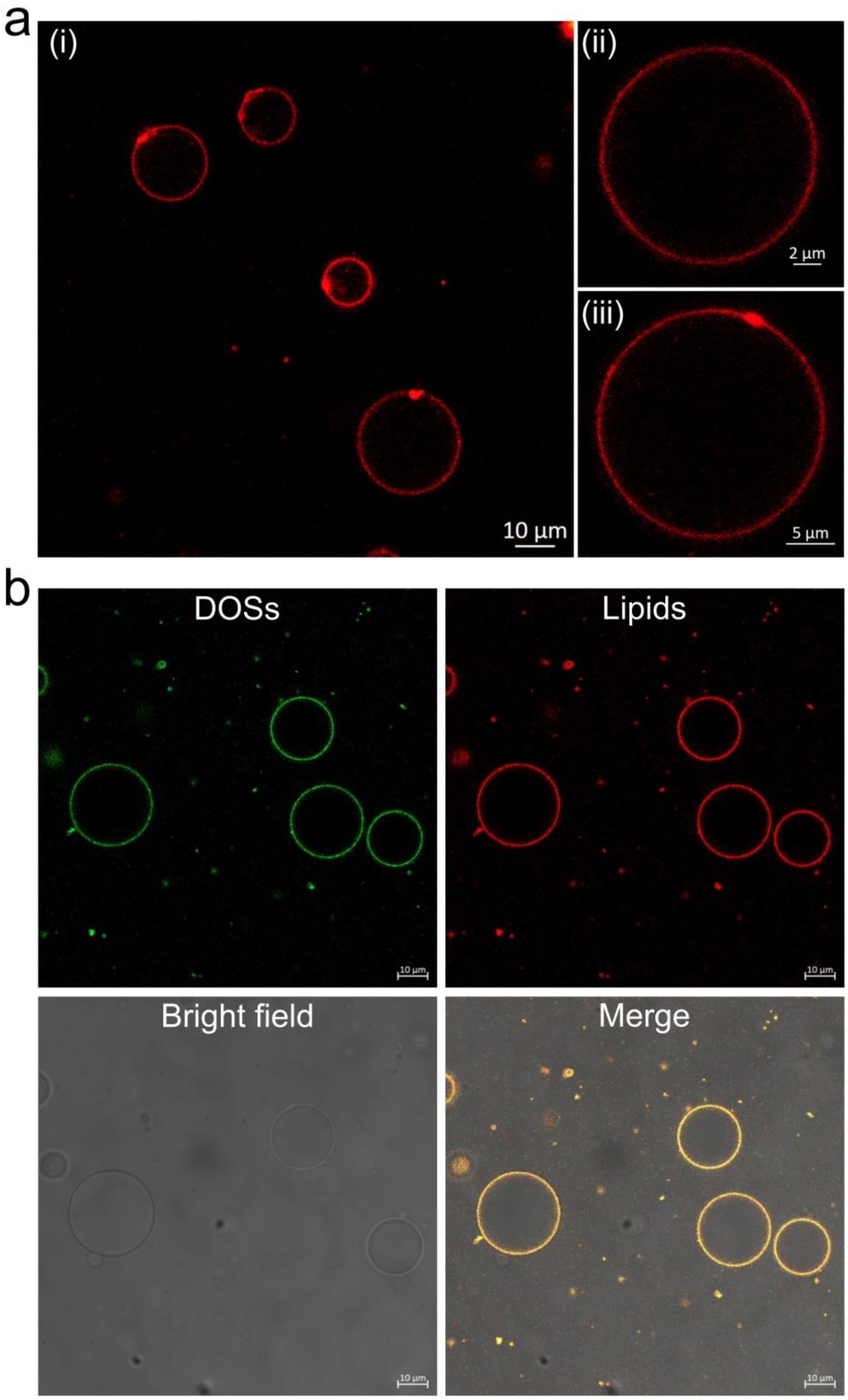

Figure S12. Confocal images of Guvs and DOS-bound GUVs. a, Confocal fluorescence images of Atto655-labeled GUVs. (i), Wide-field confocal image of multiple GUVs. (ii), Magnified view of a representative GUV showing a relative uniform lipid fluorescence distribution along the membrane. (iii), Magnified view of a representative GUV exhibiting localized fluorescence intensity variations, indicative of intrinsic membrane heterogeneity. **b,**

Colocalization of fluorescence signals from DOSs (green) and lipids (red) confirm successful membrane binding.

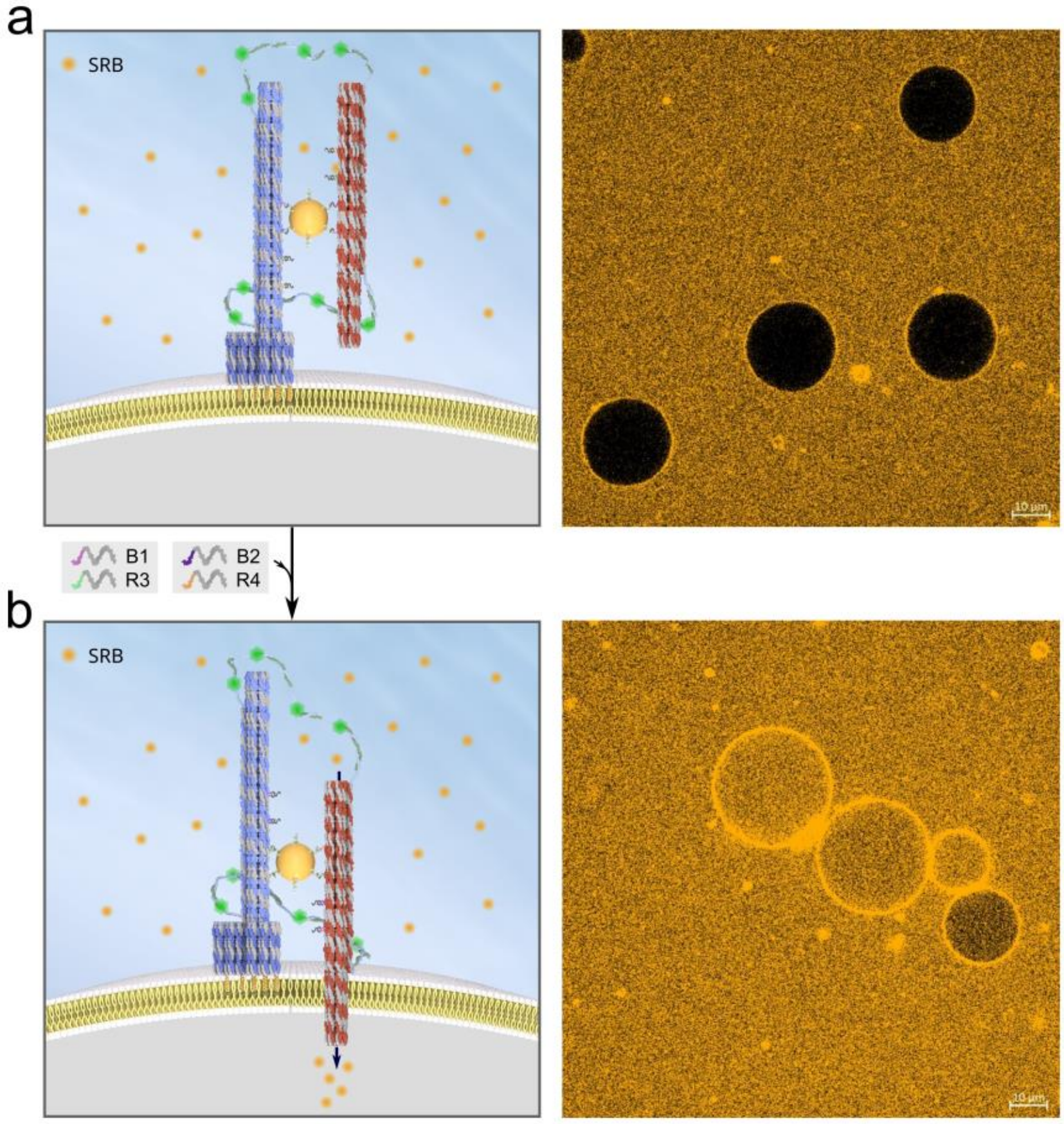

Figure S13. Confocal fluorescence images of DOS-bound GUVs incubated with SRB. a, Prior to needle penetration, no SRB fluorescence was detected inside the GUV lumen. **b**, Following activation, SRB signal appeared and distributed homogeneously within the lumen, confirming membrane perforation by DOSs.

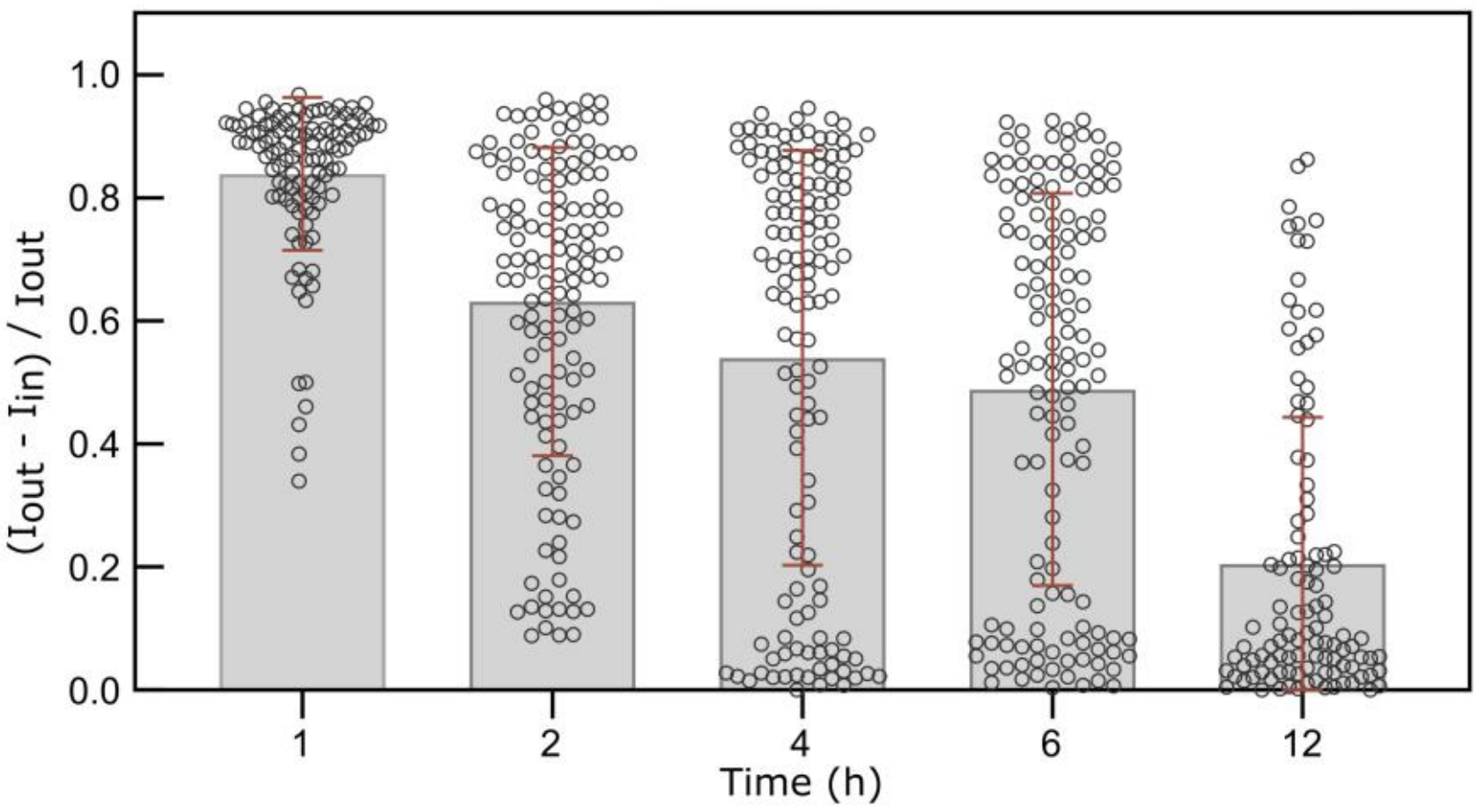

Figure S14. **Time-resolved confocal imaging of SRB transport**. Fluorescence intensity analysis shows a time-dependent decrease in $(I_{out} - I_{in})/I_{out}$ over 12 h, reflecting progressive

transport of SRB into the GUV lumen following membrane perforation.[1] Data represent mean ± s.d. from three independent experiments. The numbers of GUVs analyzed at 1, 2, 4, 6, and 12 h are 110, 135, 133, 137 and 108, respectively.

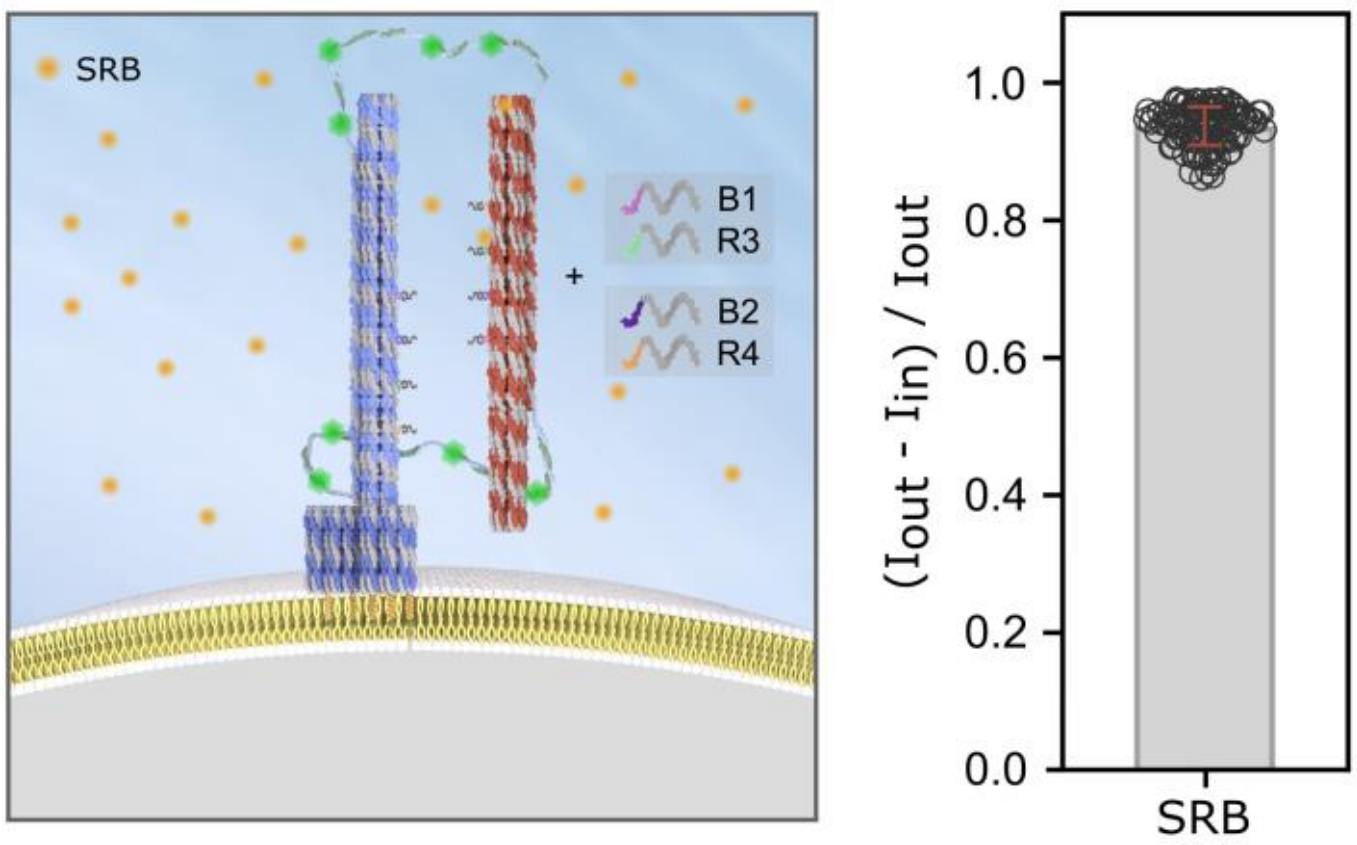

Figure S15. SRB influx assay using DNA origami without AuNP crosslinker. No detectable SRB influx is observed following addition of fuel strands.

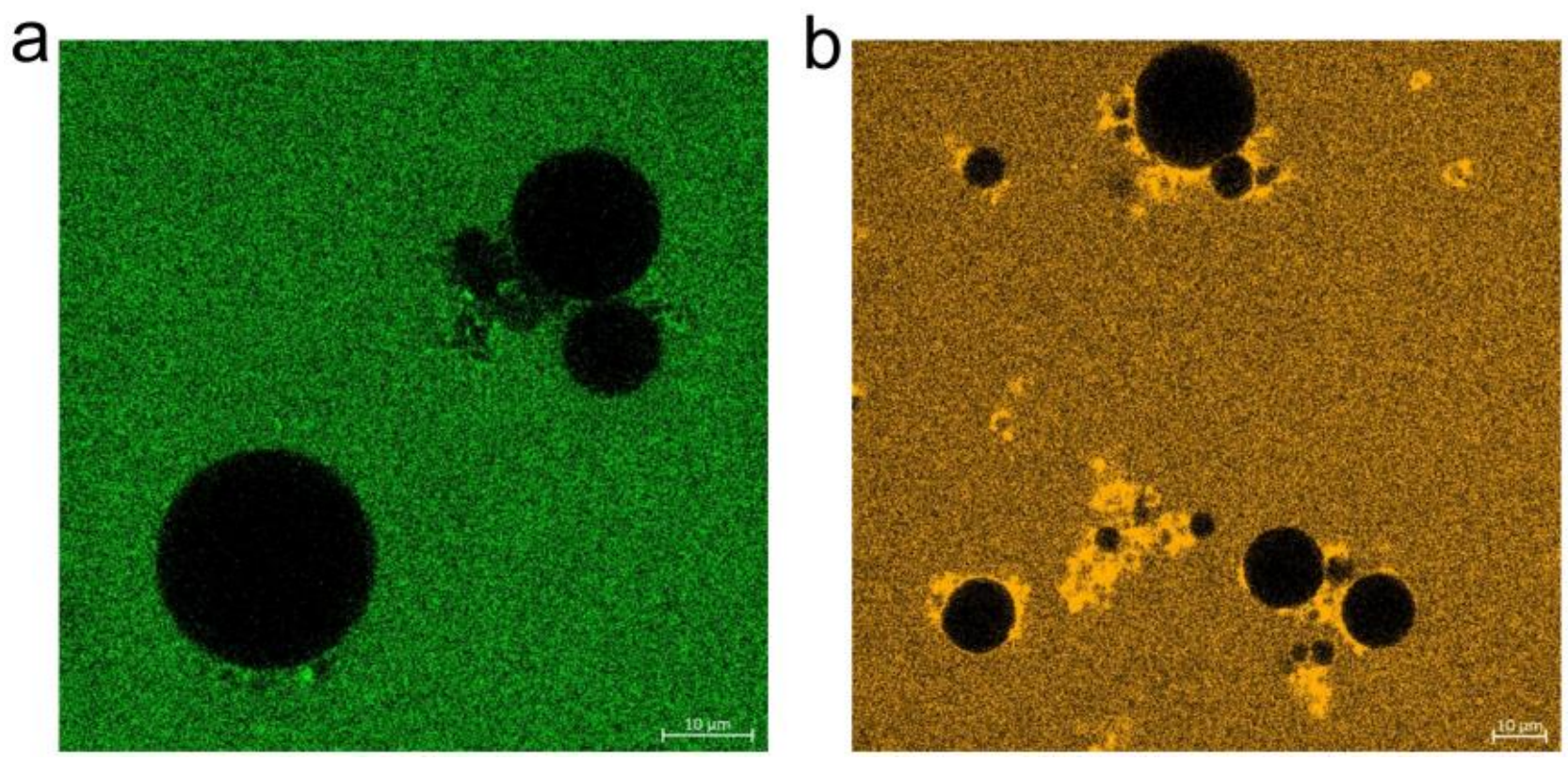

Figure S16. Confocal fluorescence images of DOS-bound GUVs with dyes. a, With CF. **b**, With 3 kDa Dextran-Cy3**.** In both cases, no detectable fluorescence signals were observed inside GUVs.

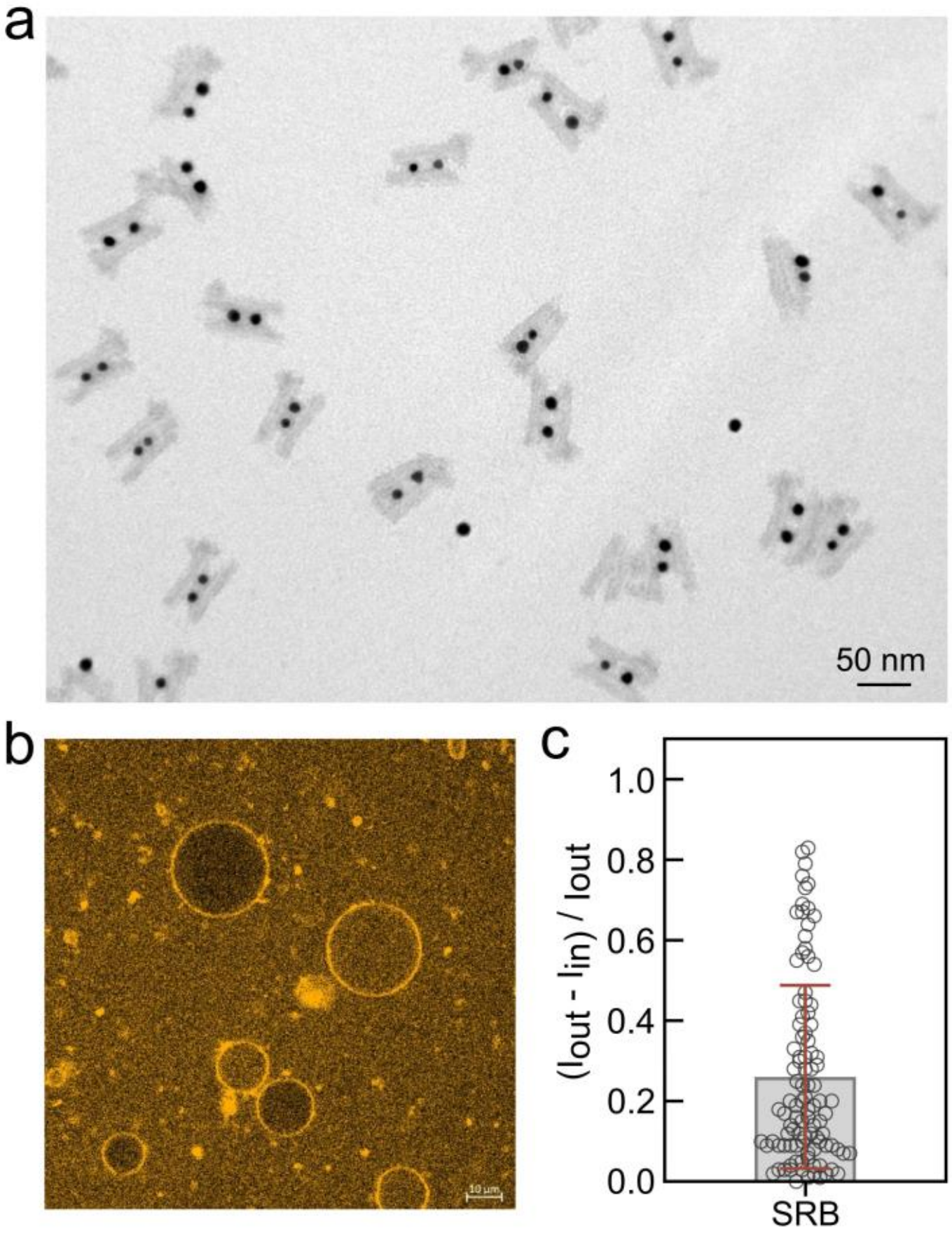

Figure S17. Membrane penetration by a DOS variant containing two AuNPs. a, Representative TEM image of assembled DOS structures with two AuNPs. **b**, Representative confocal fluorescence image of GUVs after needle penetration and incubation with SRB. **c**, Quantification of SRB influx following needle penetration. Data represent mean ± s.d. from three independent experiments (n = 101).

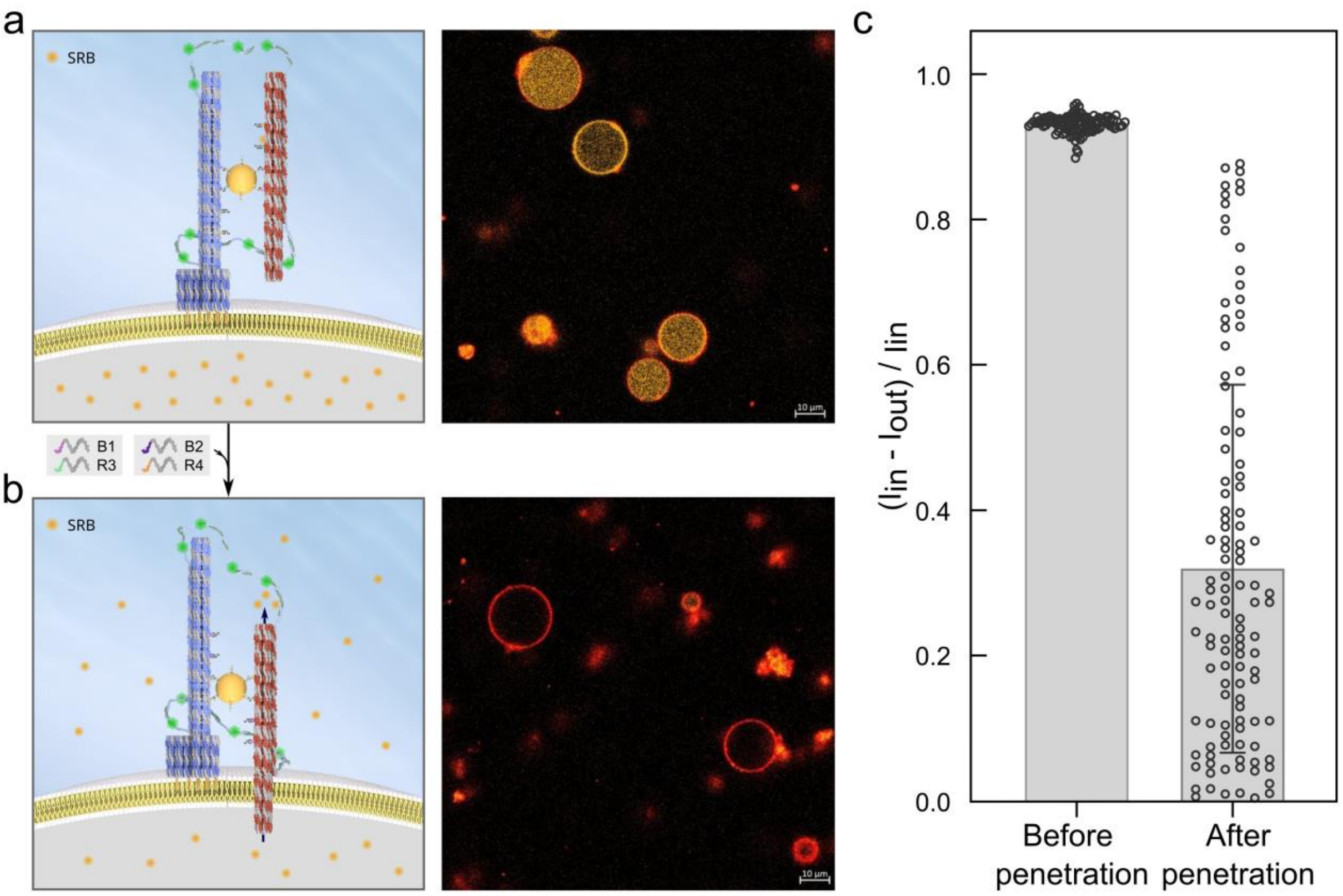

Figure S18. SRB efflux from GUVs following DOS penetration. a, Before needle penetration. b, After needle penetration. Lipid channel fluorescence was merged with the SRB signal in the confocal images to visualize GUVs post-efflux. **c**, Quantification of the normalized fluorescence intensity difference, efflux metric= $(I_{in}-I_{out})/I_{in}$ at equilibrium showed efficient SRB release from the GUV lumen after needle penetration. n = 108 (before) and 112 (after).

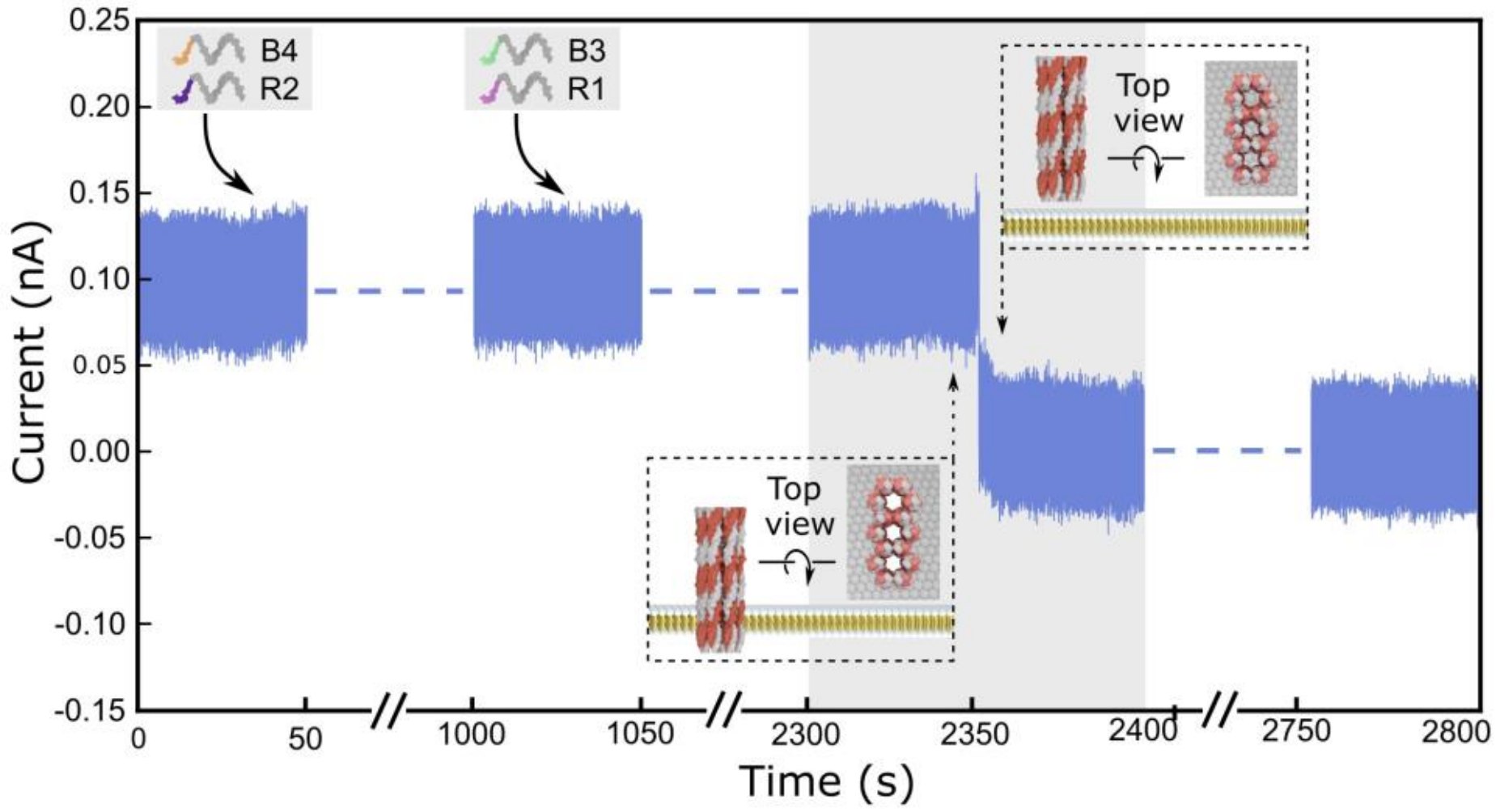

Figure S19. Ionic current recording of DOS needle retraction. Following insertion, a stable elevated current level is observed. After ~10 min to confirm signal stability, the first pair of DNA fuel strands for reversible sliding is added, with no significant change in current. Subsequent addition of the second pair of fuel strands induces a decrease in current toward the baseline level. The recovery occurs over a finite transition before stabilizing near the baseline. The measurement was conducted in 1 M KCl and 10 mM HEPES (pH 7.6) at an applied membrane potential of 50 mV.

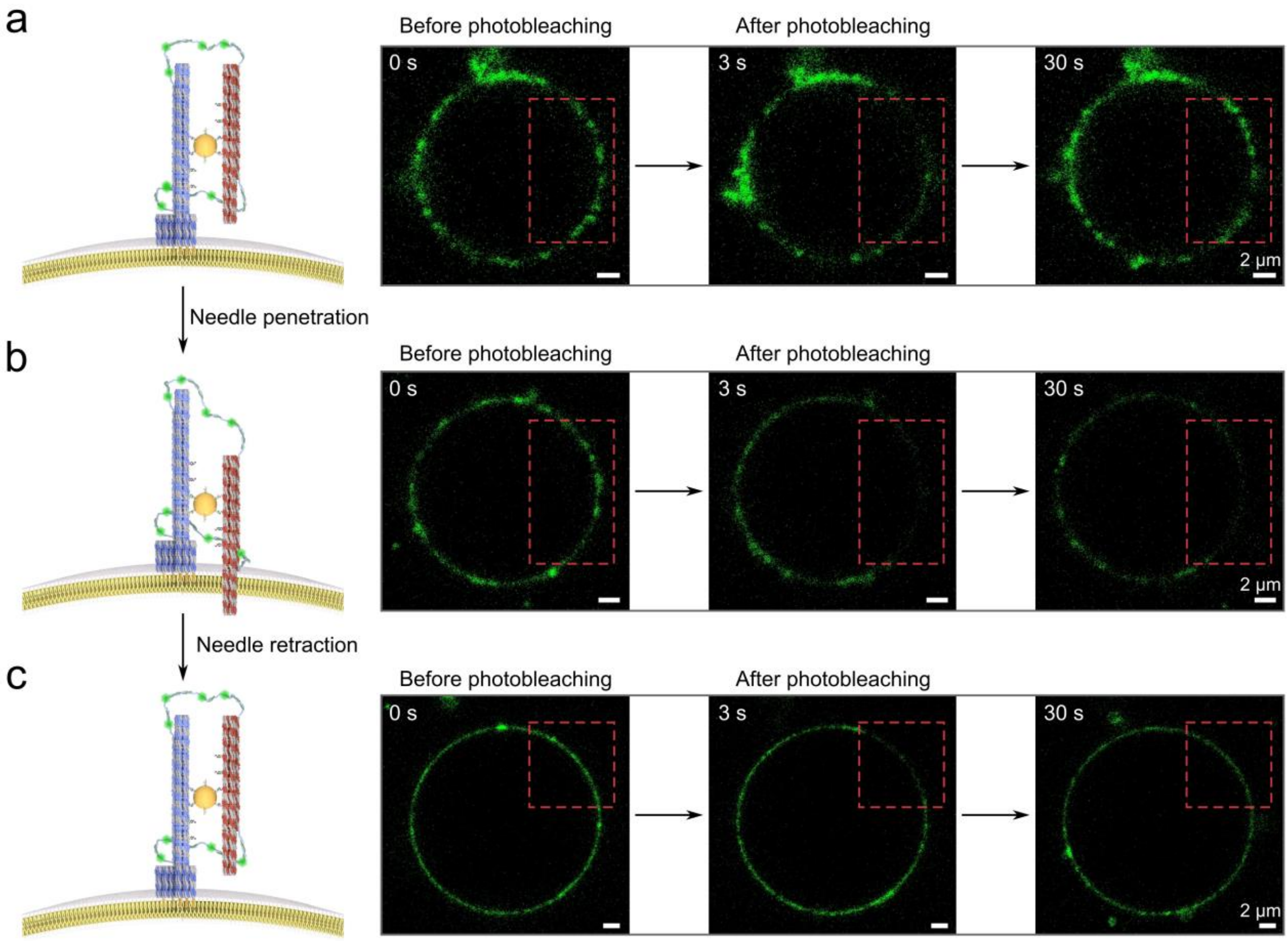

Figure S20. DOS needle penetration and retraction demonstrated by FRAP. Confocal fluorescence images of Atto488-labeled DOS-bound GUVs for FRAP experiments. **a**, Before needle penetration, showing rapid fluorescence recovery. **b**, Needle penetration, showing no fluorescence recovery. **c**, Needle retraction. Fluorescence was restored. The half-times of fluorescence recovery ($t_{1/2}$) for states (a) and (c) are 10.4 ± 0.6 s and 15.6 ± 0.8 s, respectively.

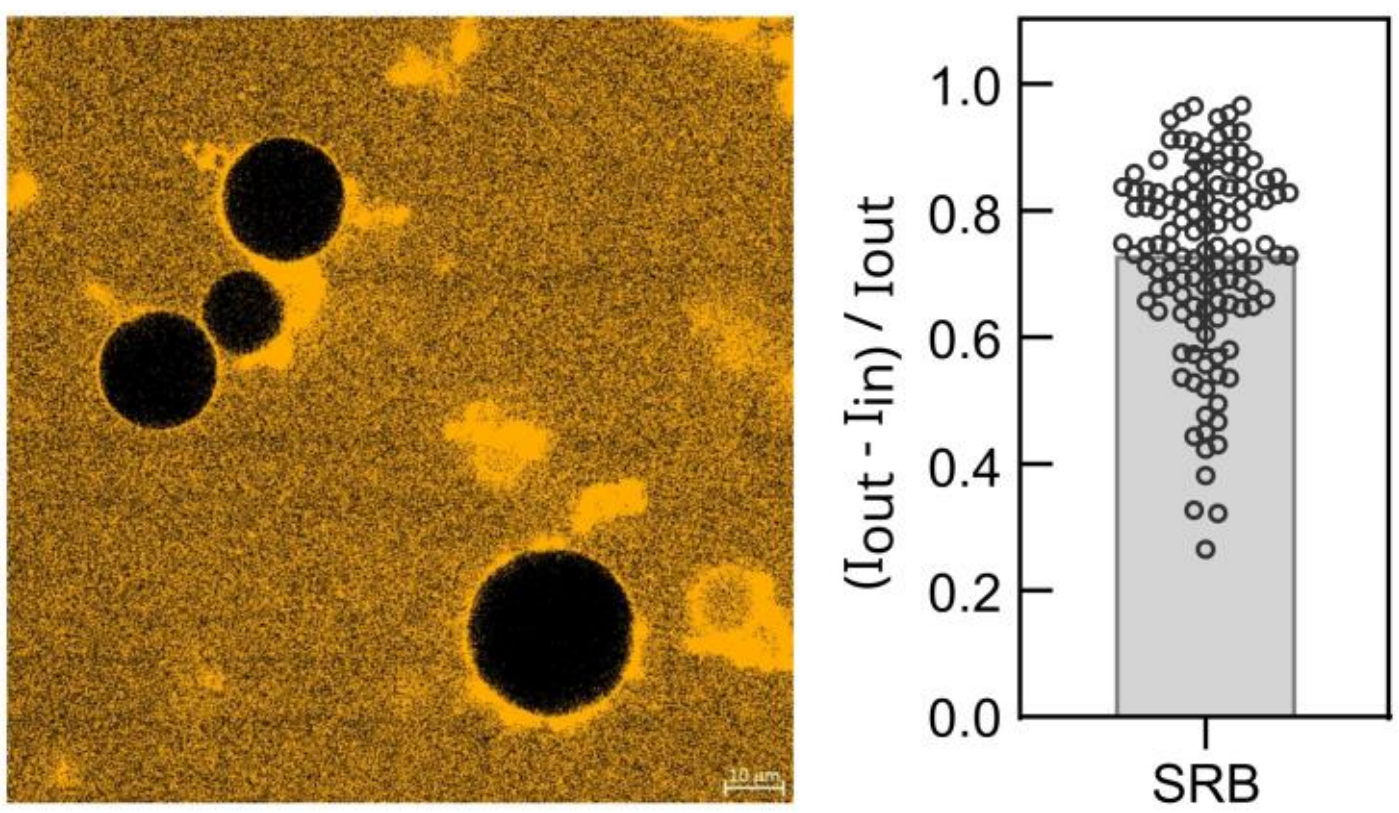

Figure S21. SRB influx assay following needle retraction. Marked reduction in SRB influx indicated successful needle retraction.

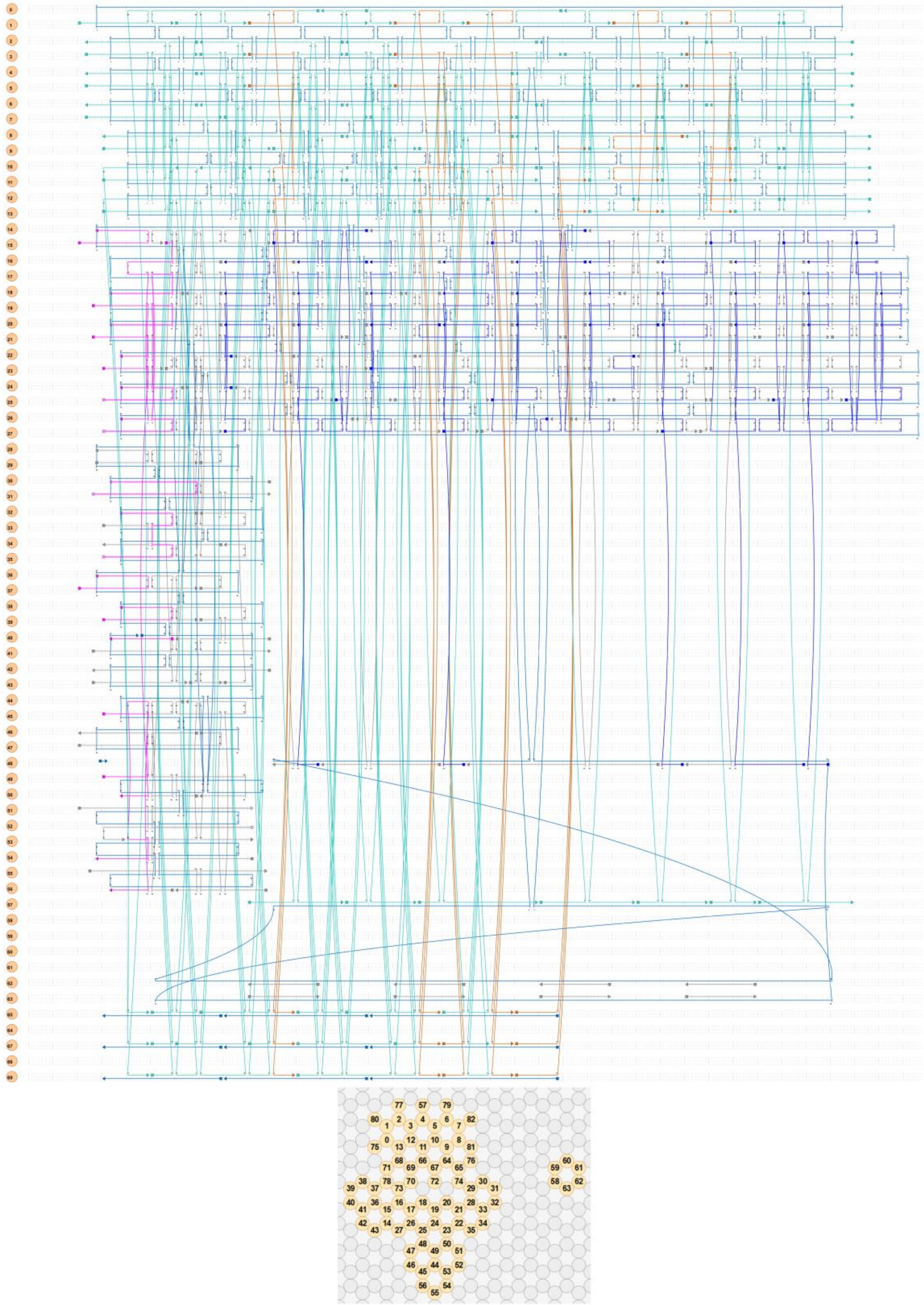

Figure S22. caDNAno design of the DNA origami in the DOS variant with blocked central channels in the needle. The cross-section of the structure is shown at the bottom of the strand diagram. Both were generated using caDNAno. The filled hexagonal lattice follows previous designs[2], and the use of short miniscaffold strands to overcome scaffold length limitations is adapted from prior work.[3]

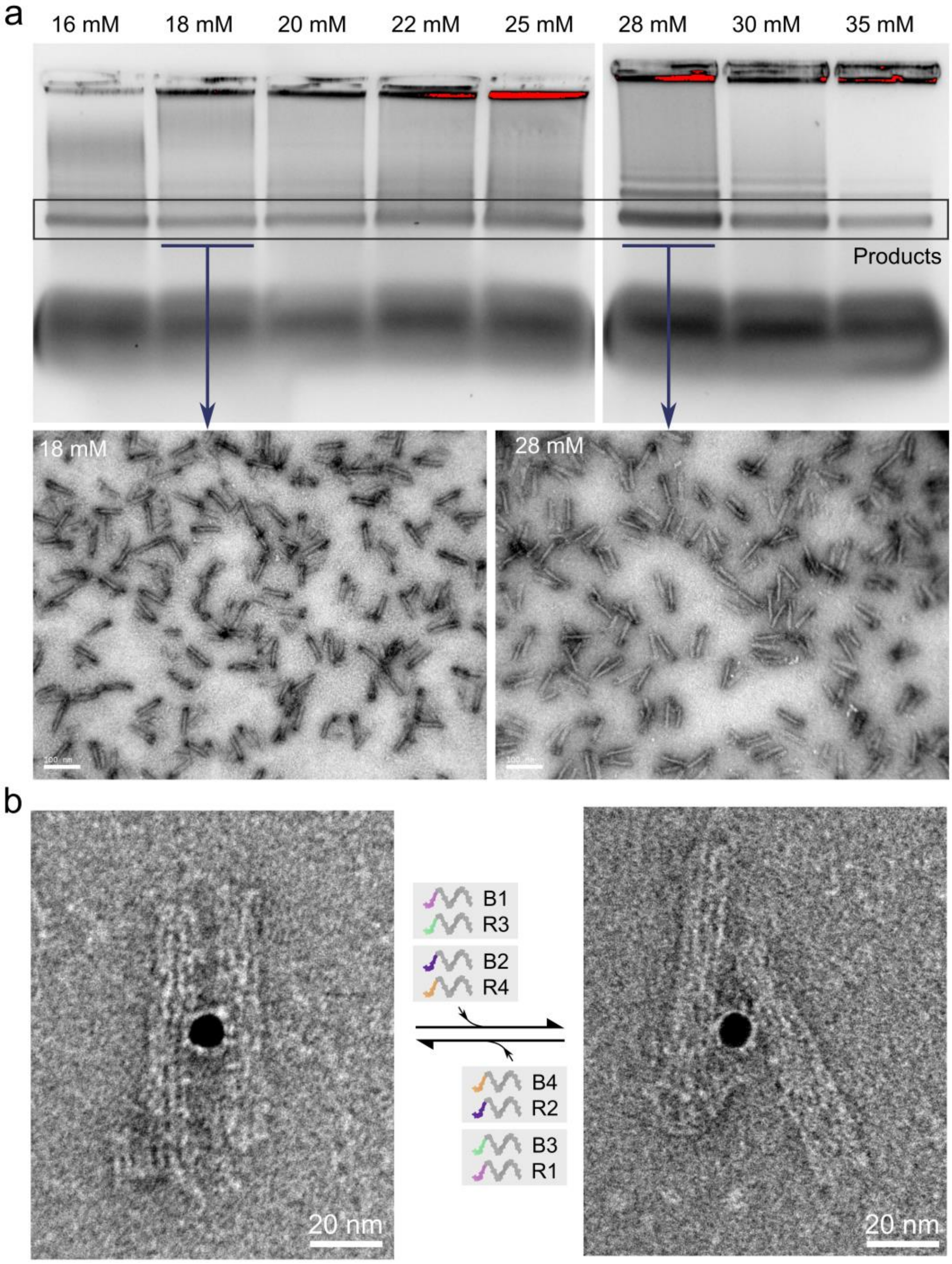

Figure S23. Structure characterization of DOS variants. a, Agarose gel electrophoresis (1%) of DNA origami assembled under different Mg^{2+} concentrations, with representative TEM images at 18 mM and 28 mM Mg^{2+}. The hexagonal-lattice close-packing induces increased electrostatic repulsion, necessitating higher Mg^{2+} concentrations for proper folding. TEM images show well-formed structures at 28 mM Mg^{2+}, whereas assemblies at 18 mM Mg^{2+} exhibit incomplete folding, especially in the hexagonal-lattice regions. **b**, Representative TEM images illustrating the reversible sliding behavior of the DOS variant.

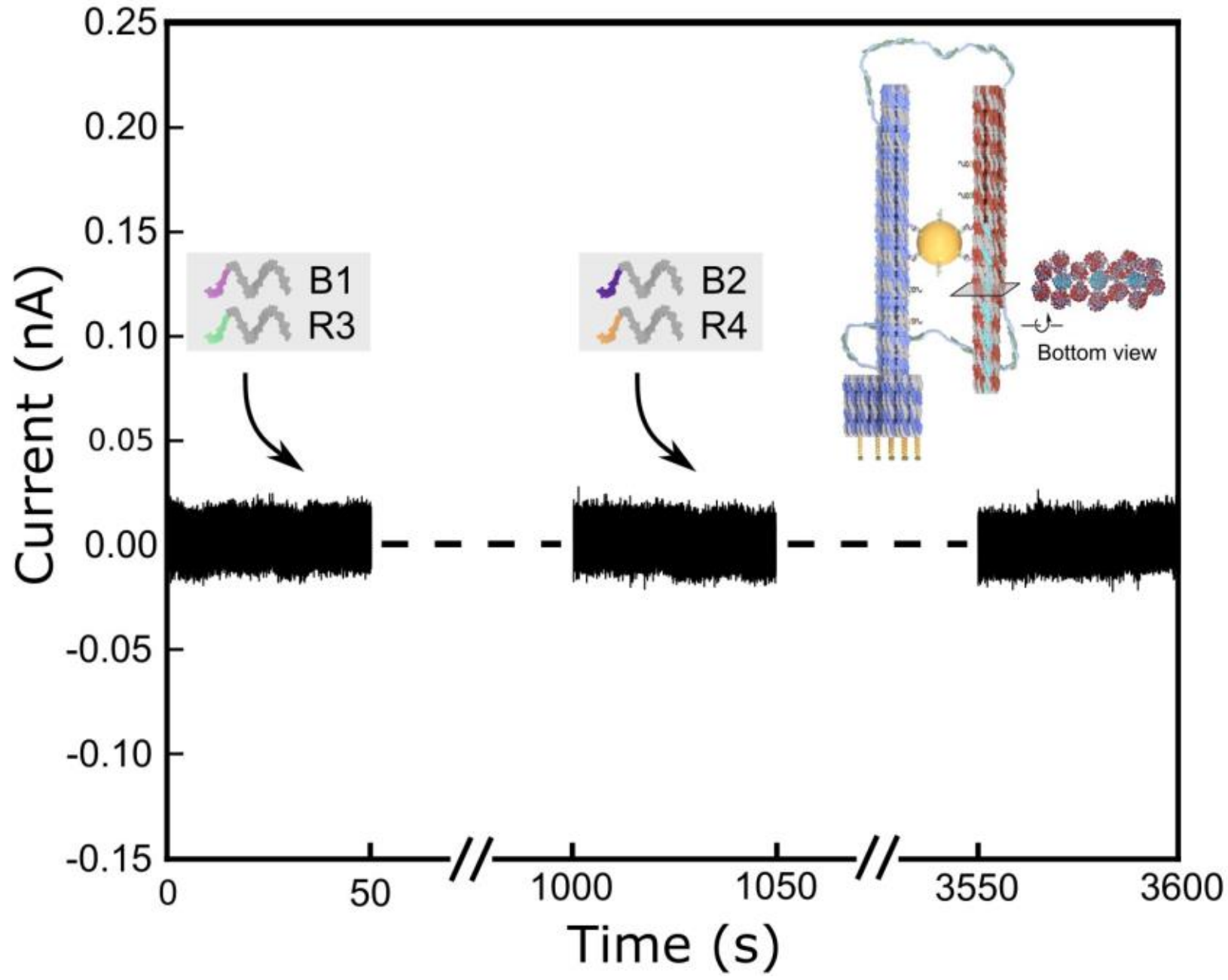

Figure S24. Ionic current recording of blocked-channel DOS. Ionic current recording of a DOS variant with sterically blocked central channels. No detectable current signal is observed under identical conditions. The measurement was conducted in 1 M KCl and 10 mM HEPES (pH 7.6) at an applied membrane potential of 50 mV.

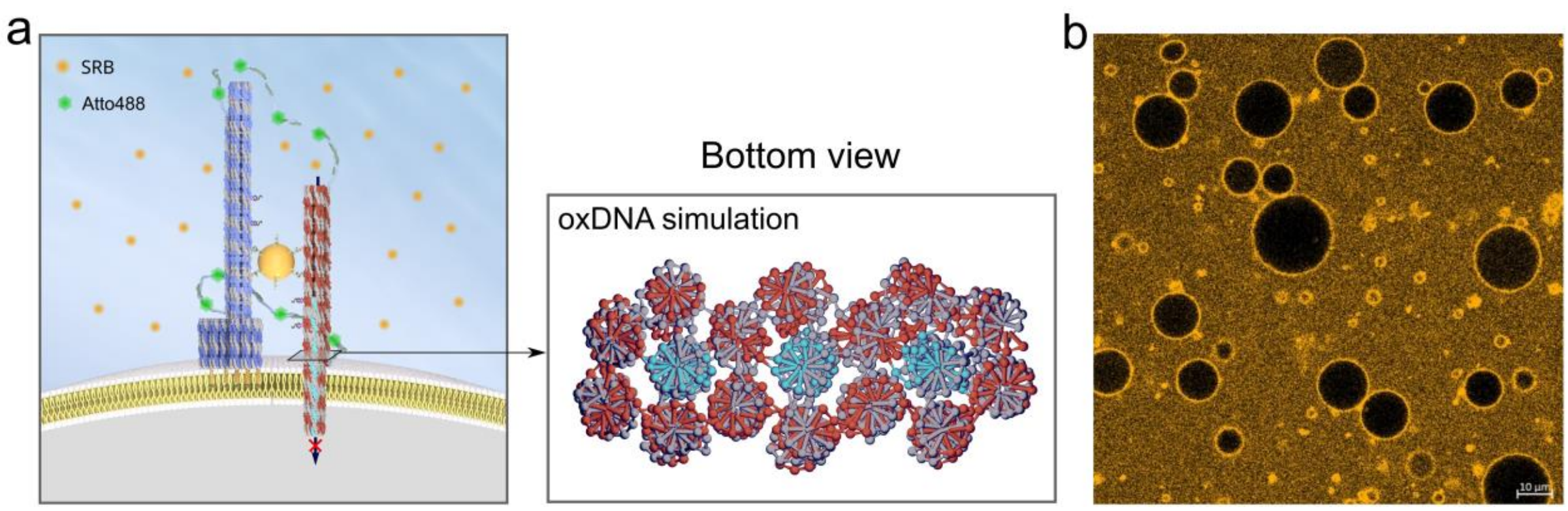

Figure S25. SRB transport assessment using DOS variants. a, Schematic of the blocked transport pathway in the DOS variant, featuring channel obstruction within the needle, accompanied by a bottom-view oxDNA simulation of the blocked lower channel segment. **b**, Confocal fluorescence image of DOS variant-bound GUVs after incubation with SRB.

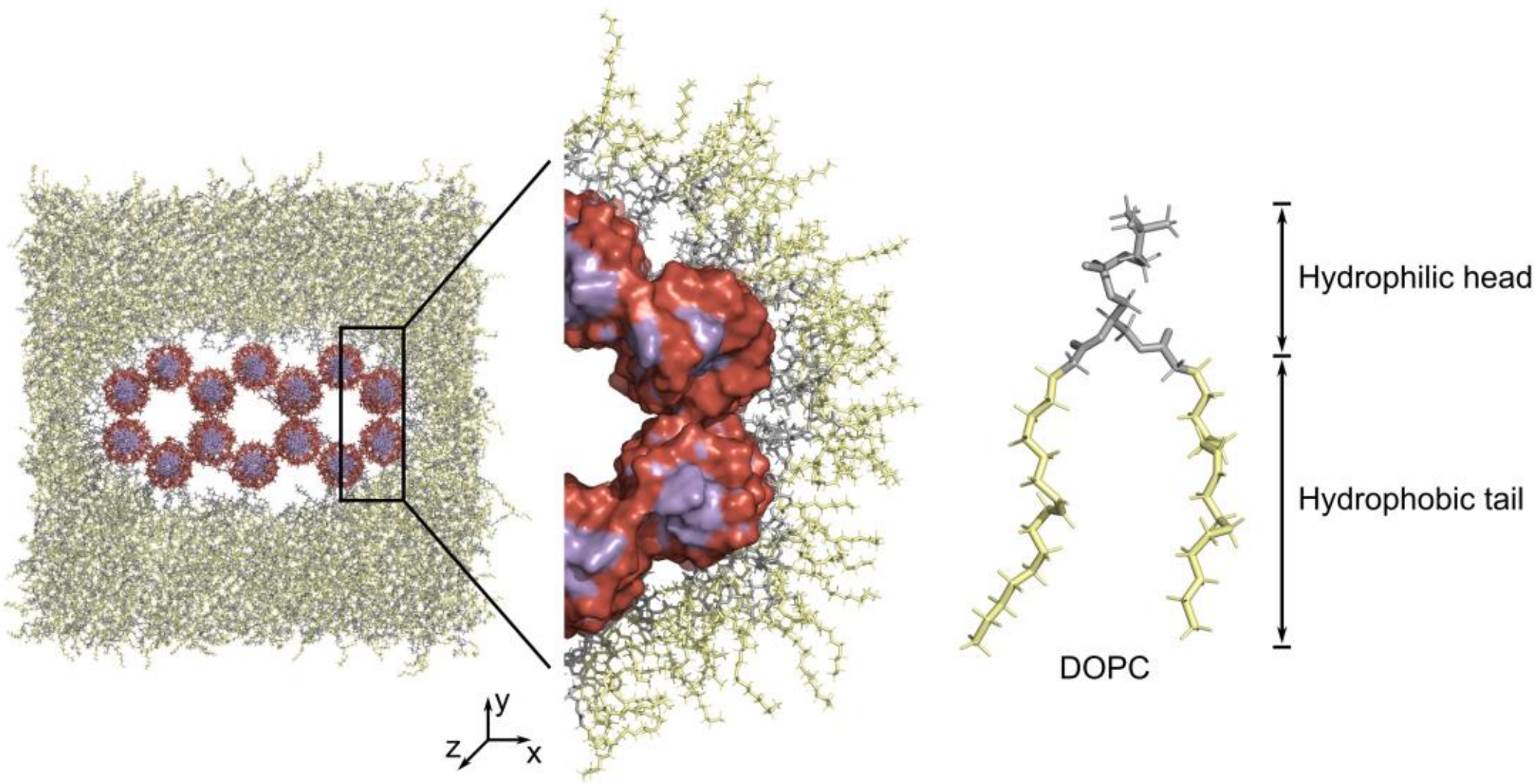

Figure S26. Orientation of lipid molecules around DOSs after membrane penetration revealed by all-atom molecular dynamics simulations. Hydrophilic head groups of DOPC preferentially associate with the needle surface. The all-atom molecular dynamics simulations were performed as follows: The DNA origami was converted into an all-atom model and embedded into a pre-equilibrated lipid membrane. The lipid bilayer comprised DOPC of 20 × 20 nm^2, generated using the CHARMM-GUI website[4-6]. The system was solvated with TIP3P water molecules[7] and supplemented with ions to achieve a 10 mM $MgCl_2$ solution, and additional Mg^{2+} was introduced to neutralize the system charge. All MD simulations were performed using the GROMACS software package[8,9] and employed the CHARMM36 force field parameter set[10]. Periodic boundary conditions were applied in all directions. Temperature was maintained at 300 K using the V-rescale thermostat[11], and pressure coupling was achieved at 1.0 bar using the C-rescale barostat[12]. Covalent bonds involving hydrogen atoms were constrained using the LINCS algorithm[13]. The simulation timestep was 2.0 fs. The cutoff distance of non-bonded interactions was 1.2 nm, and long-range electrostatics was calculated using the Particle-Mesh Ewald (PME) method with a 1.2 nm cutoff[14]. After energy minimization, equilibration simulation was performed with position restraints. Initially, position restraints with a force constant of 1000 $kJ \cdot mol^{-1} \cdot nm^{-2}$ were applied to the heavy atoms of the DOS and lipid membrane, followed by gradual removal of the membrane restraints, which allowed the system to adopt equilibrium configurations. To further accelerate the configuration equilibrium, the system was subjected to an annealing cycle using the GROMACS annealing protocol. Following a 300 ns NPT production simulation, the system was cyclically heated to 370 K and cooled to 300 K over 500 ns. Ten annealing cycles were performed. The final structure was visualized and analyzed using the PyMOL software (Version 2.0).

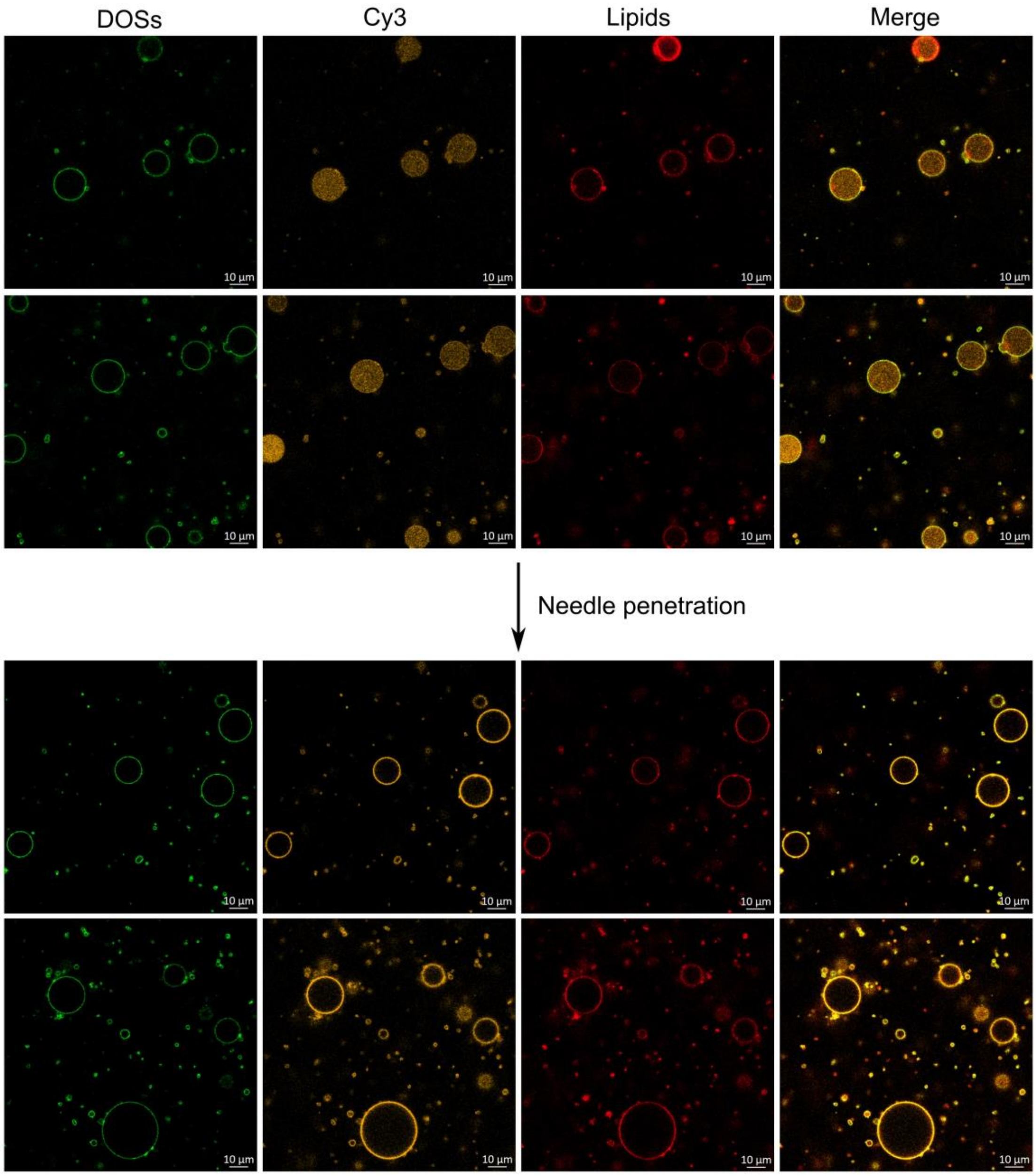

Figure S27. Wide-field confocal fluorescence images of GUVs before and after DOS needle penetration for the HCR reaction. Following needle penetration, Cy3-labeled DNA strands redistribute from a uniform luminal distribution to a membrane-associated, ring-shaped DNA cortex.

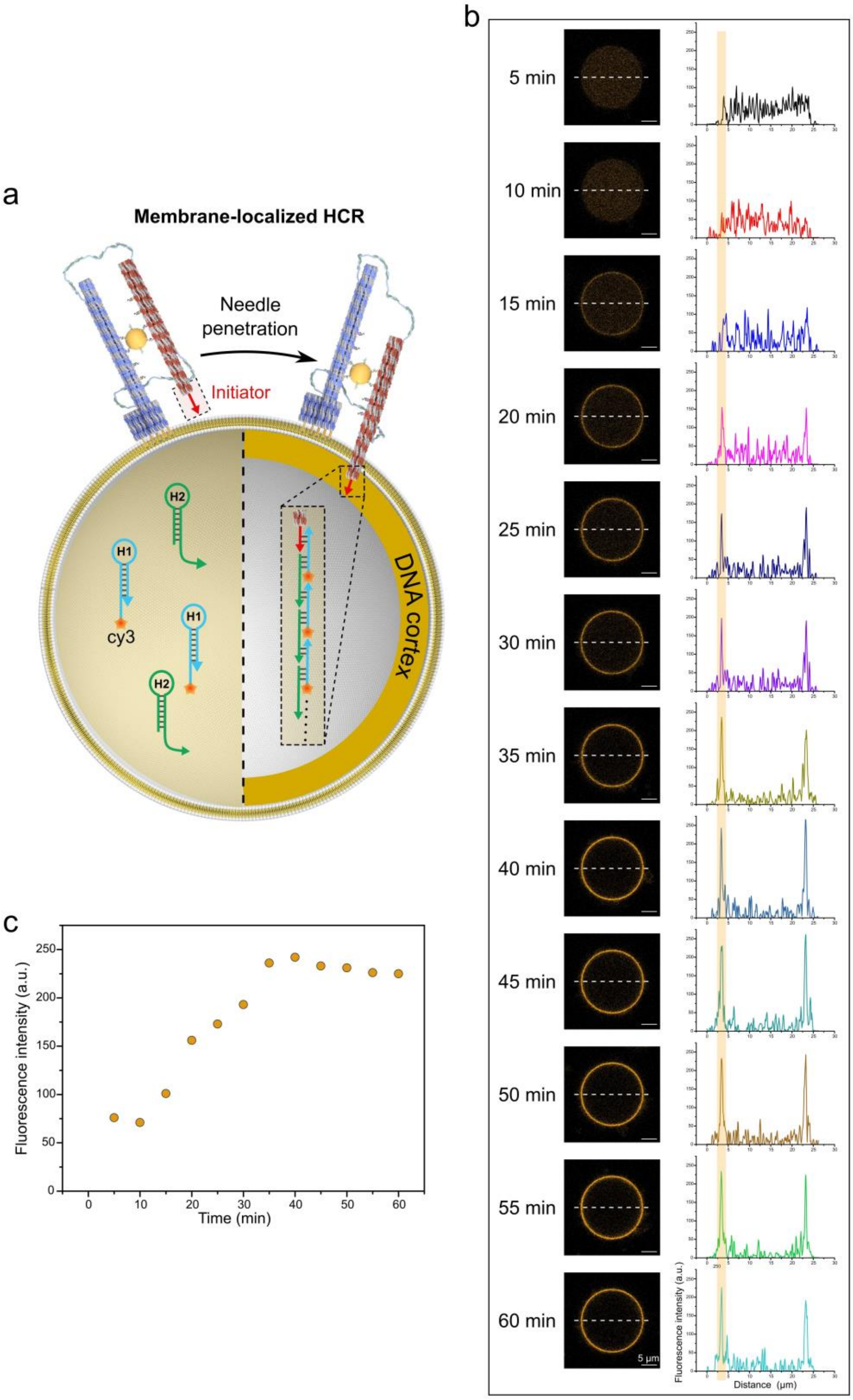

Figure S28. Time-resolved monitoring of DOS-directed membrane-localized HCR. a, Schematic of DOS-directed delivery of initiator strands and subsequent HCR within a GUV. **b,** Time-resolved confocal fluorescence images of a representative GUV following addition of the second pair of fuel strands. Fluorescence intensity profiles along the equatorial plane are shown alongside each image. **c,** Quantification of membrane-associated fluorescence intensity over time, extracted from intensity peaks at the membrane region as indicated in panel b. A

membrane-associated, ring-like fluorescence distribution becomes apparent at ~15 min after addition of the second pair of fuel strands.

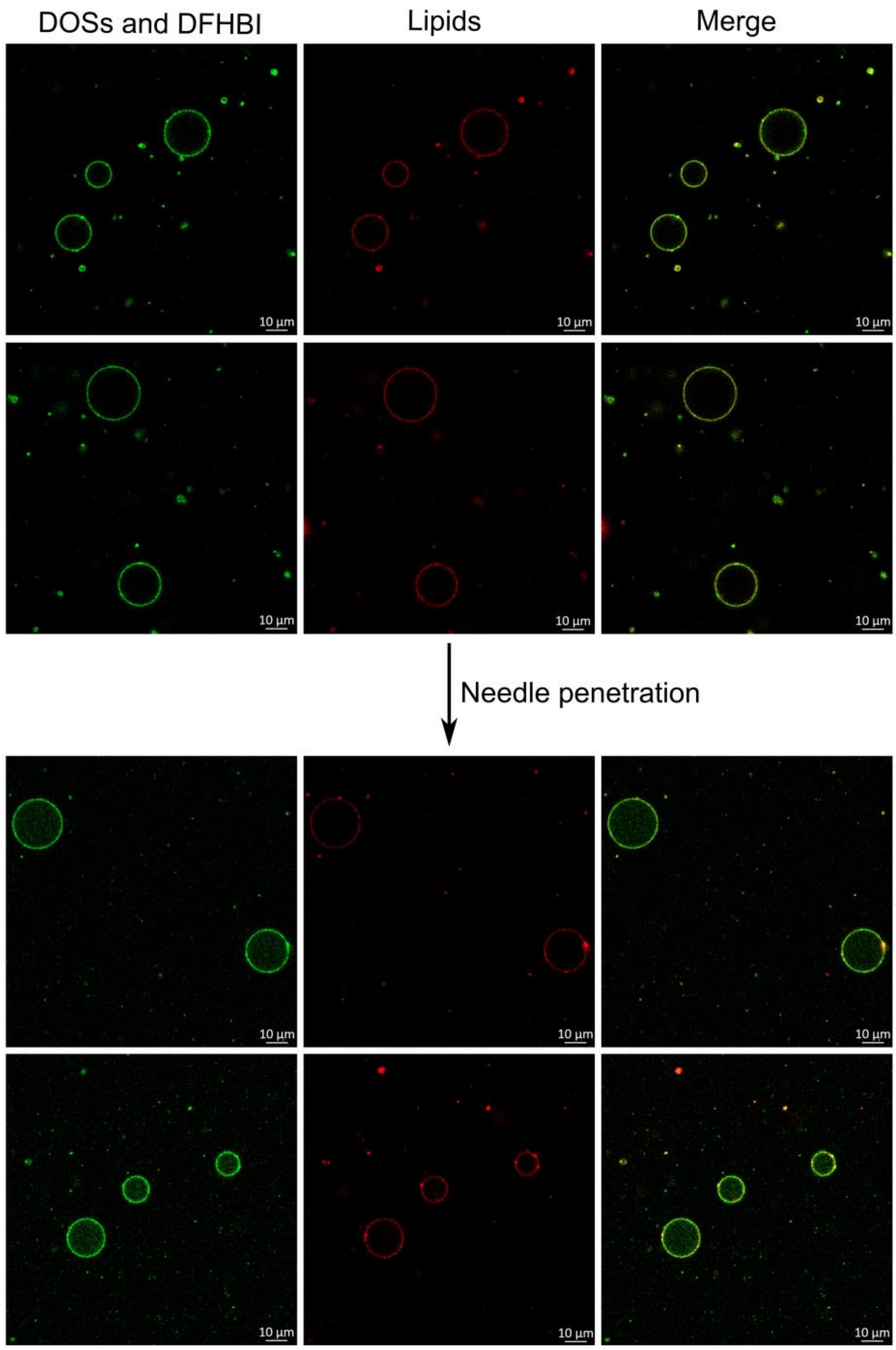

Figure S29. Wide-field confocal fluorescence images of GUVs before and after DOS needle penetration for Spinach RNA aptamer transcription in the presence of DFHBI. Following penetration, DFHBI fluorescence is observed throughout the GUV lumen, indicating successful transcription of Spinach RNA aptamers.

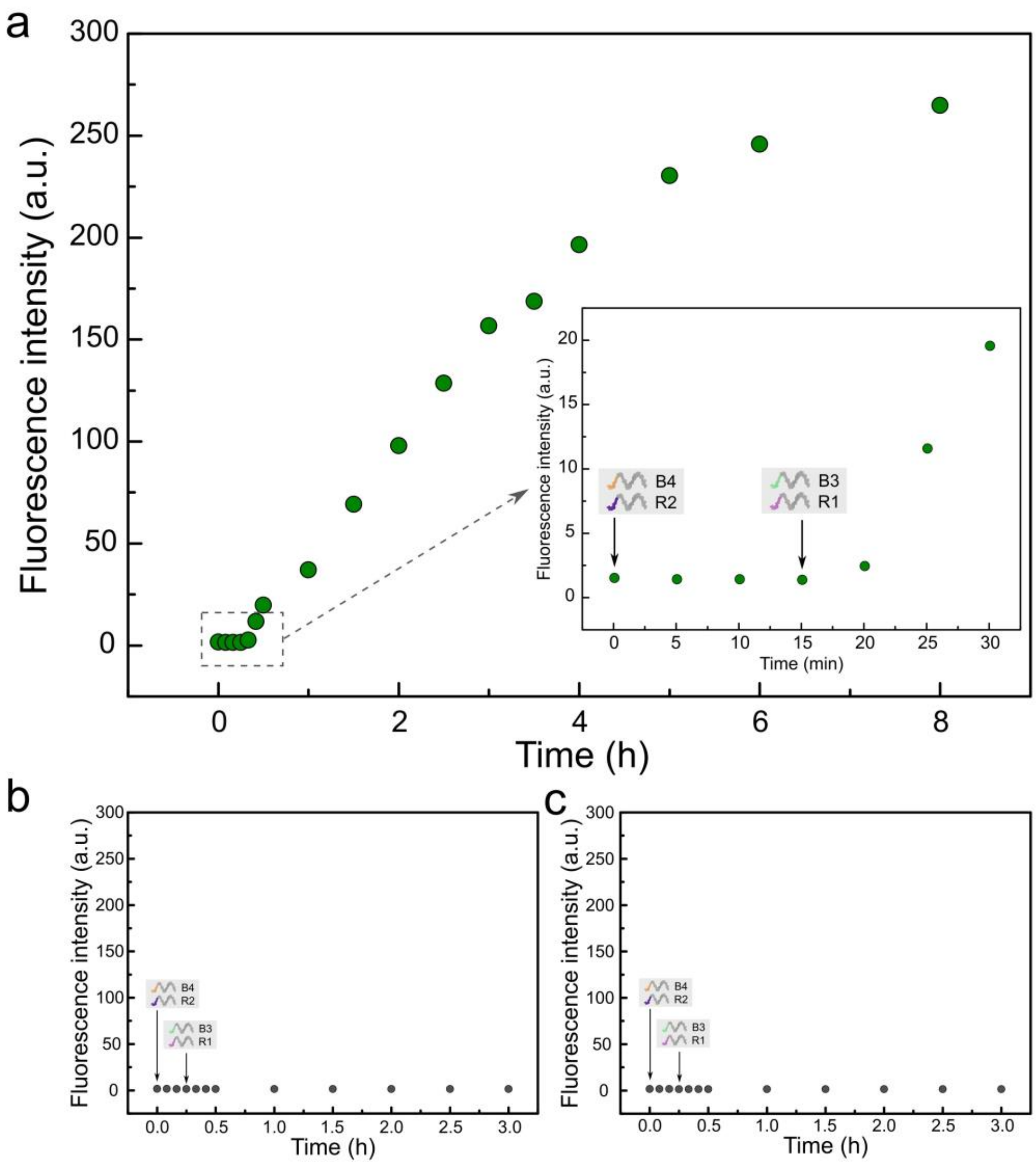

Figure S30. Time-resolved spectrofluorometric analysis of RNA transcription and control experiments. a, Time-resolved measurement of Spinach RNA aptamer transcription upon DOS activation using fluorescence spectroscopy. Fluorescence intensity at 506 nm (excitation at 470 nm) is plotted over time. A gradual increase in fluorescence is observed following addition of the second pair of fuel strands, with an initial rise at ~5 min and a more pronounced increase at ~10 min, indicating the onset of transcription. **b-c**, Control experiments for RNA transcription. Time-resolved spectrofluorometric measurements under control conditions. **b**, DNA origami frameworks without gold nanoparticle crosslinkers. **c**, DOS with GUVs prepared in the absence of DNA template. No significant increase in fluorescence is observed in either case.

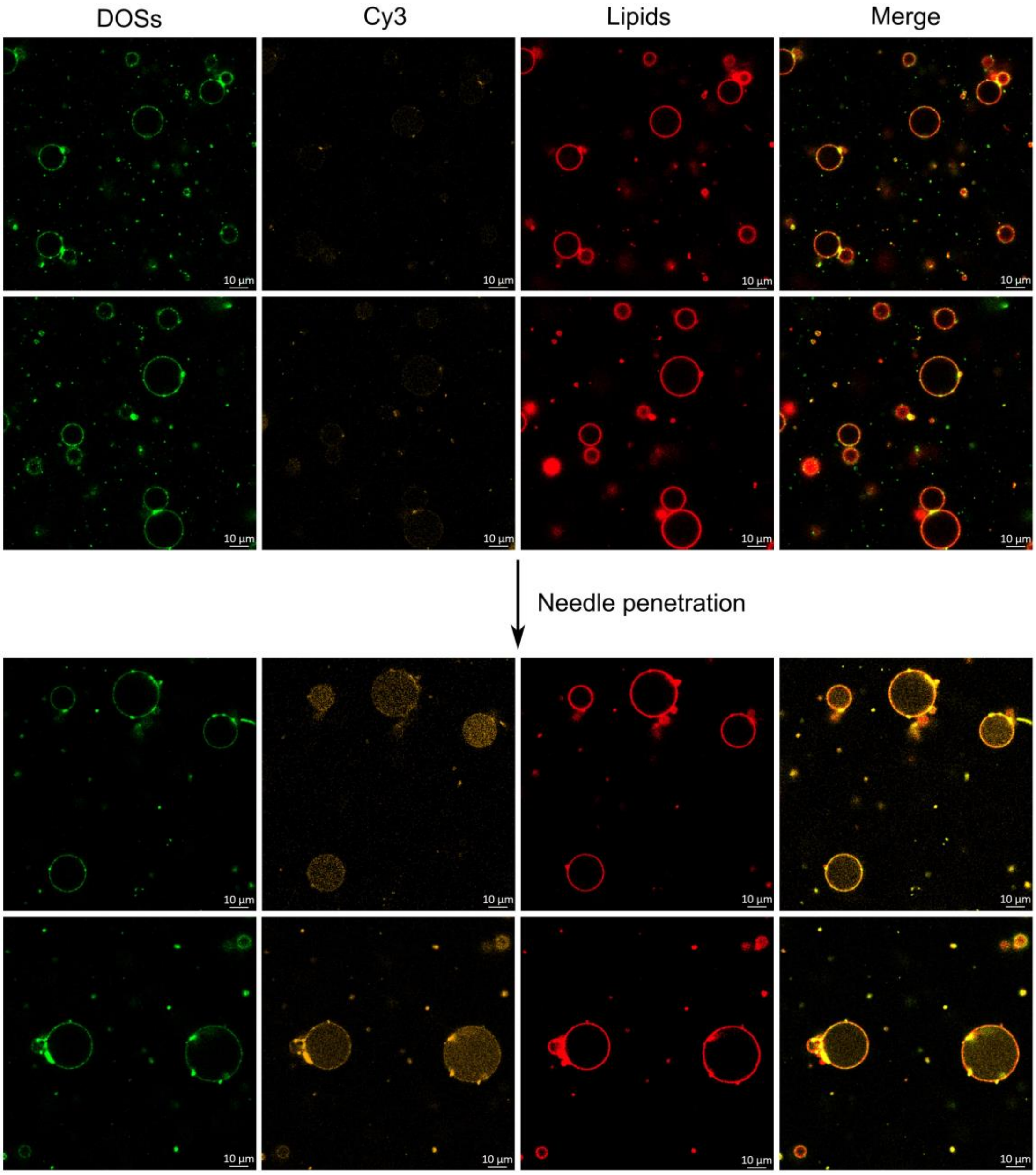

Figure S31. Wide-field confocal fluorescence images of GUVs before and after DOS needle penetration for DNAzyme-catalyzed RNA cleavage. Following needle penetration, fluorescence is observed throughout the GUV lumen, indicating successful RNA cleavage.

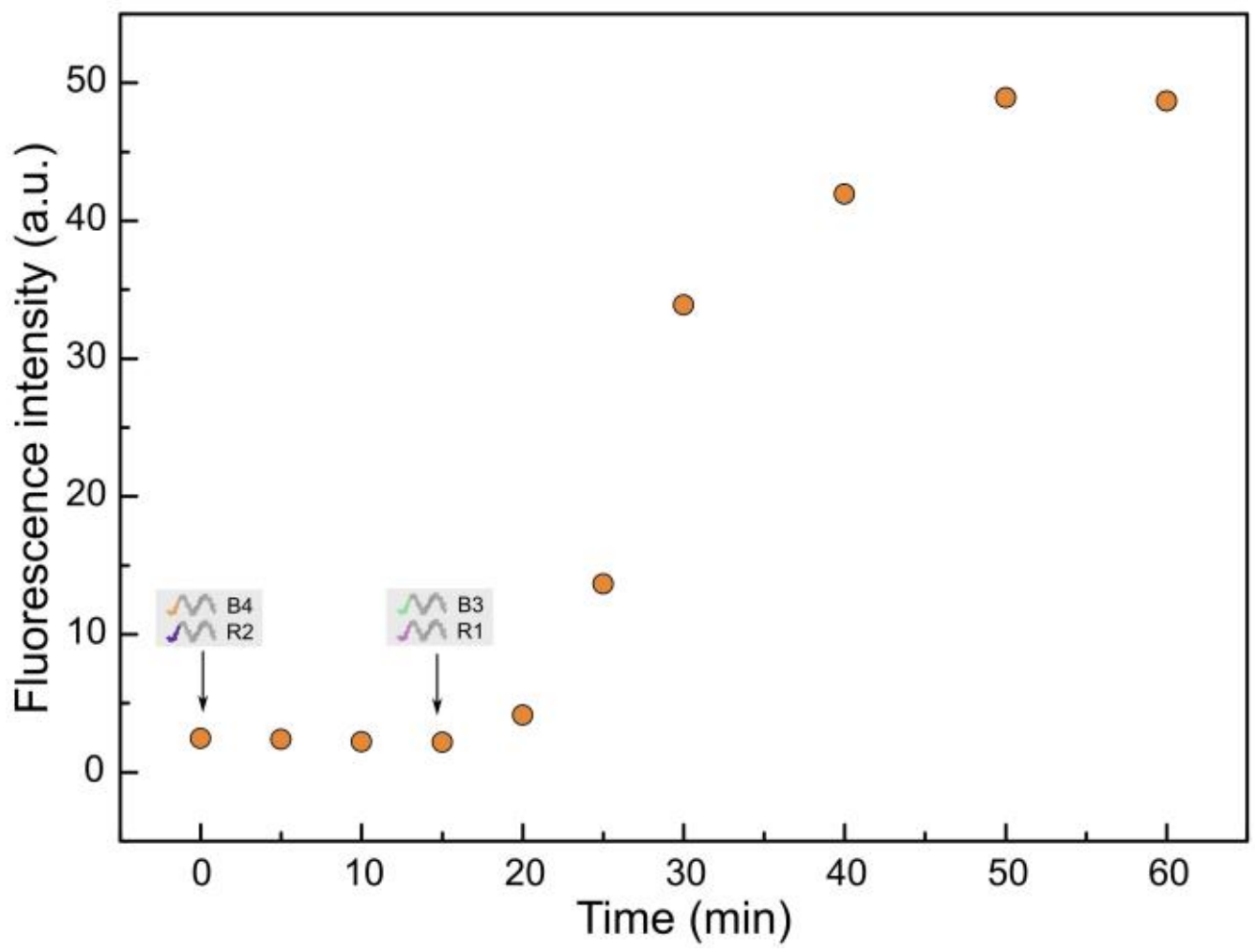

Figure S32. Time-resolved fluorescence measurement of DNAzyme-catalyzed RNA cleavage upon DOS activation using fluorescence spectroscopy. Fluorescence emission spectra were recorded over time (excitation at 550 nm), and the intensity at 570 nm was extracted to generate the time-resolved profile. A gradual increase in signal is observed following addition of the second pair of fuel strands, with an initial rise at ~5 min and a more pronounced increase at ~10 min, indicating activation of RNA cleavage.

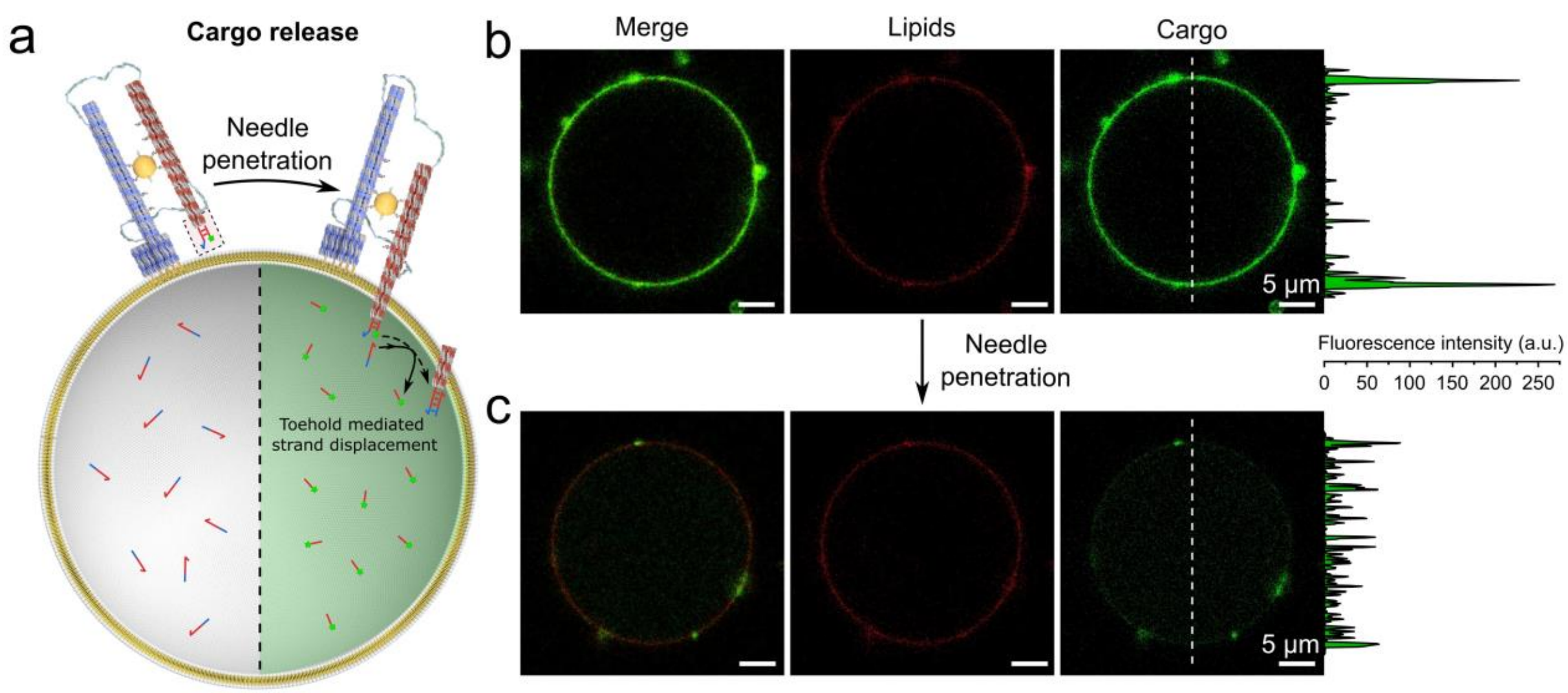

Figure S33. DOS-directed cargo release within GUVs. a, Schematic of cargo tethering at the DOS needle tip and its release into the GUV interior via toehold-mediated strand displacement. Four DNA strands extended from the needle tip were used to tether Atto488-labelled DNA, while the corresponding displacement strands were pre-encapsulated inside the GUVs. **b,** Confocal fluorescence images before needle penetration, showing cargo fluorescence at the GUV membrane. **c,** Confocal fluorescence images after needle penetration, showing decreased

membrane-associated fluorescence and increased fluorescence within the GUV lumen. Fluorescence intensity profiles along the equatorial plane are shown alongside the images.

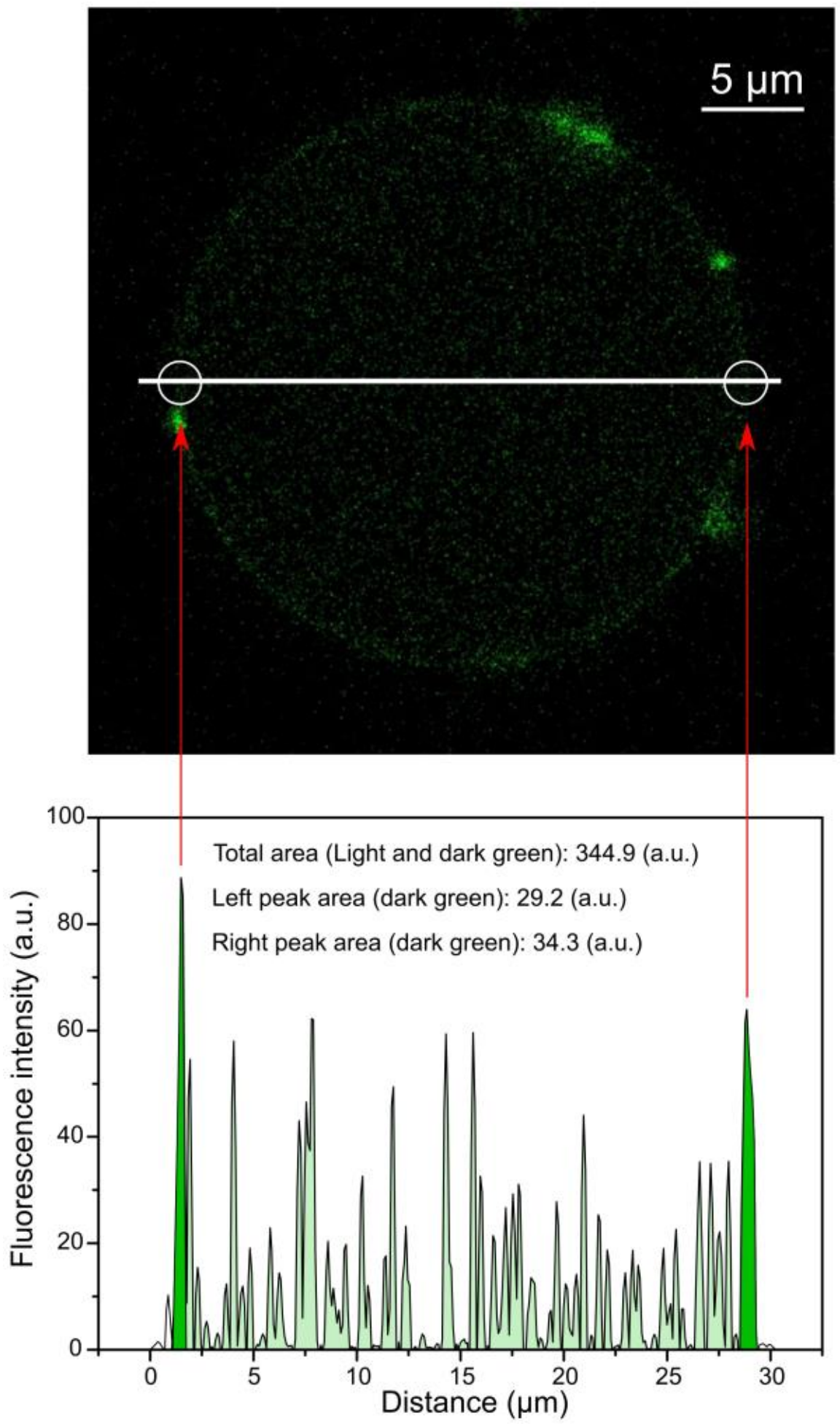

Figure S34. Quantification of cargo release based on fluorescence intensity profiles. A fluorescence intensity profile along the equatorial plane of a representative GUV was analyzed using ImageJ to distinguish membrane-associated and lumen-localized cargo signals. Two intensity peaks at the membrane positions correspond to residual membrane-associated cargo, whereas the remaining signal is attributed to cargo released into the GUV lumen. The total integrated intensity across the entire profile is 334.9 a.u., comprising both membrane-associated cargo (dark green) and released cargo within the GUV lumen (light green). The membrane-associated peaks are 29.2 and 34.3 a.u., respectively. Based on this analysis, the released fraction is estimated to be ~80%. This value should be regarded as an approximate proxy, as quantification at the level of individual DOS devices is limited by the optical diffraction limit inherent to confocal microscopy.

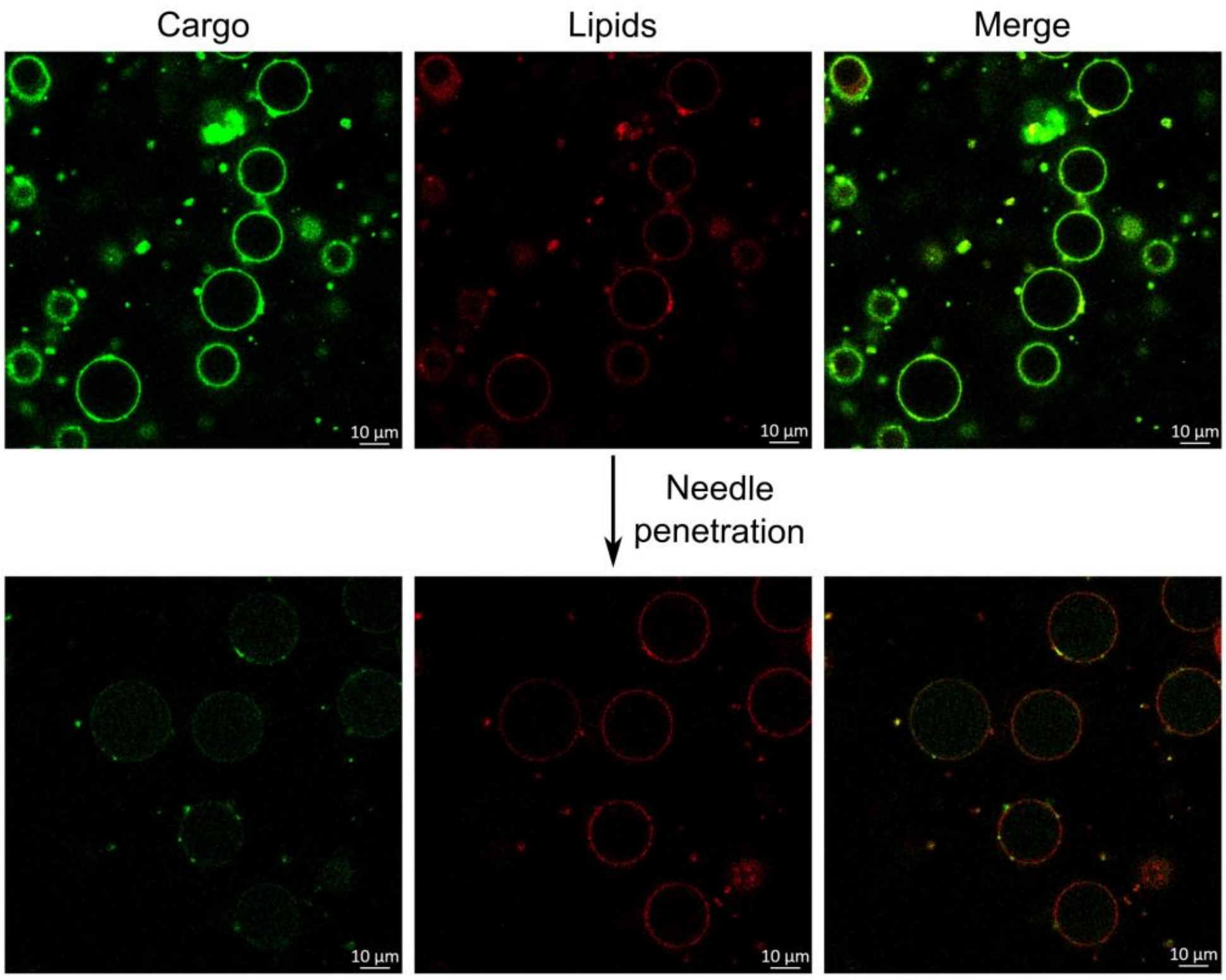

Figure S35. Confocal fluorescence images of GUVs before and after DOS-directed cargo release. Images show redistribution of Atto488-labeled DNA cargo following needle penetration. Prior to penetration, fluorescence is localized at the GUV membrane, reflecting cargo tethered to the DOS needle tip. After penetration and incubation, fluorescence decreases at the membrane and appears within the GUV lumen, demonstrating release of the cargo into the GUV interior.

DNA sequences

Table S1. Sequences of DNA origami component of the DOS.

Start	Sequence (5’ - 3’)
27[119]	TTAGGTTTCACAGTACCAGCGTGGTGCTCGGCAGCACCGTGC
26[149]	CCGATGCTGAGGTGTCCAGCATCAAACCAGCCGCAAGA
16[160]	AAGTTACCAGAAACTTACGGCTGTAGCTATCTTACCCGAACA
27[182]	CGAGAAACCAGCAACCATCCTGAATCTTATAGCAGCCTTTAA
25[214]	CTTCATAATTTTAATTTAGATAACCCACAAGAACAGGGCGTCAAA
25[235]	CTCAATAAGAAATTTAAAATTGAGCGCTAATGGAGAATAAGAAAC
27[56]	GAAGAGTGTCGCTAGTACGCCAGAATGCTCCGTATAACGTGC
25[88]	GTGTCGTAATCATAGGTAAGCGGTCCACGCTCCCTGAGCAGCTGA
16[97]	TCTGAGACGGGCAAAGAGTTGCAGCGGTATGAGCCAAAGGTT
48[125]	CAAGTTTCGTGAGCTCATACCGCGCAGCCTCCGGCCTTGTAGAACGTAC
15[133]	CCCACGCGCGGGGTATAACTATATGTAAGAAACGTTCAGCAGCGGAACG
14[160]	AGCAAGATGCAAATCCAATCGCCATTACAACGCAATTAAGAC
48[188]	TCACGGAGGGAATTTATTCATAACCAATAGCAAGCAGCTACAATTTTAA
15[196]	ACATAAAAATTGAGCAAATATATTTTAGACTAGAACTAACGAGCTATTT
15[217]	TTAGACGATCAGAGCATCTTCTGACCTAATAAACACCTAATTAGCCTTA
15[238]	ACACCCTAGAGGGTTGGTTTGAAATACCTAAATAATAAACAGCCGACTT
24[58]	AATTACTTCTTTGATTGGGCCTGAGAAGTGT
15[70]	CGCCTGGGGTTTGCGAATTTATCAAAATCATGGTCTGGTTTTGCGGTTT
14[97]	CGGTTGCCTGAGAGACTACCTTACCGAGTGCTCGTAATCGTT
20[118]	CCAGCGGCAGTGTCACTGCCGATGCTGAGTCTCGTCGCTGAG
25[133]	ATAGCAGCACCGCTTACATCGCATCCTCCGGTCCG
22[174]	GCGCCAAAGGGAAGGAATTATCACCGCAAACGTAGAGAAACG
20[181]	TGGCAACATAGAAAATTCAATTTACGAGTCATTACCGCGCGG
48[223]	AGAGATAGACTTTCTCCGTGGTTATACATTTCGAGATATAGATTTCATC
48[230]	ATTTGTGTAAAGCCATGTAATTTCTAAGGAACAAG
16[244]	ATCCCAATACAAAAGGCGTTAAACAGTAGGGCTTAGCCATATTAGCGAAACCAAGT
22[58]	TGACAAATGAAAAATCTCGTAGTAATAACAT
48[83]	CATTTTCCTGTTTCCACAACACATTAATGTCGGGA
23[98]	AGCGGCCAGAATGCTCTGCCAGCACGTCGCATCAGGCCCCCT
1[39]	TTAATGCAGATACATAACGCCATTTTTT
13[35]	ATATGCAAATGCTGAGGCATAAATAGCGAGAGGCTTTTGCTTTTTT
11[35]	GAGAGTAAGCAAACGCCAGAGCATAAATATTCATTGAATCTTTTTT
7[17]	TTTTTTCCCCTCAAATGCCAAAGCGGATTTTTT
2[139]	AGTGCCTACTGGTAGTAGCCAGCTTTTTAGGAACGTGGGCTT
2[181]	ACAGATGTCGCCTGGATCGTCACCAGTAGCCCTCATAAGGCT
8[216]	GTCGCTGCCGACAAAATTGTATTCACGTACTTTCACCAGACGTCATTAC
2[76]	ACCCTCATGCGGGATTTTGCGGATGGCTGTTTCATCTGGCTC
9[46]	GGAGACCGGACCTTTAATGAATATACTAAAGGACGTTGGGAAGAAAACA
7[200]	GACCAGGGAGTTAAAGGAGCTTGATGCTTTCGGAA
7[84]	TACAGGCAAGGCCTTTCTACTAATAGTACTGAAAAGCTATATTCGC
25[168]	GACTTGAAGTAGCACAAGACAAGAAACAATGAAAT
16[76]	TTGCCCTGCCAGGGATCTTCTGTAAATCCAATAGTCCCAGCA
25[105]	CTGTTCTCCCCGGGTTTTAACCAATCCGCCGGGCG
27[130]	TATCATTGCAGGCGCTTCTCGGTGGTGCCAT
27[193]	TTTTTAAGCCCAATAATAGACAGAGAGAATA
21[210]	TCAATAATTTTTATAGGCTTAATTAGTTGCGTCTTTGTTTAAAAGCGCA
21[231]	TTATCATTCATCGAAACGCGAGTTTTGATGCCAGTTCCAAATTAACTGA

Start	Sequence (5’ - 3’)
43[18]	TTTTTTTGGGGTCGAGGTGCCGTATGGTTCCATAG
43[56]	AACCCTAGGCGAAACTTTGACGAGCAAC
22[111]	TCTGTGGTGCTGCCCAGACGATCCAGCGTGCCGGTATGCCGGTTCA
23[161]	TTGAGGGAGACAAATTTTGTCACAATCAATATAAAAAAATACGGCA
23[39]	AATACCCTCATATCCAGAACAAAGCTGAGTAAACCGTTATCA
13[119]	AACCAATTTGTTAATAATACAGGAGTGTTGAGTAA
13[140]	AAATAATTCGCGTAACACCAGAACGAGTCCCCCTGCGGGGTC
13[161]	ACAGACACAAACTAAAGGATTAGGATCAAAACATGAAAGTAT
11[193]	TTGGTGAGAAATCTAAACAAAGCTGCTCATTGACCAGGAAAGAGG
13[56]	TCTGGAATAGAGCTATCATAACCCTCTTTAAGATT
11[88]	AAAATTTAGTGATTCCCTACCTTATGCGATTTGCAATGTTTTAGA
13[98]	CGTTAAATCAGCTCTCAACTTTAATCATAGGTAAACAAATCA
21[154]	AAGTTTAAGGGCGACAGCCTTTAGTGATTCCGGCAAAGGAAT
23[196]	TAAAGTACCGACAAAAGAAAAAAACCAA
18[223]	AATCAAGTCCGGTATTAGGCAAGTAATTCTGTCCAAACAATATCTTTCC
18[244]	GCGGGAGGGCGTTTTTAACAAACAATAAACAACATTGCAGAAACGGGTA
20[76]	AACCTGTAACACATTAATTGCGTTGCGC
18[118]	TGGTGTGGTTACCTGGGGGTTGGCGGGCCGTTTTCAAGCGCCTGTGCAC
18[181]	AAGAACTATACATATAAAGGTGTAAATATTGACGGTATATGGTTTACCA
18[55]	TTTTATAGTAGCAATACATTTTGCAAAAGAAGATGTTGC
22[76]	TAACTAGCATCACCATGAAACAAACACGAGCCGGAAGTGAGC
20[139]	TTTTTTCTTGCCGTGAAGGGTAAAGTTAACCCCGTAAAAAAA
20[160]	CAAAGACACCACCGATAGTATGTTAGCAGTTGGGCCAACCGA
20[202]	GTAGGAACATGTAGATAATATCCCATCCAAGAATA
56[41]	CGTTATTGTAAAACCCTACCACTTCTGATATAATCGGCA
20[97]	GCGCCCGCTTTCCAGAATCGGCCAACCGCGCTCACAGTGTAA
34[55]	TTGAAATAAAAGGGACCTGAAATATCAACAACAGTAACA
16[55]	TTTCCTCAGGAACGTTAATTATCAATATATGTGAGAATT
48[118]	GCCTTTAGCGTCAGACTGTAGTACGTGC
16[118]	GGCTGGTATCCCTTTGAGGATTCGCGTC
18[139]	TGCCGGACAGAGCAACATAAAAAAATGGCTCATCG
48[181]	AAAAGAGACGCAGAAACAGCGAGTCACC
16[181]	AAGTAAGGGAATACAATCACCGCCATTT
18[202]	TGCACCCAAATCAGCCAGTAATAAGAATAGGTATC
20[223]	CAAGCCGTCGGCTGGATAAGTCCTGAACAAGGTAAGAGGCATAATT
16[223]	GATTTTTTCCAGAGCCGGAATACCAGTA
20[244]	ACCGCACTCCAAGACGCGCCTGTTTATCGACGACGCGCCAACAACG
21[91]	TCACTATTGGGGTGCCTAATGAGCATAAAATTCCACTGT
23[140]	GCCGCACAGGCGTTGGTTGTGGCTCATTAAGG
18[160]	TCCTTATTACGCAACAGGAAACCGAGGACATT
18[97]	AAGCGCGGGGAGAGTCTTTTCACCAGTTCTCG
18[107]	GCACATAAACAATGGGTGGGTCACTGTTGCCTCGCACTCTCCGGC
16[139]	ATGCCAAGGTCTGGCACCAATGAAACCCGGGT
18[170]	TGATAATAACCAGATAGCGAAGCCCTTTTTAAAGAGCAAAGAACG
16[202]	AATGAAAACCAACGAAAGCCTGTTTAAGACTT
18[44]	GTGTTTAGACGTTAGAAGAGTACTATGGTTGATCCTGTACGCTGA
29[19]	AACGCTCATGGAAATACCATTGCAACAGGAAA
32[48]	AAAACATCGACCAGAGTATTACCGCCAGCCGTCAGTATTAAC
8[104]	GTCAAAAGGAGCAAACAAGAGGTCATTGTTGAGAGTTAATGCATAT
29[49]	ACCGCCTCACGCTGTATGGCTATTAGTCGAACTGAACGAACCACTTTTT

Start	Sequence (5' - 3')
30[69]	TTTTTCAGCAGAAGATAAAACATACCGATAGCCCT
33[21]	TTTTTAGATTCACCGGCCAACAGTTTTT
20[55]	CACTTGCCCAGCAGACCTCAAAGCGTAAGAATACGAATATTT
41[18]	TTTTTCCCACTACGTGAACCATGGTAGGGTTTATTAAAGACACCC
40[69]	TTTTTAAAGGAAGGTGGCAAGACGCTGCGCGTAACCACCAACGGGCGCT
11[140]	ATGTGAGCGAGTAGTGTCTGGCCTTCCTATAAGTTGCAAGCG
11[161]	TGTACCGGGATAGCCGTCGAGAGGGTTAATTAGCG
7[182]	TCACCCTCAGCAGCTTCCATTGCACCAACAAA
9[203]	AGTTGCGAGGCTTGAGCATCGGAACGAGAAGACTTGGCAAAAGCGA
9[35]	AAAGATTTCAGAAGTTTAAACAGTTCAGGAATCGTGGGGTAAAAAC
11[56]	TTTTGATTTCAAAGATGTTTAGACTGCCCTGTTTA
37[49]	GCCGCGCGCTACAGGGCGCGAGGAAAGCGCGAAAG
39[49]	AGGGCGCGAAGAAACGGCGAACGTGGCGAGTTTTT
43[46]	ACTGAGCTTGACCACCCAAATCAAGTTTTTTTTT
24[44]	ATTACATAAAATTTTCCGATTAAGTTGATGGAAGC
42[69]	TTTTTGGGAGCCCCCGATTTAAAATCGG
54[64]	TTTTATAATACATTTGAGGATTTAGAAGCAGGCTAATAGATTAGGTTA
47[14]	TTTTTAGGTTTAACGTCAGATAATTGCGTAGATTTTCTTTTT
46[48]	AATAAAAATAAAGAGAATATACAGTAACAGTACCTGAGAAAC
26[156]	AGCTGCCGCCGATCAAACTTAAATTTTAATCAGTAGCGACAGAAT
25[196]	ATATGCGTGAAGGGATAGCTC
18[76]	GCGTATTCCAGCTGTATCAAGAAAACAATGAATAACCTTGAGGGTCATA
26[93]	AATAAATTGTCGCGTTTTCATCGG
50[48]	AGGAAAAATAATGGAACGCAGAGGCGAACAATTACCTGAGAA
52[64]	TTTTAAATATCTTTAGGAGCACTAACAAAACAATTCATCTTGAGGAAGGTTATCTATTTT
51[14]	TTTTTGATTATCAGATGATGGTTATCATCATATTCCCACCAG
56[68]	TTTTTCGTATTAAATTGCTTGTTACAAAATCGGGAGCCGTCAATAGTTTT
44[44]	GAAAGCGGATTCGCCTGATCCTTTCAAACAATTCGACAACTTTT
55[17]	TTTTTTTTGAGTAACATTATTATTAGACTTTAGCCCGAA
11[98]	TAGCAAATATTTAAATTAAATTTTTGAACAATATGCGGAGAGATTAAAG
11[119]	ACGTTAAGCCCCAAAACAGGAAGATCGCTGGCTTTTGATGGCGCAGTCT
57[126]	CTGAATTTACCGTTCCAGTACCGCTTCT
4[167]	GGGTTTTGAAACCAGGCAAAG
13[182]	CGTAACGTAGAAAGATAAATTCAACGGACGCCATTCAGGCTG
13[203]	CGTCTTTACAGTTTGACCTGCTACCAAGGTTGGGA
13[224]	ATGAATTGATTTTGCCGGAACATCTTTGGGGCCTC
13[77]	AACAGTTTTGACCAGAAGCCTATGACCCTTCACAAACAAATA
6[124]	GCCTGGCTATCAGAATCGATGAACGCGCATCGTACAGTATCG
9[140]	GATTGACCGTAATCATAACAACCCGTCGCCAGCTTTGAGGGG
9[161]	CCACCCTCTCAGAAACTCAGGAGGTTTACGTGATATAAGTAT
9[56]	AAGACTTTCTTTACGAATGACCATAAATAGGATAG
9[98]	TCTCATATGTACCCAAGATTGTATAAACGCTATTTCCTGAGA
62[125]	CAGAGCCACCACCCTCAGAGC
62[167]	AACCGCCTCCCTCAGAGCCGC
62[209]	TCTTTTCATAATCAAAATCAC
62[83]	AGCCGCCGCCAGCATTGACAG
63[105]	TTCCCAGTCACGACGTTGTAA
63[147]	TTCAGAGGTGGAGCCGCCACG
63[189]	AACAATCGGCGAAACGTACAG
63[63]	GGGATGTGCTGCAAGGCGATT

Start	Sequence (5' - 3')
7[154]	TCACCGTCCGCCACCCTGGGATAGGTCAATCTGCCAGGTGTA
11[182]	AACTAAAGAGGTGATACGAAGAAACGGGTAAAATAGTGATCG
11[203]	TAATTTTTCGGTTTCGAAAGATTTCATGAGGAAGTGAAA
9[224]	AACCATCAACCGATACGGCTACATTTTT
11[77]	ATACATTTTTCATTATAAAGCAAATTAAGCAATAACATCCAATAAATCA
9[119]	TAATCAGGGGTAATCGTAAAACTAGCAT
11[224]	CTCCAAACAAAAGGCTAAAACGGCTTTGAGGACTAGGTAGCAATATTCG
6[62]	CGTCCAATACTGCGAAAACGACCTGACTATTATAGAAGA
2[125]	CAGTGAAAGGCCGGACCGTTCAATTCGCATTGTAA
4[139]	TCATACAACTCCAGGATTCTCCGTGGAATACATCAACATTAA
57[154]	GGTGCCGGCTCAGTACCAGCGTTACTCCTCAAGAGCAACGCCGAACCCA
57[175]	CGCCATTGATTTGTATGCCACATCCTCATTTTCAGTAACACTGAGTTAC
0[209]	CCAAATCGACCTTCGAACCGAACTGACCAATCCGCCAGCGGACGAATAA
2[237]	TTTTTGGCGCAGACGGTCAATTACTTAGCTAAACATGAAAAT
3[17]	TTTTTTAAAGGAATTACGTAGCTCAAGGATTA
2[62]	TAGGAATACCACATCAACACTTAATTGCTTGCTCC
57[70]	CTTGATATGTAATATCAGAGCTGTTTAATTCGAGCAAGAGGTCATTTAG
57[91]	AATCCTCGGACCAAAAACATTTTATTTCAACGCATCGAGTAGTGGTCAA
3[109]	TCAAGACAGTGATTCAAAAGGGTGTTTAATTATTTTTT
0[125]	GAGATGGAGCCCGTATAAACAGTTAATGAGTAAATCCATCAA
2[160]	TAAGAGGCTGAGAACCTATTTCGGAACCACGAGAAGCATTCC
0[167]	TGCCCTGTATTATTCTGAACGGTGTACACAGTGAATAGTTAG
4[191]	GTAGTGTCGAAACTTTGCGCATAGGCTGGCTAACGTAAGTTTTGT
4[212]	TTATCCATGTCATAAGGATCAAGAGTAATCTCGGATATTTAGTAA
1[14]	TTTTTAACGAACTAACGGAACAAATCTAGTTTTAA
4[44]	CAAGTAAGAGTCAACTATTACAGGTAGAAAGCAGTCAGTACGGTG
0[62]	ATTATACATTCATCAGTTGTATTTTAAATTAAGAATCCATAT
2[97]	CCAAGGATAAAAATCCTGAGTAATGTGTTGTGAATAATTCTG
57[105]	CCAGAATGGAAAGCTGATAAAATCTACAAAACAGGCGGTTGA
6[139]	ACGACGAACCGTGCCGTTGGTGTAGATGAAGAACAAACGGCG
6[160]	AGCCCGGAATAGTTTCGCGGATAAGTGCAAGCCCAAGAACCG
9[182]	CTTAAACCCGCTTTTGCGGACCGCCACCCAGAGCCACCACTT
57[196]	CGCAACTCGCGAAACCTAAAAATCAGCTTACCGAT
57[210]	AGGGCGATCGGTGCACCCCCAGAATACAAGCCTTTTGACAAC
5[17]	TTTTTTAAAAGAAGTTTTTCCAACAGCATCAA
4[62]	CCAGACGACGATAATAGTAAACGAACCAAGCCCGA
9[77]	GCGCGAGGTAGCATTAACAAAAATCAGGCAAATATCGCGTGG
6[97]	GTAAAGAATTAGCATAAATCGGTTGTTACTGTTTAGGTGGCA
15[14]	TTTTTCTTATAAATCAAAAGAGAAATCGGCAAAATCC
27[21]	TTTTTACATAGCGATAGCTTACTTAGAAAAGGGATAGGCCACCGAGTAAAAGAG
25[21]	TTTTTTTTTAATGGAAACAGTTCATTTGGCAAATTGAAGAACTCAAACTATCGG
21[18]	TTTTTCCTTGCTGGTAAATCAATATC
19[18]	TTTTTTCTGTCCATCACAATTACCTT
15[39]	CCCTCAGAGCGGGAGCTCCGATTATCCTTGAAA
31[18]	TTTTTCAATCGTCTGAAATGGACCATTAAAAAGAGGTGAGGCGTACATTTTGACGCT
35[21]	TTTTTAGATAGAACCCTTCTGACATTCTAGTCACACGTTATTTACATTGGC
37[14]	TTTTTTGGAACAAGAGTCCACGAGTGTTGTTCCAGTT
39[21]	TTTTTAAGGGCGAAAAACCGCGTGGACTCCAACGTCA
40[41]	GAGTCTATCAGGGCGATGG
23[21]	TTTTTTGGTCAGTTCTGATTGTT

Start	Sequence (5’ - 3’)
49[21]	TTTTTTGGATTATATATCAAAAAGGAGCTTTTGCGGAACAAAGA
45[21]	TTTTTTATTTGCACAATTTTAAAAGT

Table S2. Functional sequences for the DNA origami.

Name	Start	Sequence (5’ - 3’)
Cholestrol-1	19[18]	TTTTTTCTGTCCATCACAATTACCTT ATAATAATAATAATA
Cholestrol-2	35[21]	TTTTTAGATAGAACCCTTCTGACATTCTAGTCACACGTTATTTACATTGGC ATAATAATAATAATA
Cholestrol-3	39[21]	TTTTTAAGGGCGAAAAACCGCGTGGACTCCAACGTCA ATAATAATAATAATA
Cholestrol-4	31[18]	TTTTTCAATCGTCTGAAATGGACCATTAAAAAGAGGTGAGGCGTACATTTTGACGCT ATAATAATAATAATA
Cholestrol-5	40[41]	GAGTCTATCAGGGCGATGG ATAATAATAATAATA
Cholestrol-6	49[21]	TTTTTTGGATTATATATCAAAAAGGAGCTTTTGCGGAACAAAGA ATAATAATAATAATA
Cholestrol-7	15[14]	TTTTTCTTATAAATCAAAAGAGAAATCGGCAAAATCC ATAATAATAATAATA
Cholestrol-8	45[21]	TTTTTTATTTGCACAATTTTAAAAGT ATAATAATAATAATA
Cholestrol-9	15[39]	CCCTCAGAGCGGGAGCTCCGATTATCCTTGAAA ATAATAATAATAATA
Cholestrol-10	21[18]	TTTTTCCTTGCTGGTAAATCAATATC ATAATAATAATAATA
Cholestrol-11	23[21]	TTTTTTGGTCAGTTCTGATTGTT ATAATAATAATAATA
Cholestrol-12	27[21]	TTTTTACATAGCGATAGCTTACTTAGAAAAGGGATAGGCCACCGAGTAAAAGAG ATAATAATAATAATA
Footholds-I-1-1	22[174]	GCGCCAAAGGGAAGGAATTATCACCGCAAACGTAGAGAAACG CAA TCA CAG GGT AAG TCT
Footholds- I -2-1	25[133]	ATAGCAGCACCGCTTACATCGCATCCTCCGGTCCG TTG TGA ACT GGT AAG TCT
Footholds- I -3-1	20[118]	CCAGCGGCAGTGTCACTGCCGATGCTGAGTCTCGTCGCTGAG GTA ATG AGC GGT AAG TCT
Footholds- I -4-1	23[98]	AGCGGCCAGAATGCTCTGCCAGCACGTCGCATCAGGCCCCCT CCA ACA ATT GGT AAG TCT
Footholds-I-1-2	14[160]	AGCAAGATGCAAATCCAATCGCCATTACAACGCAATTAAGAC CAA TCA CAG GGT AAG TCT
Footholds- I -2-2	15[133]	CCCACGCGCGGGGTATAACTATATGTAAGAAACGTTCAGCAGCGGAACG TTG TGA ACT GGT AAG TCT
Footholds- I -3-2	48[125]	CAAGTTTCGTGAGCTCATACCGCGCAGCCTCCGGCCTTGTAGAACGTAC GTA ATG AGC GGT AAG TCT
Footholds- I -4-2	14[97]	CGGTTGCCTGAGAGACTACCTTACCGAGTGCTCGTAATCGTT CCA ACA ATT GGT AAG TCT
Footholds-I-1-3	16[160]	AAGTTACCAGAAACTTACGGCTGTAGCTATCTTACCCGAACA CAA TCA CAG GGT AAG TCT
Footholds- I -2-3	26[149]	CCGATGCTGAGGTGTCCAGCATCAAACCAGCCGCAAGA TTG TGA ACT GGT AAG TCT
Footholds- I -3-3	27[119]	TTAGGTTTCACAGTACCAGCGTGGTGCTCGGCAGCACCGTGC GTA ATG AGC GGT AAG TCT
Footholds- I -4-3	16[97]	TCTGAGACGGGCAAAGAGTTGCAGCGGTATGAGCCAAAGGTT CCA ACA ATT GGT AAG TCT
Footholds-II-1-1	3[109]	TCAAGACAGTGATTCAAAAGGGTGTTTAATTATTTTTT CAA TCA CAG GGT AAG TCT
Footholds-II-2-1	0[125]	GAGATGGAGCCCGTATAAACAGTTAATGAGTAAATCCATCAA TTG TGA ACT GGT AAG TCT
Footholds-II-3-1	2[160]	TAAGAGGCTGAGAACCTATTTCGGAACCACGAGAAGCATTCC GTA ATG AGC GGT AAG TCT
Footholds-II-4-1	0[167]	TGCCCTGTATTATTCTGAACGGTGTACACAGTGAATAGTTAG CCA ACA ATT GGT AAG TCT
Footholds-II-1-2	2[125]	CAGTGAAAGGCCGGACCGTTCAATTCGCATTGTAA CAA TCA CAG GGT AAG TCT
Footholds-II-2-2	4[139]	TCATACAACTCCAGGATTCTCCGTGGAATACATCAACATTAA TTG TGA ACT GGT AAG TCT
Footholds-II-3-2	57[154]	GGTGCCGGCTCAGTACCAGCGTTACTCCTCAAGAGCAACGCCGAACCCA GTA ATG AGC GGT AAG TCT

Name	Start	Sequence (5’ - 3’)
Footholds-II-4-2	57[175]	CGCCATTGATTTGTATGCCACATCCTCATTTTCAGTAACACTGAGTTAC CCA ACA ATT GGT AAG TCT
Footholds-II-1-3	57[105]	CCAGAATGGAAAGCTGATAAAATCTACAAAACAGGCGGTTGA CAA TCA CAG GGT AAG TCT
Footholds-II-2-3	6[139]	ACGACGAACCGTGCCGTTGGTGTAGATGAAGAACAAACGGCG TTG TGA ACT GGT AAG TCT
Footholds-II-3-3	6[160]	AGCCCGGAATAGTTTCGCGGATAAGTGCAAGCCCAAGAACCG GTA ATG AGC GGT AAG TCT
Footholds-II-4-3	9[182]	CTTAAACCCGCTTTTGCGGACCGCCACCCAGAGCCACCACTT CCA ACA ATT GGT AAG TCT
Fluo-1	62[125]	ATGCATCCCCATAACCCATT TTTT CAGAGCCACCACCCTCAGAGC
Fluo-2	62[167]	ATGCATCCCCATAACCCATT TTTT AACCGCCTCCCTCAGAGCCGC
Fluo-3	62[209]	ATGCATCCCCATAACCCATT TTTT TCTTTTCATAATCAAAATCAC
Fluo-4	62[83]	ATGCATCCCCATAACCCATT TTTT AGCCGCCGCCAGCATTGACAG
Fluo-5	63[105]	ATGCATCCCCATAACCCATT TTTT TTCCCAGTCACGACGTTGTAA
Fluo-6	63[147]	ATGCATCCCCATAACCCATT TTTT TTCAGAGGTGGAGCCGCCACG
Fluo-7	63[189]	ATGCATCCCCATAACCCATT TTTT AACAATCGGCGAAACGTACAG
Fluo-8	63[63]	ATGCATCCCCATAACCCATT TTTT GGGATGTGCTGCAAGGCGATT
HCR-initiator-1	11[35]	GAGAGTAAGCAAACGCCAGAGCATAAATATTCATTGAATC TT GCATTTCCTTCTGAGAAGGGCAAACGACGGGAGGAG
HCR-initiator-2	13[35]	ATATGCAAATGCTGAGGCATAAATAGCGAGAGGCTTTTGC TT GCATTTCCTTCTGAGAAGGGCAAACGACGGGAGGAG
HCR-initiator-3	7[17]	TTTTTCCCCTCAAATGCCAAAGCGGA TT GCATTTCCTTCTGAGAAGGGCAAACGACGGGAGGAG
HCR-initiator-4	1[39]	TTTTTCCCCTCAAATGCCAAAGCGGA TT GCATTTCCTTCTGAGAAGGGCAAACGACGGGAGGAG
RNA-activator-1	11[35]	GAGAGTAAGCAAACGCCAGAGCATAAATATTCATTGAATC TTT CGCTAATACGACTCACTATA
RNA-activator-2	13[35]	ATATGCAAATGCTGAGGCATAAATAGCGAGAGGCTTTTGC TTT CGCTAATACGACTCACTATA
RNA-activator-3	7[17]	TTTTTCCCCTCAAATGCCAAAGCGGA TTT CGCTAATACGACTCACTATA
RNA-activator-4	1[39]	TTAATGCAGATACATAACGCCA TTT CGCTAATACGACTCACTATA
DNAzyme-1	11[35]	GAGAGTAAGCAAACGCCAGAGCATAAATATTCATTGAATC TTT TGCCCAGGG AGGCTAGCTACAACGA GAGGAAACCTT
DNAzyme-2	13[35]	ATATGCAAATGCTGAGGCATAAATAGCGAGAGGCTTTTGC TTT TGCCCAGGG AGGCTAGCTACAACGA GAGGAAACCTT
DNAzyme-3	7[17]	TTTTTCCCCTCAAATGCCAAAGCGGA TTT TGCCCAGGG AGGCTAGCTACAACGA GAGGAAACCTT
DNAzyme-4	1[39]	TTAATGCAGATACATAACGCCA TTT TGCCCAGGG AGGCTAGCTACAACGA GAGGAAACCTT

Table S3. Functional sequences for DOS and DOS-directed reactions in GUVs.

Name	Sequence (5’ - 3’)
B1	CCT TAT ATC CT CTTACC CTGTGATT G
B2	ATTCTTCAATC CTTACC AGTTCACA A
B3	ATCTAGTTATG CTTACC GCTCATTA C
B4	CATTCATGTAA CTTACC AATTGTTG G
R1	CAATCACAGGGTAA GAGGATATAAGG
R2	TTGTGAACTGGTAA GGATTGAAGAAT
R3	GTAATGAGCGGTAA GCATAACTAGAT
R4	CCAACAATTGGTAA GTTACATGAATG
AuNP-SH	[ThiC6]- TTTTGACTTACC
DOS-Cholestrol	[CholTEG]- TATTATTATTATTAT

Name	Sequence (5’ - 3’)
DOS-Atto488	AATGGGTTATGGGGATGCAT- [Atto488]
H1	[Cyanine3]-GAGAAGGGCAAACGACGGGAGGAGTTCTTTCTTACGCTCCTCCCGTCGTTTGCCCTTCTCAGAAGGAAATGC
H2	CGTAAGAAAGAACTCCTCCCGTCGTTTGCCCTTCTCGCATTTCCTTCTGAGAAGGGCAAACGACGGGAGGAG
RNA template	GGAGCTCACACTCTACTCAACAGTAGCGAACTACTGGACCCGTCCTTCACCCTATAGTGAGTCGTATTAGCGAGTATAGGG
RNA substrate	[Cyanine3]-AAGGTTTCCTCR(g)R(u)CCCTGGGCA-[BHQ2]

Table S4. Sequences of DNA origami component of the DOS variant with blocked needle channels.

Start	Sequence (5’ - 3’)
0[153]	GTTGGGAAGAAAAAGGCTGCCAGTTTGAGGGGACGTCAGGAC
0[83]	CAGTTGATTTATGACCCTGTAATACTTT
0[97]	GTAGAAAATCTATGTTGTGTACCAAATGGGAACAA
1[105]	GGTAATCGTAAAACAACGGAATGAGCCC
1[126]	GATTGACCGTAATGTCTACGTCGTCCAG
1[14]	TTTTTGCATAGTAAGAGCAACCAAAAGGAACGAAC
1[168]	AGCAAGCCCAATAGAGAACTGTAAAGGA
1[189]	CTGAGGCTTGCAGGGAATTACACGGTCA
1[217]	ATGCCACAATTTCAGAACTGACCAACTTTGAAATTTTTTT
1[42]	TAAATATTCATTGAACTAATGTCAAGTTCTCGTAAAATATGAGATAAAT
1[63]	CGGTGTCTGGAAGTGATTTAGCTACCTT
2[146]	TGCATATAGGTCACGTTGGGAACTATTCTGAAACATGAAAGTATTA
2[195]	CGCTTTATATTCACCCATGTACCGTAATTTTCAGACAGTATCGGCCTCG
2[237]	TTTTTAGTAGTACGAAGGCACCAACCTAAACGGGTTAAAGGC
2[48]	ATGCTGCGGTACCTTTAATTGCTCCTTTGTCTACCCCCCTCAA
2[90]	CCTTTAAAACGTTCTAGCTGATAAATTATACGACGGGAGAAG
3[105]	GATGTACCCCGGTTGCGTCATGGTAGCC
3[154]	AGATCGCGTTTCAGCGCTGAGACTCCTCCATCGTAGCAAGCG
3[16]	TTTTTTTTTTACCAGACGAACAGGTCTTTTTTTCCACAGC
3[189]	GATTGCGGGATCGTACGAGGCACCTGCT
3[49]	CAGTTCATAAGACCGCAATGGAGGCACCGAGCTTTTCAGCTC
3[63]	ATGTTGATTCCCAATCATCAAGCCTTAG
3[91]	AACGCAACCGGCAAGATAGCTCTGCTCGATTTAGCTTGAGAG
4[139]	TCATACATGGCTCGGAAATGCCCCCTGCGAAGGATTAGG
4[237]	TTTTTGAAACACCAGGGCCTCTTTTTTT
4[48]	TGACCTAGCAAAGCGAACCAGAGGTCATTGTTTAAACGAGAA
5[105]	TCCCAAAAACAGGATGTATTCGTCTACG
5[16]	TTTTTTTAGCGAGAGGCTTGCGTTTTTTTTTTTATCGCCA
5[175]	GCCTGTAGGTTTATCAGCTGTAAAAAAAAAAGGCT
5[189]	GCAGACAGCATCGGCGAAATCTGTATCA
5[63]	AAATTTAGTTTGACCAAATTTCCAAGGT
6[146]	ATAGGAGTGTACTGGTAATGAGTTTGAGTAACAGTACCAGGCGGAT
6[195]	CAACGACAACAAATTCCACAGACAGCAACCGCCTTTAGTACCGCCACGA
6[237]	TTTTTGCTTGCCTGACCCCCAGCGATAAAGACTCGAGGGTAG
6[48]	CCCTGATGTAAGAGGAAGCCCGATGGCTTATGTAGGTCTTTA
6[90]	ATGCCAGAGTCAAAAGGGTGATTTGAGAATCCTTTAAATGCA
7[140]	TATAGCCCGCCTCAGAACCGCAAAGTTTTGCCGTCGAGAGGG

Start	Sequence (5' - 3')
7[16]	TTTTTTTGCCAGAGGGGGTAATAGTAAAACTATTAAATTG
7[182]	CCGACAATGGCTACAGAGGCTCAACGGAGACAGCT
7[56]	CTGAATATACAAATGGAGCAATAAAGCCTCTGAGTAAAAGGC
7[98]	TATCAGGTCTATTTAACATTAAATGTGAGCAAGTTTTATAAG
8[139]	TTGATAACGGGGTCGCCGCTTCGTATAAAAGCAAT
8[188]	TGATACCGATAGGTTAGCGTATATGCTTTCGAGGT
8[242]	TTTTTTTACGTAACAAAGCTGCTCAGCGCGAAAATACCGG
8[90]	TGTAGGTAGAATTCAGGCCGGCTTCTAGTATGATACAGGACCGATTCAT
9[112]	AAAGCCAGCTCCAACATTGTATAAGCAAAATTGATTTGTTA
9[161]	CAGACGTACGATCTCACCCTCAGCCTCATATTGCG
9[182]	GAATTTCTTAAATTCGTTTAATTGTATCGCCCATC
9[203]	TCGCCTGCAAAGTATTGAGGACTTATACCAATTCAGTGAATAAGTTTTT
9[21]	TTTTTTTATCAAAAAGATTTAGCAAAAGAAGTTTTTTTTTTT
9[217]	ATATTCATTACCCAAATCATTTTTTT
9[70]	TCAGAATTAGATCATTTAGATACATTTCGATGCGCTATATTT
10[146]	TTTTGCTGGTGTATCACCGTACTTTCCGACCAGGA
10[216]	CAAGAAAACCCAGGCGCATAGGCTGGCTGACCTTTTTTTT
10[242]	TTTTTTTTCATCAAGAGTATTCTGACGATTTTT
11[112]	GCTCTGGCCTTTTTCTAGCAGTCTCTGAATTTACCGTTCCAGTAC
11[161]	GCTAAACAAATGAAGGACCCTCAACAAC
11[182]	CCAAAAGGAGCCCGGCAATTTTTTCACGGTTAACC
11[203]	CCATGTTATTGTGTAATTTTCATAAAACACTCATCATCTTGA
11[21]	TTTTTTTAATTCGAGCTTCGTCATAAAAACCAAAATTTTTTTT
11[70]	CATACAGGCAAGGGTCTTTCACAAACAAATA
11[91]	CATAGAGGGTTTGCAATATTAAAGCCAGAAT
12[132]	TTTCGGACGTAAGCTAATAAAACGAACTTACGGCG
12[174]	TCTCCCTGAACAACGCTCATTATACCAGACGGGAT
12[216]	ACAGATTGGGAACCACTTTAATCATTGTGAGTAAAATACGTA
12[242]	TTTTTTTGAGGACAGATGAGGCAGAACGTTTTT
13[112]	TCAAAAATAAATAAAGCATGTCAATCATAGAATGGAACGCCA
13[161]	TAGAAAGTTCAACAACCACCCTCACACTGAGTTTCTTGAAAA
13[182]	ATTGCGAATAATAGCTTATGCGATTTTAGAGGTCG
13[203]	ATCATAAAGCCGGACATTTCCATTAAAACGAAAGAACGGTGT
13[21]	TTTTTTTAGGATTAGAGAGAATTATCATAACCCTCGTTTTTTT
13[70]	AACATCCAATACCATCATTCCATATAACAGCAAAGTAGCATT
14[160]	AGCAAGATGCAAATCCAATCGCCATTACAACGCAATTAAGAC
14[97]	CGGTTGCCTGAGAGACTACCTTACCGAGTGCTCGTAATCGTT
15[133]	CCCACGCGCGGGGTATAACTATATGTAAGAAACGTTCAGCAGCGGAACG
15[14]	TTTTTCTTATAAATCAAAAGAGAAATCGGCAAAATCC
15[196]	ACATAAAAATTGAGCAAATATATTTTAGACTAGAACTAACGAGCTATTT
15[217]	TTAGACGATCAGAGCATCTTCTGACCTAATAAACACCTAATTAGCCTTA
15[238]	ACACCCTAGAGGGTTGGTTTGAAATACCTAAATAATAAACAGCCGACTT
15[39]	CCCTCAGAGCGGGAGCTCCGATTATCCTTGAAA
15[70]	CGCCTGGGGTTTGCGAATTTATCAAAATCATGGTCTGGTTTTGCGGTTT
16[118]	GGCTGGTATCCCTTTGAGGATTCGCGTC
16[139]	ATGCCAAGGTCTGGCACCAATGAAACCCGGGT
16[160]	AAGTTACCAGAAACTTACGGCTGTAGCTATCTTACCCGAACA
16[181]	AAGTAAGGGAATACAATCACCGCCATTT
16[202]	AATGAAAACCAACGAAAGCCTGTTTAAGACTT
16[223]	GATTTTTTCCAGAGCCGGAATACCAGTA

Start	Sequence (5’ - 3’)
16[244]	ATCCCAATACAAAAGGCGTTAAACAGTAGGGCTTAGCCATATTAGCGAAACCAAGT
16[55]	TTTCCTCAGGAACGTTAATTATCAATATATGTGAGAATT
16[76]	TTGCCCTGCCAGGGATCTTCTGTAAATCCAATAGTCCCAGCA
16[97]	TCTGAGACGGGCAAAGAGTTGCAGCGGTATGAGCCAAAGGTT
18[107]	GCACATAAACAATGGGTGGGTCACTGTTGCCTCGCACTCTCCGGC
18[118]	TGGTGTGGTTACCTGGGGGTTGGCGGGCCGTTTTCAAGCGCCTGTGCAC
18[139]	TGCCGGACAGAGCAACATAAAAAAATGGCTCATCG
18[160]	TCCTTATTACGCAACAGGAAACCGAGGACATT
18[170]	TGATAATAACCAGATAGCGAAGCCCTTTTTAAAGAGCAAAGAACG
18[181]	AAGAACTATACATATAAAGGTGTAAATATTGACGGTATATGGTTTACCA
18[202]	TGCACCCAAATCAGCCAGTAATAAGAATAGGTATC
18[223]	AATCAAGTCCGGTATTAGGCAAGTAATTCTGTCCAAACAATATCTTTCC
18[244]	GCGGGAGGGCGTTTTTAACAAACAATAAACAACATTGCAGAAACGGGTA
18[44]	GTGTTTAGACGTTAGAAGAGTACTATGGTTGATCCTGTACGCTGA
18[55]	TTTTATAGTAGCAATACATTTTGCAAAAGAAGATGTTGC
18[76]	GCGTATTCCAGCTGTATCAAGAAAACAATGAATAACCTTGAGGGTCATA
18[97]	AAGCGCGGGGAGAGTCTTTTCACCAGTTCTCG
19[18]	TTTTTTCTGTCCATCACAATTACCTT
20[118]	CCAGCGGCAGTGTCACTGCCGATGCTGAGTCTCGTCGCTGAG
20[139]	TTTTTTCTTGCCGTGAAGGGTAAAGTTAACCCCGTAAAAAAA
20[160]	CAAAGACACCACCGATAGTATGTTAGCAGTTGGGCCAACCGA
20[181]	TGGCAACATAGAAAATTCAATTTACGAGTCATTACCGCGCGG
20[202]	GTAGGAACATGTAGATAATATCCCATCCAAGAATA
20[223]	CAAGCCGTCGGCTGGATAAGTCCTGAACAAGGTAAGAGGCATAATT
20[244]	ACCGCACTCCAAGACGCGCCTGTTTATCGACGACGCGCCAACAACG
20[55]	CACTTGCCCAGCAGACCTCAAAGCGTAAGAATACGAATATTT
20[76]	AACCTGTAACACATTAATTGCGTTGCGC
20[97]	GCGCCCGCTTTCCAGAATCGGCCAACCGCGCTCACAGTGTAA
21[154]	AAGTTTAAGGGCGACAGCCTTTAGTGATTCCGGCAAAGGAAT
21[18]	TTTTTCCTTGCTGGTAAATCAATATC
21[210]	TCAATAATTTTTATAGGCTTAATTAGTTGCGTCTTTGTTTAAAAGCGCA
21[231]	TTATCATTCATCGAAACGCGAGTTTTGATGCCAGTTCCAAATTAACTGA
21[91]	TCACTATTGGGGTGCCTAATGAGCATAAAATTCCACTGT
22[111]	TCTGTGGTGCTGCCCAGACGATCCAGCGTGCCGGTATGCCGGTTCA
22[174]	GCGCCAAAGGGAAGGAATTATCACCGCAAACGTAGAGAAACG
22[58]	TGACAAATGAAAAATCTCGTAGTAATAACAT
22[76]	TAACTAGCATCACCATGAAACAAACACGAGCCGGAAGTGAGC
23[140]	GCCGCACAGGCGTTGGTTGTGGCTCATTAAGG
23[161]	TTGAGGGAGACAAATTTTGTCACAATCAATATAAAAAAATACGGCA
23[196]	TAAAGTACCGACAAAAGAAAAAAACCAA
23[21]	TTTTTTGGTCAGTTCTGATTGTT
23[39]	AATACCCTCATATCCAGAACAAAGCTGAGTAAACCGTTATCA
23[98]	AGCGGCCAGAATGCTCTGCCAGCACGTCGCATCAGGCCCCCT
24[44]	ATTACATAAAATTTTCCGATTAAGTTGATGGAAGC
24[58]	AATTACTTCTTTGATTGGGCCTGAGAAGTGT
25[105]	CTGTTCTCCCCGGGTTTTAACCAATCCGCCGGGCG
25[133]	ATAGCAGCACCGCTTACATCGCATCCTCCGGTCCG
25[168]	GACTTGAAGTAGCACAAGACAAGAAACAATGAAAT
25[196]	ATATGCGTGAAGGGATAGCTC
25[21]	TTTTTTTTTAATGGAAACAGTTCATTTGGCAAATTGAAGAACTCAAACTATCGG

Start	Sequence (5’ - 3’)
25[214]	CTTCATAATTTTAATTTAGATAACCCACAAGAACAGGGCGTCAAA
25[235]	CTCAATAAGAAATTTAAAATTGAGCGCTAATGGAGAATAAGAAAC
25[88]	GTGTCGTAATCATAGGTAAGCGGTCCACGCTCCCTGAGCAGCTGA
26[149]	CCGATGCTGAGGTGTCCAGCATCAAACCAGCCGCAAGA
26[156]	AGCTGCCGCCGATCAAACTTAAATTTTAATCAGTAGCGACAGAAT
26[93]	AATAAATTGTCGCGTTTTCATCGG
27[119]	TTAGGTTTCACAGTACCAGCGTGGTGCTCGGCAGCACCGTGC
27[130]	TATCATTGCAGGCGCTTCTCGGTGGTGCCAT
27[182]	CGAGAAACCAGCAACCATCCTGAATCTTATAGCAGCCTTTAA
27[193]	TTTTTAAGCCCAATAATAGACAGAGAGAATA
27[21]	TTTTTACATAGCGATAGCTTACTTAGAAAAGGGATAGGCCACCGAGTAAAAGAG
27[56]	GAAGAGTGTCGCTAGTACGCCAGAATGCTCCGTATAACGTGC
29[19]	AACGCTCATGGAAATACCATTGCAACAGGAAA
29[49]	ACCGCCTCACGCTGTATGGCTATTAGTCGAACTGAACGAACCACTTTTT
30[69]	TTTTTCAGCAGAAGATAAAACATACCGATAGCCCT
31[18]	TTTTTCAATCGTCTGAAATGGACCATTAAAAAGAGGTGAGGCGTACATTTTGACGCT
32[48]	AAAACATCGACCAGAGTATTACCGCCAGCCGTCAGTATTAAC
33[21]	TTTTTAGATTCACCGGCCAACAGTTTTT
34[55]	TTGAAATAAAAGGGACCTGAAATATCAACAACAGTAACA
35[21]	TTTTTAGATAGAACCCTTCTGACATTCTAGTCACACGTTATTTACATTGGC
37[14]	TTTTTTGGAACAAGAGTCCACGAGTGTTGTTCCAGTT
37[49]	GCCGCGCGCTACAGGGCGCGAGGAAAGCGCGAAAG
39[21]	TTTTTAAGGGCGAAAAACCGCGTGGACTCCAACGTCA
39[49]	AGGGCGCGAAGAAACGGCGAACGTGGCGAGTTTTT
40[41]	GAGTCTATCAGGGCGATGG
40[69]	TTTTTAAAGGAAGGTGGCAAGACGCTGCGCGTAACCACCAACGGGCGCT
41[18]	TTTTTCCCACTACGTGAACCATGGTAGGGTTTATTAAAGACACCC
42[69]	TTTTTGGGAGCCCCCGATTTAAAATCGG
43[18]	TTTTTTTGGGGTCGAGGTGCCGTATGGTTCCATAG
43[46]	ACTGAGCTTGACCACCCAAATCAAGTTTTTTTTT
43[56]	AACCCTAGGCGAAACTTTGACGAGCAAC
44[44]	GAAAGCGGATTCGCCTGATCCTTTCAAACAATTCGACAACTTTT
45[21]	TTTTTTATTTGCACAATTTTAAAAGT
46[48]	AATAAAAATAAAGAGAATATACAGTAACAGTACCTGAGAAAC
47[14]	TTTTTAGGTTTAACGTCAGATAATTGCGTAGATTTTCTTTTT
48[118]	GCCTTTAGCGTCAGACTGTAGTACGTGC
48[125]	CAAGTTTCGTGAGCTCATACCGCGCAGCCTCCGGCCTTGTAGAACGTAC
48[181]	AAAAGAGACGCAGAAACAGCGAGTCACC
48[188]	TCACGGAGGGAATTTATTCATAACCAATAGCAAGCAGCTACAATTTTAA
48[223]	AGAGATAGACTTTCTCCGTGGTTATACATTTCGAGATATAGATTTCATC
48[230]	ATTTGTGTAAAGCCATGTAATTTCTAAGGAACAAG
48[83]	CATTTTCCTGTTTCCACAACACATTAATGTCGGGA
49[21]	TTTTTTGGATTATATATCAAAAAGGAGCTTTTGCGGAACAAAGA
50[48]	AGGAAAAATAATGGAACGCAGAGGCGAACAATTACCTGAGAA
51[14]	TTTTTGATTATCAGATGATGGTTATCATCATATTCCCACCAG
52[64]	TTTTAAATATCTTTAGGAGCACTAACAAAACAATTCATCTTGAGGAAGGTTATCTATTTT
54[64]	TTTTATAATACATTTGAGGATTTAGAAGCAGGCTAATAGATTAGGTTA
55[17]	TTTTTTTTGAGTAACATTATTATTAGACTTTAGCCCGAA
56[41]	CGTTATTGTAAAACCCTACCACTTCTGATATAATCGGCA
56[68]	TTTTTCGTATTAAATTGCTTGTTACAAAATCGGGAGCCGTCAATAGTTTT

Start	Sequence (5’ - 3’)
57[112]	GGAAAGCATCAGAAAAGCCTGGAGTTAAATCA
57[147]	CGCTTCTGGTGCCGCAGCCAGCTCAGGATTTTCTGTACGGGG
57[175]	CGCCATTCCAGTACAAACTGAGCCACTCGAAACCAGGCAAAG
57[196]	CGCAACTCTCAGCAGCGAACCACGCACACGCCATTCAGGCTG
57[210]	AGGGCGATCGGTGCAAAGAATACACTGAGGAAGCCGTTGGGA
57[63]	TTTTTTTCTTGATAGCGAACGAGTAGCATGAAAAGGTGG
57[91]	AATCCTCAAAAATTTTTAGAATCTCAAATCAC
62[125]	CAGAGCCACCACCCTCAGAGC
62[167]	AACCGCCTCCCTCAGAGCCGC
62[209]	TCTTTTCATAATCAAAATCAC
62[83]	AGCCGCCGCCAGCATTGACAG
63[105]	TTCCCAGTCACGACGTTGTAA
63[147]	TTCAGAGGTGGAGCCGCCACG
63[189]	AACAATCGGCGAAACGTACAG
63[63]	GGGATGTGCTGCAAGGCGATT
65[119]	CTCGTACAGTGTTCATCAAATTGTAAACCAATCTAGCATTAA
65[140]	CGTATCGCAGAACTCGTCTTTC
65[35]	GAGTACTGCAAAGCGGATTGCTTTTTTT
65[49]	AGATATGCGAGCAATGTTTAGAGCTTTAGTCAGAATCGTCTCAGACTTC
65[77]	CCCGTAGAAAGCAAAATTATCAATAACCCCGGTTCGGGGCGC
65[91]	TGGGAGCTACGTCGGTTAGATCTACATG
67[105]	TACACAGTTTTTAACAAACCTCAACATTATTACAGTGTGAAC
67[119]	GCCTCGCACAGTCCAGAACCCGTTTTGATGATACACAGTGCCTCTAGTC
67[140]	CGAGTTCACGGATTGGGATTTT
67[35]	TTATCAGAAATATCTTGACTGGAATAAATCAAAAATGCGGAA
67[42]	AATCATGGGAAGCATTTGCAGCAGATACATAACGCACCGTCA
67[63]	GTTGCACTTCTACTGGCCGGGGAATACCACATTCAATAAGTA
67[77]	TACTCAAAGACAGGCAAAAGCTAAACCCTCATATATATTGAA
69[119]	AGATACGACCTTTCGATCCGTGGTGTAGATGGGCGAAGACTAAGTCATG
69[140]	ATCTCGTCCAGGTGAGTGAGAA
69[35]	GACCACCAACTCCACGCAATACTTTAAA
69[77]	ATCACGCTTCAAAATTTGTACCAATTTC

Table S5. Mini-scaffolds of DNA origami component of the DOS variant with blocked needle channels.

Name	Sequence (5’ - 3’)
1	GTTCTGCGATACGGACTAGAAAGCGGCGTACGAGCGTAGACTAGATTGCGACGTA
2	ATCCGTGAACTCGCATGACTATTGCTTGCGAGGCGGCTACCCTGTGTACGAGCAG
3	ACCTGGACGAGATCTGGACGGCTTACGCGTATCTGGGCTCAAGGTTTGTACACAA
4	GCTCCCAGAATTCTCTACGGGACCTTGGGAACCGGTTGCTCG
5	AGCTATCCTAGAAGTTGAGTACTAAGGCGTGCAACGGTGCCT
6	CATAGATGGTCCTGGCGTGATAAGGTAGCCCGGCCTTACGAG
7	CATATCTGAGACGAAGTACTCTGGCGATTTTTTTT
8	CCATTGCCATGATTCTGATAAGCTGTGGTTTTTTT
9	AACTTGACTGCAAAGGTGGTCGTTCGTTTTTTTTT

References

1. Burns, J. R., Seifert, A., Fertig, N. & Howorka, S. A biomimetic DNA-based channel for the ligand-controlled transport of charged molecular cargo across a biological membrane. *Nat. Nanotechnol.* **11**, 152-156, (2016).
2. Ke, Y., Voigt, N. V., Gothelf, K. V. & Shih, W. M. Multilayer DNA Origami Packed on Hexagonal and Hybrid Lattices. *J. Am. Chem. Soc.* **134**, 1770-1774, (2012).
3. Wickham, S. F. J. *et al.* Complex multicomponent patterns rendered on a 3D DNA-barrel pegboard. *Nat. Commun.* **11**, 5768, (2020).
4. Jo, S., Kim, T., Iyer, V. G. & Im, W. CHARMM-GUI: A web-based graphical user interface for CHARMM. *J. Comput. Chem.* **29**, 1859-1865, (2008).
5. Jo, S. *et al.* CHARMM-GUI 10 years for biomolecular modeling and simulation. *J. Comput. Chem.* **38**, 1114-1124, (2017).
6. Jo, S., Lim, J. B., Klauda, J. B. & Im, W. CHARMM-GUI Membrane Builder for Mixed Bilayers and Its Application to Yeast Membranes. *Biophys. J.* **97**, 50-58, (2009).
7. Jorgensen, W. L., Chandrasekhar, J., Madura, J. D., Impey, R. W. & Klein, M. L. Comparison of simple potential functions for simulating liquid water. *J. Chem. Phys.* **79**, 926-935, (1983).
8. Abraham, M. J. *et al.* GROMACS: High performance molecular simulations through multi-level parallelism from laptops to supercomputers. *SoftwareX* **1-2**, 19-25, (2015).
9. Van Der Spoel, D. *et al.* GROMACS: Fast, flexible, and free. *J. Comput. Chem.* **26**, 1701-1718, (2005).
10. Klauda, J. B. *et al.* Update of the CHARMM All-Atom Additive Force Field for Lipids: Validation on Six Lipid Types. *J. Phys. Chem. B.* **114**, 7830-7843, (2010).
11. Bussi, G., Donadio, D. & Parrinello, M. Canonical sampling through velocity rescaling. *J. Chem. Phys.* **126**, (2007).
12. Bernetti, M. & Bussi, G. Pressure control using stochastic cell rescaling. *J. Chem. Phys.* **153**, (2020).
13. Hess, B. P-LINCS: A Parallel Linear Constraint Solver for Molecular Simulation. *J. Chem. Theory Comput.* **4**, 116-122, (2008).
14. Essmann, U. *et al.* A smooth particle mesh Ewald method. *J. Chem. Phys.* **103**, 8577-8593, (1995).
